# Repeatability and reproducibility assessment in a large-scale population-based microbiota study: case study on human milk microbiota

**DOI:** 10.1186/s40168-020-00998-4

**Published:** 2021-02-10

**Authors:** Shirin Moossavi, Kelsey Fehr, Ehsan Khafipour, Meghan B. Azad

**Affiliations:** 1 Department of Medical Microbiology and Infectious Diseases, University of Manitoba, Winnipeg, MB Canada; 2 Children’s Hospital Research Institute of Manitoba, Winnipeg, MB Canada; 3 Developmental Origins of Chronic Diseases in Children Network (DEVOTION), Winnipeg, MB Canada; 4 Digestive Oncology Research Center, Digestive Disease Research Institute, Tehran University of Medical Sciences, Tehran, Iran; 5 Department of Physiology and Pharmacology & Mechanical and Manufacturing Engineering, University of Calgary, Calgary, AB Canada; 6 Department of Pediatrics and Child Health, University of Manitoba, Winnipeg, MB Canada; 7 Department of Animal Science, University of Manitoba, Winnipeg, MB Canada; 8 Microbiome Research and Technical Support, Cargill Animal Nutrition, Diamond V brand, Cedar Rapids, USA

**Keywords:** Decontam, Reagent contaminant, Batch variation, Repeatability, Reproducibility, Microbiome, Milk microbiota, Human milk, CHILD cohort

## Abstract

**Background:**

Quality control including assessment of batch variabilities and confirmation of repeatability and reproducibility are integral component of high throughput omics studies including microbiome research. Batch effects can mask true biological results and/or result in irreproducible conclusions and interpretations. Low biomass samples in microbiome research are prone to reagent contamination; yet, quality control procedures for low biomass samples in large-scale microbiome studies are not well established.

**Results:**

In this study, we have proposed a framework for an in-depth step-by-step approach to address this gap. The framework consists of three independent stages: (1) verification of sequencing accuracy by assessing technical repeatability and reproducibility of the results using mock communities and biological controls; (2) contaminant removal and batch variability correction by applying a two-tier strategy using statistical algorithms (e.g. *decontam*) followed by comparison of the data structure between batches; and (3) corroborating the repeatability and reproducibility of microbiome composition and downstream statistical analysis. Using this approach on the milk microbiota data from the CHILD Cohort generated in two batches (extracted and sequenced in 2016 and 2019), we were able to identify potential reagent contaminants that were missed with standard algorithms and substantially reduce contaminant-induced batch variability. Additionally, we confirmed the repeatability and reproducibility of our results in each batch before merging them for downstream analysis.

**Conclusion:**

This study provides important insight to advance quality control efforts in low biomass microbiome research. Within-study quality control that takes advantage of the data structure (i.e. differential prevalence of contaminants between batches) would enhance the overall reliability and reproducibility of research in this field.

**Video abstract**

**Supplementary Information:**

The online version contains supplementary material available at 10.1186/s40168-020-00998-4.

## Background

Quality control of microbiome studies has been an integral component of pioneering projects including the Human Microbiome Project [1]. The Microbiome Quality Control Project (MQCP) focused on identifying sources of variability in 16S rRNA gene microbiota profiling across different laboratories, but batch-to-batch variability was not assessed [2]. As microbiome studies expand in sample size, we are facing the additional challenge of batch-to-batch variability in large-scale population-based studies. Additionally, repeatability and reproducibility of results are often unaddressed. Unlike other high-throughput methods such as transcriptomics and metabolomics [3, 4], these concepts are not well developed for microbiome studies.

“Batch effects are sub-groups of measurements that have qualitatively different behaviour across conditions and are unrelated to the biological or scientific variables in a study” [4]. Batch effects can mask true biological results and/or result in irreproducible conclusions and interpretations [4]. Potential sources of batch effects in microbiome research include heterogeneity in all aspects from sample collection to library preparation and bioinformatics processing [1] leading to technical variability. Reagent contaminants pose a major challenge in microbiome profiling of low biomass samples such as milk [5, 6] and could be an important source of non-technical batch variability even when all procedures are identical.

Repeatability is defined as obtaining the same results after re-running the same process on the same set of samples, while reproducibility refers to the ability to obtain similar results on a different set of samples [7]. Assessing repeatability and reproducibility is among the cornerstones of good scientific conduct and is being adopted in many areas of high-throughput experiments such as clinical genomics [8]. Studies have assessed the reproducibility of the microbiome profile as part of MQCP [2]. However, repeatability and reproducibility of results are not commonly assessed between batches. This process is important when combining results from multiple batches in large-scale microbiome projects. Therefore, the objective of this study was to perform extensive quality control and establish good practices using milk microbiome data generated in two batches (extracted and sequenced in 2016 and 2019). Additionally, we assessed and mitigated batch variability and examined repeatability and reproducibility in this dataset.

## Results

We studied a subset of 1194 mother-infant dyads in the CHILD Cohort Study [9]. Milk microbiota from a representative subset of 428 mothers was previously profiled in 2016 (batch 1) [10]. An additional set of 766 samples enriched in infant atopy and asthma was profiled in 2019 (batch 2). Experimental and bioinformatics procedures were identical for the two batches with the exception of DNA extraction kit lots. Some participant characteristics varied significantly between the batches (e.g. season of birth differed, and atopy/asthma were purposefully enriched in batch 2; Table S1) and thus some degree of true biological variability between batches was anticipated in the milk microbiota composition.

### Technical reproducibility

**Fig. 1:**
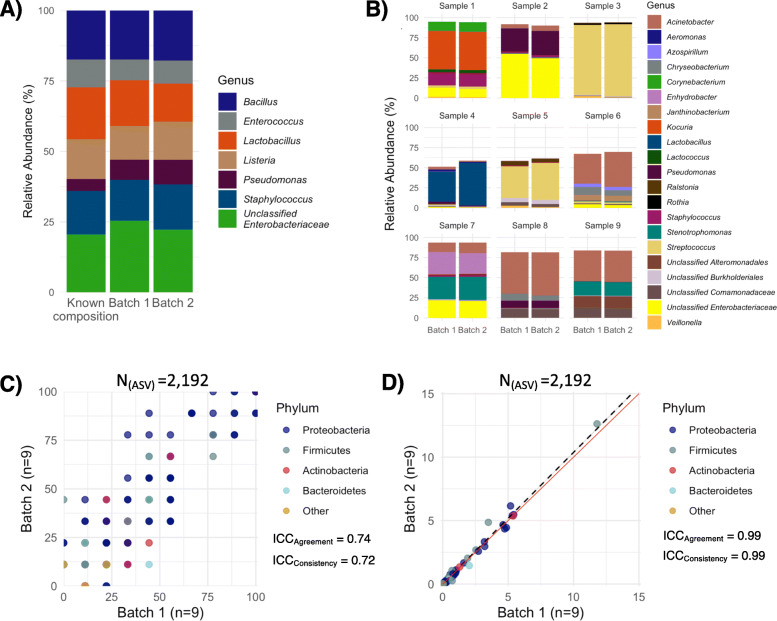
Sequencing technical accuracy verification. **a** Composition of a mock community consisting of 8 different bacterial species with a known composition was assessed (*n* = 8 per batch). The mock community used contained *Escherichia coli* and *Salmonella enterica*; which cannot be resolved using 16S rRNA gene sequencing [11]. We considered the relative abundance of unclassified *Enterobacteriaceae* as a surrogate for the cumulative relative abundance of these two species. **b** Taxonomic composition of biological controls was compared. Biological controls comprised of 9 samples originally extracted and sequenced in batch 1 that were re-sequenced in batch 2. Top 5 abundant ASVs per sample are visualised. **c** Prevalence and **d** relative abundance of all present ASVs (*N* = 2192) in biological controls were compared between batches. Each dot represents the average per batch. There is high agreement and consistency in the ASVs prevalence and relative abundance between the batches. Given the small sample size of biological controls, we did not identify potential reagent contaminants using the biological controls. The solid red line represents a perfect correlation. The dotted line shows the linear association between average relative abundance values of batches. ASV, amplicon sequencing variant; ICC, intraclass correlation coefficient

Technical reproducibility of library preparation and sequencing was confirmed on a mock community consisting of DNA extracted from 8 bacterial species (ZymoBIOMICS ™ Microbial Community Standard, Zymo Research, USA) and biological controls (comprising of 9 batch 1 samples re-sequenced in batch 2; Fig. 1a, b). The mock community used contained *Escherichia coli* and *Salmonella enterica*; two closely related *Enterobacteriaceae* species which cannot be resolved using 16S rRNA gene sequencing [11]. Although we did not identify these two taxa in the mock community, the relative abundance of unclassified *Enterobacteriaceae* was the expected cumulative relative abundance of the two enteric species (Fig. 1a). Substantial inter-individual variability was observed, as expected. However, the composition remained consistent between batches within each individual (Fig. 1b), and there was a high degree of agreement in the prevalence and relative abundances between batches (Fig. 1c, d).

### Two-tier strategy using the *decontam* algorithm and milk microbiota data structure to identify reagent contaminants

**Fig. 2:**
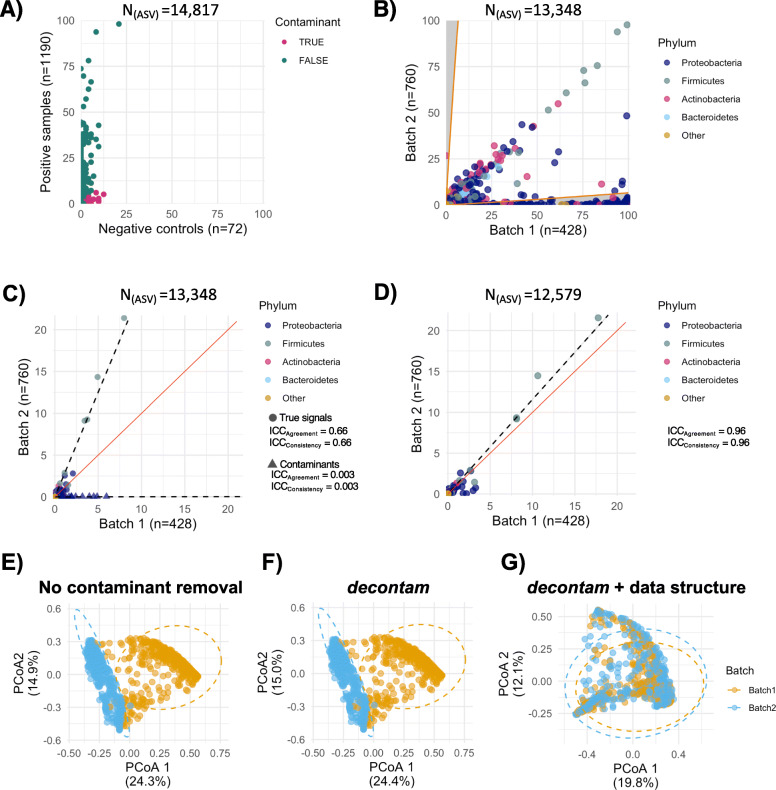
Two-tiered identification of potential contaminants and effects of their removal on batch variability. **a** Potential reagent contaminants were identified using the *decontam* package [13], which compared ASV prevalence between samples (*n* = 1190) and negative controls (*n* = 72). Two hundred fifty-six ASVs were identified as potential contaminant and removed. At this stage, unassigned ASVs, and ASVs belonging to the phylum Cyanobacteria, family of mitochondria and class of chloroplast (*n* = 780) were also removed. **b** Next, the data structure was used by between-batch comparison of ASV prevalence. We defined contaminants as any ASV with higher prevalence in one batch as would be expected in the other batch according to the standard error of prevalence calculated based on the batches’ sample size. The acceptable threshold is represented by the orange lines. Six hundred sixty ASVs below the orange lines were identified as potential contaminants. Quality control assessment of the data structure method was done on the between-batch comparison of ASVs (**c**) average relative abundances prior to and (**d**) after the removal of all potential contaminants. Relative abundances were re-calculated after the removal of the identified contaminants in panel **d**. There is high agreement and consistency in the relative abundance of “true” signals but not the contaminants. Removal of contaminants improved the between batch agreement and consistency of the remaining non-contaminant taxa. The solid red line represents a perfect correlation. The dotted line shows the linear association between average relative abundance values of batches. In panels **b**–**d**, each dot represents the average per batch. Next, batch variability was assessed **e** prior to contaminant removal, **f** after *decontam*, and again **g** after considering the data structure, i.e. taxa prevalence between the batches. The two-tier strategy eliminated the prominent separation of the samples on the PCoA plot assessed on Bray-Curtis dissimilarity. ASV, amplicon sequencing variant; ICC, intraclass correlation coefficient

As milk is a low biomass sample, reagent contaminants could plausibly be present in the sequencing output of samples [12]. As we have defined batches based on using reagents with different lot numbers, it is plausible that the major differences between the batches could be driven by different profiles of the reagent contaminants. A two-tier strategy was used to identify potential reagent contaminants followed by assessing the milk microbiota variability between batches prior to rarefaction and threshold filtering. First, potential reagent contaminants (*N* = 256 amplicon sequencing variants [ASVs]) were identified and removed using the *decontam* package based on either the frequency of the ASV in negative controls or the negative correlation with DNA concentration [13] (Fig. 2a). The negative controls included were extraction negative controls for batch 2 (*N* = 21) and no template PCR controls for batch 1 (*N* = 15) and batch 2 (*N* = 36). The extraction negative controls were not available for batch 1 and thus it was anticipated that some potential reagent contaminants might have remained after *decontam* especially in batch 1. Therefore, we next identified potential contaminants by comparing the data structure between batches. de Goffau et al. suggest that for low biomass samples such as milk for which we expect a “true” bacterial community to be present; “within-batch consistency of the reagent contamination profile and between batch variation of such profiles are two of the most powerful tools that can be used to recognize reagent contamination” [14]. Although this argument does not directly extend to non-contaminant “true” signals between batches, we postulate that unless a dramatic shift in microbiome composition is expected between the batches, the compositions will remain consistent between the batches and hence overall, the “true” signals will demonstrate high degrees of correlation between the batches.

We took advantage of the data structure of each batch to identify additional reagent contaminants that were not identified by the *decontam* algorithm. There was a high degree of correlation in the prevalence of ASVs in samples between the two batches (Fig. 2b). However, there were taxa more prevalent in one of the batches, specifically in batch 1 compared to batch 2 (Fig. 2b). We defined potential reagent contaminants based on comparing the prevalence of taxa in one batch to their corresponding prevalence in the other batch while accounting for the standard errors of the prevalence in order to adjust for the sample sizes. Although we have applied this approach to the biological controls (Fig. 1c), given the small sample size (*N* = 9), we did not consider them for identification of the potential contaminants. Next, we compared different sequencing runs within each batch and observed an overall high degree of agreement and consistency between the runs. Comparing between-run variabilities, we identified 198 and 66 ASVs as potential contaminants in batches 1 and 2, respectively (Figure S1A–D). Subsequently, by comparing the batches, 623 and 37 ASVs were identified as potential contaminants of batches 1 and 2, respectively (Fig. 2b). Of these, 144 and 9 were also identified in between-run comparisons of batches 1 and 2, respectively (Figure S1). In total, 769 ASVs were identified as contaminant through between-run (Figure S1) and/or between-batch (Fig. 2b) analysis. Overall, there was neither agreement nor consistency in the relative abundances of the contaminants between the batches (Fig. 2c). These additionally identified contaminant ASVs through between-run and/or between-batch comparisons were also removed, resulting in high agreement and consistency of the remaining, non-contaminant taxa between the batches (Fig. 2d). Comparison of the performance of contaminant identification using *decontam* and the data structure is summarised in Table S2. The agreement in relative abundances of the remaining non-contaminant taxa was 0.66 which was increased to 0.96 following identification of additional contaminants using the data structure (Table S2).

### Identification of contaminants using the data structure is influenced by the batch sample size but not the distribution of host characteristics

Next, we assessed the performance of the prevalence-based approach to identify contaminants in a homogenous subset of the data with varying batch sample sizes. We confirmed that between-batch variability in mother and infant characteristics did not impact the identified potential contaminants by the between-batch comparison using a subset consisting of primiparous, directly breastfeeding mothers and the child was not diagnosed with asthma at 5 years (Figure S2A). Although the sample size was lower than the entire dataset (*n* = 171 vs. 1188), 323 ASVs identified as contaminants in the homogenous subset accounted for 99% of the total reads of contaminant ASVs identified in the entire dataset (Table S2). Furthermore, we assessed the influence of batch sample size on contaminant identification. While this method increases the accuracy for large datasets, the definition of a contaminant becomes more relaxed in small datasets as larger variation in prevalence between the batches is expected. This is especially true for low prevalent taxa. We compared the performance of this method in smaller subsets of our data and observed that the 147 identified contaminants with 25 samples per batch accounted for 84% of total reads of contaminant ASVs identified in the entire dataset (Figure S2B). Similarly, 85% and 90% of total contaminant reads were accounted for in uneven datasets (more samples in batch 1 vs. more in batch 2; Figure S2C and D). This suggests low and uneven sample size is potentially important when using data structure for identifying potential contaminants.

We confirmed that in contrast to “true” taxa, the identified contaminants were highly correlated within batch 1 (Figure S3A) as stipulated and in agreement with de Goffau et al. [14]. All contaminant ASVs in batch 2 were low in abundance (< 0.1% mean relative abundance) and thus the correlation was not assessed for them. We did not observe strong correlation of non-contaminant taxa within each batch (Figure S3B and C).

### Reagent contaminants as the major source of batch variability

Despite the technical reproducibility, which was confirmed on mock community and biological controls (Fig. 1), preliminary comparisons between batches revealed differences in beta diversity of milk microbiota composition (Fig. 2e). This difference remained after applying *decontam*, which identified reagent contaminants in both batches (Fig. 2f). However, removing the additional potential contaminants identified through comparison of the data structure between batches improved the consistency in relative abundances of taxa between batches (Fig. 2d) and eliminated the differences in milk microbiota composition between the batches (Fig. 2g and Table S2).

### Repeatability and reproducibility assessment

**Fig. 3:**
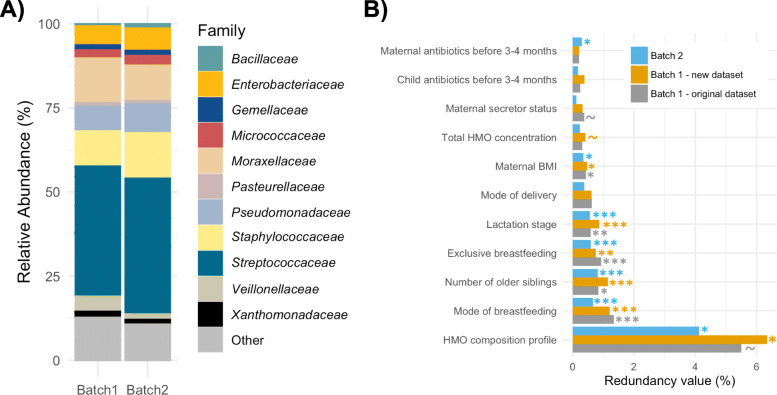
Repeatability and reproducibility of the results were assessed. **a** Comparison of milk microbiota taxonomy between the two batches. **b** Comparison of the statistical associations of determinants of the milk microbiota composition using redundancy analysis as previously described [10]. ***p < 0.001, **p < 0.01, *p < 0.05, ~*p* < 0.1

Next, we assessed the repeatability and reproducibility of the milk microbiota composition taxonomy and statistical associations with its determinants. The composition of the core ASVs (especially those suggested to be common reagent contaminants: *Comamonadaceae*, *Rhodospirillaceae* and Burkholderiales) was affected by the updated pre-processing and contaminant removal. As a result, the core microbiota as previously defined (ASVs present in at least 95% of samples with at least 1% mean relative abundance) [10] was not repeatable or reproducible, underscoring the challenges of defining core taxa in low biomass samples. However, the taxonomic structures of the most abundant taxa were consistent between the two batches (Fig. 3a).

Next, the robustness of the associations with determinants of the milk microbiota composition was assessed. We had previously performed redundancy analysis (RDA) on batch 1 to identify factors associated with the overall composition of milk microbiota [10]. We repeated the same RDA analysis on the re-processed batch 1 and the new batch 2 milk microbiota composition (Fig. 3b). We confirmed repeatability of the results within batch 1, despite removing several ASVs during the updated pre-processing. Additionally, most of the associations including mode of breastfeeding were reproduced in batch 2 (Fig. 3b). Based on these results, we felt confident to merge the two datasets for our ongoing research.

## Discussion

Rigorous quality control and assessment of repeatability and reproducibility of results are infrequently reported for microbiome studies. Here, we have proposed a framework for an in-depth step-by-step approach to a comprehensive quality control assessment of low biomass microbiome. The framework consists of three independent stages: (1) verification of sequencing accuracy by assessing technical repeatability and reproducibility of the results using mock communities and biological controls; (2) contaminant removal and batch variability correction by applying a two-tier strategy using statistical algorithms (e.g. *decontam*) followed by comparison of the data structure between (sub)batches; and (3) corroborating the repeatability and reproducibility of microbiome composition and downstream statistical analysis.

Batch variability can be minimised by adhering to standardised protocols and using consumables of the same lot; however, the latter may be impractical in longitudinal studies or when samples are analysed over a prolonged period of time, as in our study. In such circumstance, it is important to include sufficient numbers of biological controls (replicates) for repeat DNA extraction and sequencing, allowing for unbiased investigation of between-batch variability and potential reagent contaminants. Methods to minimise batch variability post-analysis have been developed based on various normalisation approaches, which generally assume that the batch variability is due to random technical variations [15, 16]. However, these global normalisation approaches (e.g. quintile normalisation) cannot eliminate the batch effect in variables that are differentially impacted in different batches [4]—for example, if the batch effect is due to differing reagent contaminant profiles instead of random technical variabilities.

Within the limitations of a sequencing-based study, identification of potential reagent contaminants in low biomass samples with credible microbial community is challenging. Using a combination of automated and data-driven contaminant identification increase our confidence that the remaining taxa are more likely composed of “true” signals. However, the performance of these methods relies on the researcher’s degree of stringency in choosing several parameters as well as the constraints of the dataset including the number of samples and negative controls. Moreover, given the low bacterial load of low biomass samples, reagent contaminants could potentially uniformly affect samples within a batch. Consequently, methods relying on the correlation of DNA concentration with taxa abundance are not capable of distinguishing whether high DNA concentration is due to high “true” bacterial load or high concentration of contaminants. Although taking advantage of the data structure between (sub)batches could to some extent overcome some of these challenges, it is crucial to emphasise that biological differences with expected strong influence on the microbiome composition should be carefully assessed. Additionally, it is conceivable that some biological factors could be causally linked to contamination of low biomass samples especially during sample collection. If such relations are hypothesised and demonstrated, it is important to have a priori scientific rationale for the definition of “true” signals. Finally, even with extended quality control approaches such as the one we have adopted, the identification of “true” signals requires confirmatory culture-dependent experiment.

Based on our previous and ongoing research, we did not expect any prominent associations of milk taxa with mother-infant characteristics. Consequently, we did not consider association of relative abundances of potential reagent contaminants within the milk microbiota with maternal, infant and early life factors as a criterion to retain them as “true” taxa. We stipulate that it is not easy to identify if an association between a potential contaminant and biological factor is real or is a product of their inherent correlation with DNA concentration or the compositionality issue of microbiome data in low biomass samples. However, it is important to highlight that under some circumstances, it might be necessary to take the association of relative abundances with biological parameters into account depending on the study design, hypotheses and ecosystem.

## Conclusion

Our study highlights the importance of reagent contaminants as a potential source of batch variability in low biomass samples [17] and provides a data-driven method to use the between-batch variability as a complementary approach to identify the potential contaminants. Guidelines have been developed to minimise the influence of contaminants in low biomass samples [18, 19]. However, these do not extend to contaminant-related batch variation. Our results indicate that conducting a comprehensive quality control assessment when profiling the microbiome of milk and other low biomass samples would ensure more robust, generalizable and reproducible results. Specifically, we recommend inclusion of appropriate negative controls and within-study quality control that takes advantage of the data structure (i.e. differential prevalence and/or abundance of contaminants between batches) to enhance the overall reliability and reproducibility of research in this field [20].

## Methods

### Study design

We studied a subset of 1194 mother-infant dyads in the Canadian Healthy Infant Longitudinal Development (CHILD) birth cohort, designed to study the developmental origins of paediatric asthma and allergy [9]. Women with singleton pregnancies were enrolled between 2008 and 2012 and remained eligible if they delivered a healthy infant > 35 weeks gestation (*n* = 3455). Milk microbiota from a representative subset of 428 mothers was previously profiled (2016; batch 1) [10]. An additional set of 766 samples enriched in infant atopy and asthma was included in this study (2019; batch 2). Participants gave written informed consent in accordance with the Declaration of Helsinki. The protocol was approved by the Human Research Ethics Boards at McMaster University, the Hospital for Sick Children, and the Universities of Manitoba, Alberta and British Columbia.

### Sample collection and microbiota analysis

Each mother provided one sample of milk collected during a 24-h period at 4 months postpartum [mean (SD) 17 (5) weeks postpartum] [10]. Batch 2 samples were processed similar to batch 1 as previously described [10]. Briefly, genomic DNA was extracted from 1 ml breastmilk using Quick-DNA Fungal/Bacterial extraction kit following the manufacturer’s instructions (Zymo Research, USA). Extraction kits were purchased separately for batch 1 and batch 2. Samples were sequenced following amplification of the V4 hypervariable region of the 16S rRNA gene with modified F515/R806 primers [21, 22] on a MiSeq platform (Illumina, San Diego, CA, USA) in 2016 (batch 1) and 2019 (batch 2). Sterile DNA-free water was used as negative controls in the DNA extraction (only batch 2) and sequencing library preparation (batches 1 and 2). A mock community consisting of DNA extracted from of eight bacterial species with known theoretical relative abundances (ZymoBIOMICS ™ Microbial Community Standard, Zymo Research, USA) was included as positive control in sequencing library preparation. Genomic DNA of nine milk samples previously extracted and sequenced in batch 1 were also included in sequencing library preparation of batch 2 as biological controls.

### Sequencing processing

Overlapping paired-end reads were processed with DADA2 pipeline [23] using the open-source software QIIME 2 v.2018.6 (https://qiime2.org) [24]. Unique ASVs were assigned a taxonomy and aligned to the 2013 release of the Greengenes reference database at 99% sequence similarity [25]. Demultiplexed sequencing data was deposited into the Sequence Read Archive (SRA) of NCBI and can be accessed via accession numbers PRJNA481046 and PRJNA597997.

### Reagent contaminant identification using *decontam*

Data analysis was conducted in R (v. 3.5.2) using the *Phyloseq* package (v. 1.26.1) [26, 27]. Potential reagent contaminants were identified using *decontam* package (v. 1.2.1) based on either the frequency of the ASV in the negative control or the negative correlation with DNA concentration with threshold set at 0.5 [13]. We applied *decontam* to all samples from both batches including all available negative controls. We used isContaminant function as we do not expect all ASVs in milk to be contaminants unless proven otherwise. The identified contaminants were removed from the dataset. Subsequently, unassigned ASVs, and ASVs belonging to the phylum Cyanobacteria, family of mitochondria and class of chloroplast (*n* = 780) were removed.

### Potential contaminant identification using the data structure

Different reagents were used in the processing of batches 1 and 2 and thus, we expected different contaminant profiles between the batches. Therefore, we built upon a previously suggested method [14] and identified additional potential contaminants by comparing the prevalence of ASVs between sequencing runs within each batch as well as between batches. Our approach is based on the assumption that differential prevalence of ASVs between batches is not unexpected in low biomass samples such as milk. However, considerable difference in the prevalence of ASVs between batches is suggestive of the influence of the reagent contaminants. Therefore, we defined contaminants as any ASV with higher prevalence (*P*) in one batch as would be expected in the other batch according to the standard error of prevalence (SEP) calculated based on the batches’ sample size (*N*). The standard error of prevalence (SEP) of ASVs (which we considered as proportions) were calculated as

$$ \mathrm{SEP}=\sqrt{P\times \left(1-P\right)}/N $$

We calculated the standard errors of prevalence for each batch and then calculated the minimum acceptable threshold (*T*) by subtracting the cumulative sum of standard errors in batches 1 and 2 from the observed prevalence; multiplied by a constant stringency factor (*k*).

$$ T=\left(P-\left({\mathrm{SEP}}_{\mathrm{Batch}1}+{\mathrm{SEP}}_{\mathrm{Batch}2}\right)\right)\times k $$

The stringency factor is a number between 0 and 1 and for this study it was set to 0.067. This data-driven approach was applied to pairwise comparison of different sequencing runs within each batch and also to the batches. Shaded areas on the figures were defined according the above formula (Fig. 2; Figures S1 and S2). All ASVs in the shaded area were considered contaminants and were removed.

Agreement and consistency of taxa relative abundances before and after contaminant removal were assessed using interclass correlation by 2-way random and fixed single measurement models using *irr* package (v. 0.84.1) [28]. Next, we assessed the potential impact of the biological variability in mother-infant characteristics on the performance of the data structure comparison. We selected a homogenous subset of samples according to parity, mode of breastfeeding and child asthma at the age of 5 years (the latter is enriched in batch 2). Overall, 171 mother-infant pairs (*N* = 63 in batch 1, *N* = 108 in batch 2) were included from primiparous mothers, directly breastfeeding, and whose child did not have asthma at 5 years. Additionally, we assessed the impact of sample size on the method performance using the homogenous data subset. Finally, within-batch consistency of the contaminant and non-contaminant profiles was assessed by Spearman rank-sum correlations among ASVs with over 0.1% average relative abundance and was visualised as heatmaps.

### Data processing following contaminant identification and removal

Subsequently, samples were rarefied to the minimum of 8000 sequencing reads per sample resulting in 870 samples and 9309 remaining ASVs. ASVs with less than 60 reads across the entire dataset were also removed, resulting in 908 remaining ASVs. This threshold was selected to retain the majority of reads per sample. By removing ASVs with total sum of 60 reads across the samples, we removed 20 ± 13% of ASVs per sample while retaining 99 ± 3% of the total sequencing reads. The number of sequencing reads per sample was then relativised to a total sum of 8000 for downstream analyses.

### Data quality control assessment

Technical reproducibility was assessed by agreements in taxonomic structure of biological controls, mock community and milk microbiota between batches. The batch effect was assessed on the overall milk microbiota composition using Bray-Curtis dissimilarity and visualised in PCoA plots. Repeatability was verified by examining the associations of maternal, infant and early life factors with milk microbiota using redundancy analysis (RDA) in the original batch 1 [10], new re-processed batch 1 and batch 2.

## Supplementary Information

**Additional file 1: Table S1.** Characteristics of mother-infant dyads from the CHILD cohort included in this study (n=870). **Table S2.** Comparison of the performance of contaminant identification using decontam and the data structure. **Figure S1.** Within-batch contaminant identification. **Figure S2.** Quality control checks for between-batch contaminant identification. **Figure S3.** Within-batch Spearman rank correlation assessment of the potential contaminant and non-contaminants.

**Additional file 2: Table S2. **List of identified contaminant taxa.

**Additional file 3. **Data analysis codes.

## Data Availability

The datasets generated and/or analysed during the current study are available in the Sequence Read Archive of NCBI repository (accession number PRJNA481046 and PRJNA597997). Requests for access to the metadata should be directed to the CHILD Cohort Study’s National Coordinating Centre (https://childstudy.ca/for-researchers/data-access). The codes used in this study are provided as the supplementary material.

## Abbreviations

ASV: Amplicon sequencing variant
CHILD: Canadian Healthy Infant Longitudinal Development
HMO: Human milk oligosaccharide
MQCP: Microbiome Quality Control Project
PCoA: Principal coordinate analysis
RDA: Redundancy analysis
SEP: Standard error of prevalence
