# Supplementary material for: Repeatability and reproducibility assessment in a large-scale population-based microbiota study: case study on human milk microbiota
Source: Microbiome. 2021 Feb 10;9:41. doi: 10.1186/s40168-020-00998-4 (PMC7877029; doi:10.1186/s40168-020-00998-4)

Supplemental information

Repeatability and reproducibility assessment in a large-scale population-based microbiota study: case study on human milk microbiota. Moossavi et al. Microbiome

Table S1. Characteristics of mother-infant dyads from the CHILD cohort included in this study (n=870).

Table S2. Comparison of the performance of contaminant identification using decontam and the data structure.

Figure S1. Within-batch contaminant identification.

Figure S2. Quality control checks for between-batch contaminant identification.

Figure S3. Within-batch Spearman rank correlation assessment of the potential contaminant and non-contaminants.

Table 1. Characteristics of mother-infant dyads from the CHILD cohort included in this study (n=870).

Factor		Mean ± SD or n (%) Initial subset Batch 1, N=337[a]	Mean ± SD or n (%) Additional subset Batch 2, N=533[a]	P value	Mean ± SD or n (%) All N=870
Maternal	Age (years)	33.1±4.1	32.8±4.4	0.284	32.9±4.3
	Pre-pregnancy BMI (Kg/m^2)	24.6±5.2	24.6±5.1	0.848	24.6±5.1
	History of atopy	190 (56.9)	315 (60.6)	0.318	505 (59.1)
	Asthma	72 (21.4)	135 (26.1)	0.138	207 (24.2)
	Secretor status	245 (73.6)	414 (77.7)	0.196	659 (76.1)
	Ethnicity			0.109	
	Caucasian	253 (75.1)	378 (72.6)		631 (73.5)
	Asian	57 (16.9)	80 (15.4)		137 (16.0)
	First Nations	12 (3.6)	17 (3.3)		29 (3.4)
	Other	15 (4.5)	46 (8.8)		61 (7.1)
	Ever smoking	75 (22.3)	140 (26.8)	0.153	215 (25.0)
Infant	Birth weight (g)	3,470±464	3,446±498	0.444	3,455+485
	Male sex	184 (54.6)	304 (57.0)	0.525	488 (56.1)
	Gestational age (weeks)	39.6±1.3	39.5±1.4	0.414	39.5±1.3
	Secretor status	250 (79.9)	353 (77.2)	0.435	603 (78.3)
	Atopy at 1	45 (13.5)	106 (21.1)	**0.007**	151 (18.1)
	Atopy at 3	56 (17.0)	102 (21.0)	0.188	158 (19.4)
	Asthma at 3			**<0.001**	
	Possible	30 (8.9)	74 (15.2)		104 (12.6)
	Probable	20 (5.9)	59 (12.1)		79 (9.6)
	Asthma at 5			**0.001**	
	Possible	29 (8.9)	58 (12.3)		87 (10.9)
	Definite	20 (6.1)	61 (13.0)		81 (10.2)
Early life	Mode of delivery			0.238	
	Vaginal	252 (75.9)	368 (71.5)		620 (73.2)
	Elective C/S	40 (12.0)	64 (12.4)		104 (12.3)
	Emergency C/S	40 (12.0)	83 (16.1)		123 (14.5)
	Maternal intrapartum antibiotics	119 (35.7)	227 (44.1)	**0.019**	346 (40.8)
	Maternal postpartum antibiotics before 3-4 months	32 (9.6)	63 (12.2)	0.291	95 (11.2)
	Child antibiotics before 3-4 months	7 (2.1)	11 (2.1)	1.000	18 (2.1)
	Multiparity	159 (47.2)	215 (41.2)	0.097	374 (43.5)
BF & HMOs	Direct BF (at the breast) only	139 (41.2)	187 (35.8)	0.240	326 (38.0)
	Duration of BF (months)	12.9±5.9	12.3±6.2	0.154	12.5±6.1
	Duration of exclusive BF (months)	3.4±2.3	3.3±2.2	0.357	3.3±2.3
	HMO concentration (mg/mL)	10.3±2.1	10.4±2.1	0.755	10.4±2.1
	HMO Simpson's diversity	4.9±1.4	4.9±1.4	0.489	4.9±1.4
Environment	Study city			0.248	
	Edmonton	81 (24.0)	98 (18.8)		179 (20.8)
	Toronto	92 (27.3)	142 (27.2)		234 (27.2)
	Vancouver	81 (24.0)	132 (25.3)		213 (24.8)
	Winnipeg	83 (24.6)	150 (28.7)		233 (27.1)
	Milk collection in spring [b]	59 (17.6)	168 (32.3)	**<0.001**	227 (26.5)
	Birth season			**0.045**	
	Spring	98 (29.1)	140 (26.8)		238 (27.7)
	Summer	101 (30.0)	123 (23.6)		224 (26.1)
	Autumn	75 (22.3)	123 (23.6)		201 (23.4)
	Winter	63 (18.7)	133 (25.5)		196 (22.8)

[a] N after pre-processing of microbiome data, contaminant removal, and rarefaction; [b] versus other seasons combined. BF, breastfeeding; C/S: Caesarian section; HMO, human milk oligosaccharide

Table 2. Comparison of the performance of contaminant identification using *decontam* and the data structure. Also see Figure S1 and Figure 2.

Parameters	*decontam*	Data structure				
		Between-run contaminants[1]		Between-batch contaminants[1]		All unique contaminants identified
		Batch 1	Batch 2	Batch 1	Batch 2	
Number of ASVs identified as contaminant	256	198	66[2]	623	37	769
Average prevalence of a contaminant ASV (%)	0.56	0.6 (0.9)	2.9 (14.3)	5.6 (14.9)	1.3 (6.7)	4.8 (15.1)
Batch 1, Run 1 (n=215)	0.99	2.2 (1.2)	6.3 (19.9)	15.4 (27.3)	0	12.7 (25.2)
Batch 1, Run 2 (n=213)	0.68	0.4 (0.9)	6.4 (20.2)	15.4 (28.5)	0	12.6 (26.3)
Batch 2, Run 1 (n=252)	0.45	0.1 (0.5)	0.9 (1.0)	0.1 (0.5)	2.4 (6.1)	0.3 (1.5)
Batch 2, Run 2 (n=253)	0.42	0.1 (0.4)	1.1 (1.0)	0.1 (0.5)	2.0 (4.3)	0.3 (1.2)
Batch 2, Run 3 (n=255)	0.37	0.1 (0.4)	0.7 (0.8)	0.1 (0.5)	1.7 (2.8)	0.3 (0.9)
Average percent of reads per sample identified as contaminants (%)	0.73	0.2 (1.4)	1.3 (2.9)	18.6 (29.8)	0.2 (1.4)	19.2 (29.7)
Batch 1, Run 1 (n=215)	0.62	0.4 (1.7)	2.7 (1.7)	51.0 (28.9)	0	51.2 (28.8)
Batch 1, Run 2 (n=213)	0.13	0.2 (0.9)	3.1 (1.6)	51.6 (27.4)	0	51.9 (27.3)
Batch 2, Run 1 (n=252)	1.08	0.2 (1.2)	0.2 (1.2)	0.1 (0.5)	0.5 (1.7)	1.0 (2.6)
Batch 2, Run 2 (n=253)	1.16	0.2 (1.9)	0.5 (4.1)	0.2 (0.8)	0.3 (1.7)	1.0 (4.6)
Batch 2, Run 3 (n=255)	0.53	0.1 (0.9)	0.4 (3.0)	0.3 (1.2)	0.3 (1.7)	1.1 (3.9)
Percent of total contaminant reads (%)	18.2 (31.2)	6.1 (19.3)	13.0 (24.0)	51.8 (45.0)	14.6 (28.2)	81.8 (31.2)
Agreement of remaining ASVs [3]	ICC_A= 0.58	ICC_A= 0.96				
Batch effect on the overall composition following removal of contaminants[4]	R^2=17.9% (p=0.001)	R^2=1.4% (p=0.001)				

[1]There is some overlap in the contaminants identified within the various between-run and between-batch comparisons, Figure S4 shows the overlap in ASVs were identified as contaminants among comparisons. [2]5 ASVs identified as between-run Batch 2 contaminants were also identified as Batch 1 contaminants by Between-batch comparisons and accounted for an average of 2.6% and 3.0% of reads per sample in Run 1 and Run 2 of Batch 1, respectively. [3]The agreement in ASV average relative abundances between batches using intraclass correlation (ICC) after removal of contaminant ASVs. [4]Using ADONIS from the *vegan* package on Bray-Curtis dissimilarity (see Figure 2E-2G for PCoA plots). Started at R^2=17.9% (p=0.001) prior to any contaminant removal.

Figure S1. Within-batch contaminant identification. ASV prevalence was compared between A) Run 1 and Run 2 within Batch 1 (N=198 identified as contaminant), B) Run 1 and Run 2 within Batch 2 (N=33 identified as contaminant), C) Run 1 and Run 3 within Batch 2 (N=21 identified as contaminant), and D) Run 2 and Run 3 within Batch 2 (N=32 identified as contaminant). A total of 66 unique ASVs were identified as within-Batch 2 contaminants (There was some overlap in contaminant ASVs identified between different runs of Batch 2). We defined contaminants as any ASV with higher prevalence in one run as would be expected in the other run according to the standard error of prevalence calculated based on the sample size. The acceptable threshold is represented by the orange lines. ASVs below the orange lines were identified as potential contaminants. All the identified contaminants have very low prevalence (2% on average) and thus are concentrated in the lower left corner of the figures. Between-run identification of contaminants were performed after applying decontam but prior to between batch comparison (see **Figure 2**). ASV, amplicon sequencing variant; ICC, intraclass correlation coefficient.

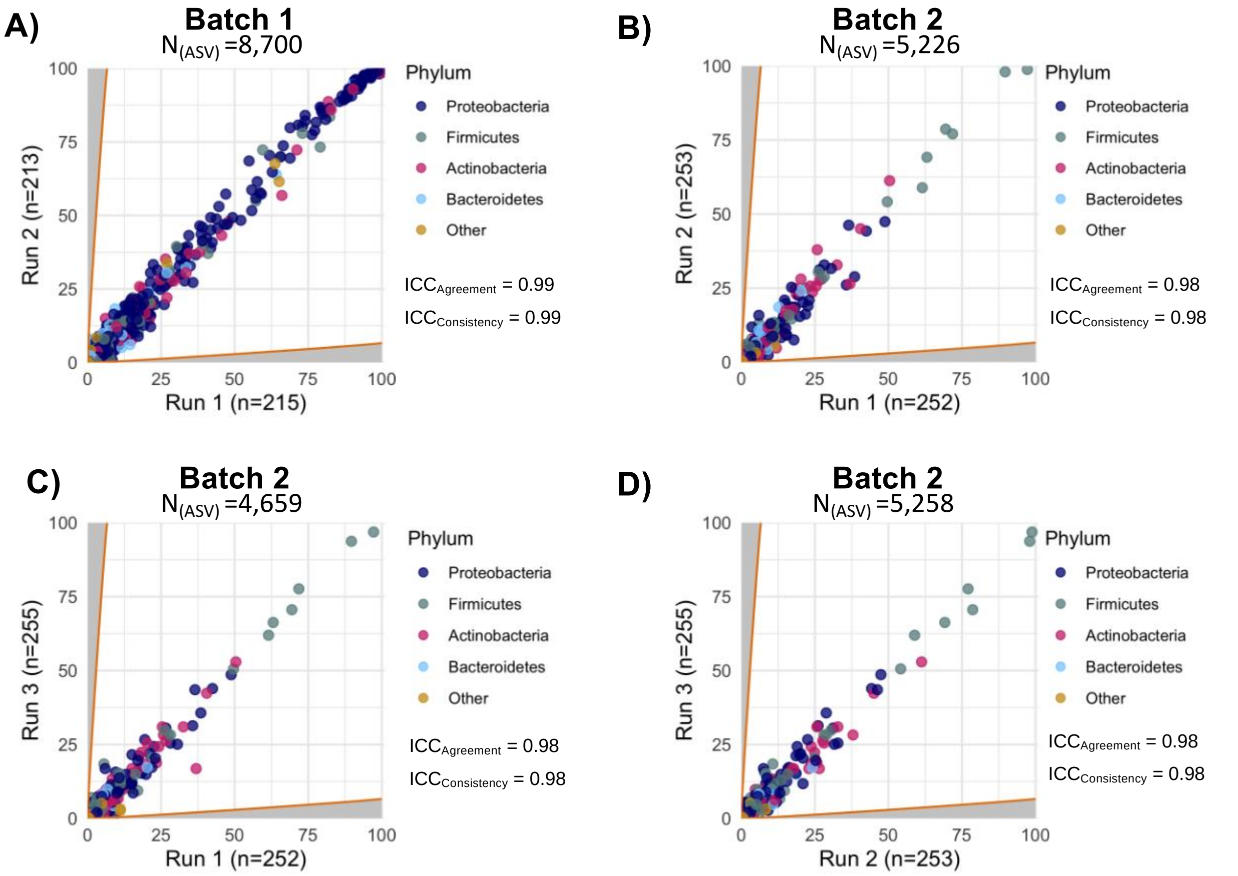

Figure S2. Quality control checks for between-batch contaminant identification. A) Comparing ASV prevalence between batches for a homogenous subset from mothers that 1) were primiparous, 2) directly breastfed, 3) who's child did not have asthma at 5 years. B-D) Comparisons of ASV prevalence between batches with different sample sizes. Samples were selected from the homogenous subset of Figure S2A. We defined contaminants as any ASV with higher prevalence in one run as would be expected in the other run according to the standard error of prevalence calculated based on the sample size. The acceptable threshold is represented by the orange lines. ASVs below the orange lines were identified as potential contaminants. Between-run identification of contaminants were performed after applying decontam but prior to between batch comparison (see Figure 2). ASV, amplicon sequencing variant; ICC, intraclass correlation coefficient.

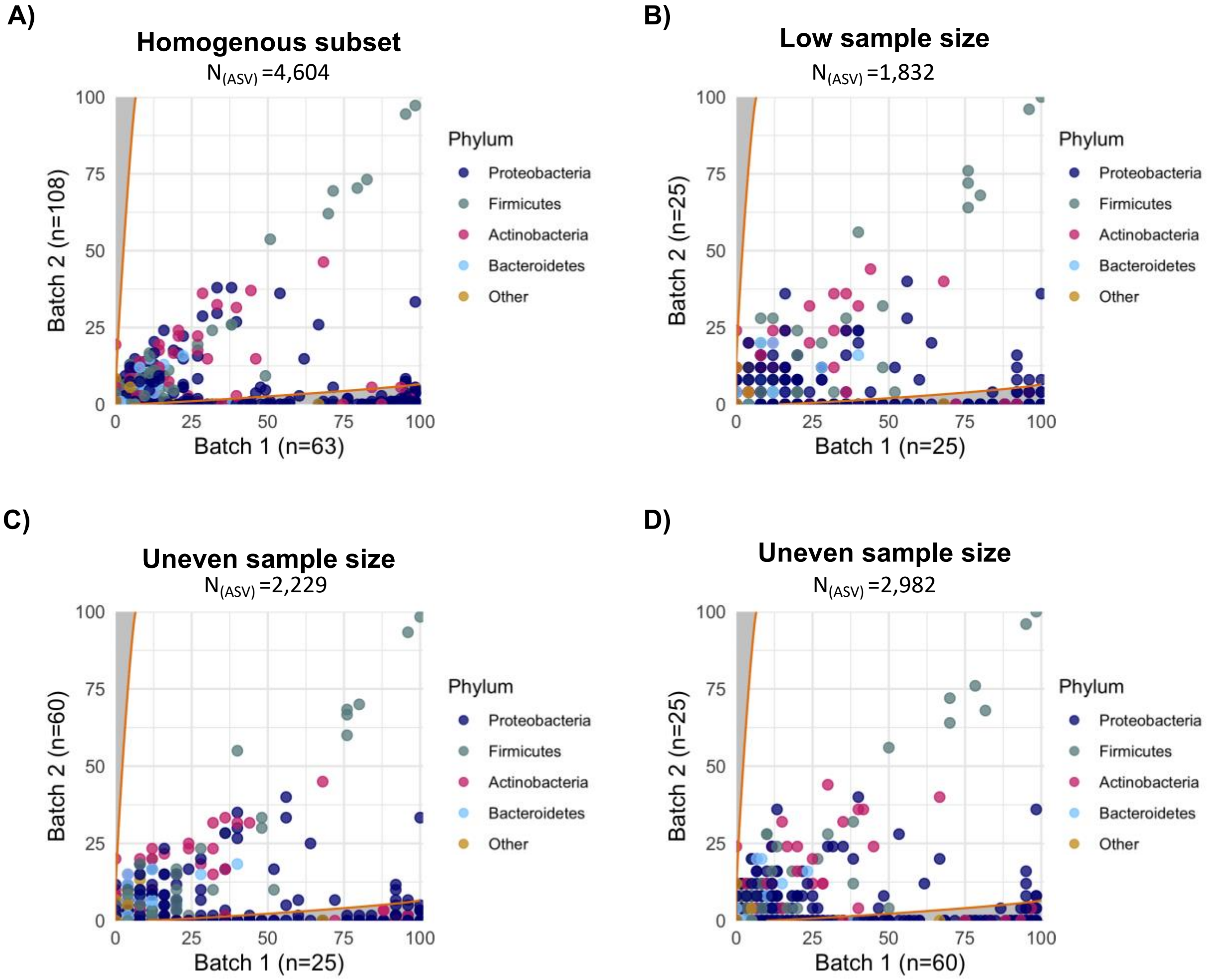

Figure S3. Within-batch Spearman rank correlation assessment of the potential contaminant and non-contaminants. A) Correlation of contaminant ASVs (n=51) and B) non-contaminant ASVs (n=58) within Batch 1. C) Correlation of non-contaminant ASVs (n=76) within Batch 2. We defined contaminants as any ASV with higher prevalence in one run as would be expected in the other run according to the standard error of prevalence calculated based on the sample size. ASVs with > 0.1% mean relative abundance are included (see Figure 2). Potential contaminants in Batch 2 had less than 0.1% mean relative abundance; and thus within-batch correlation of Batch 2 contaminants is not assessed.

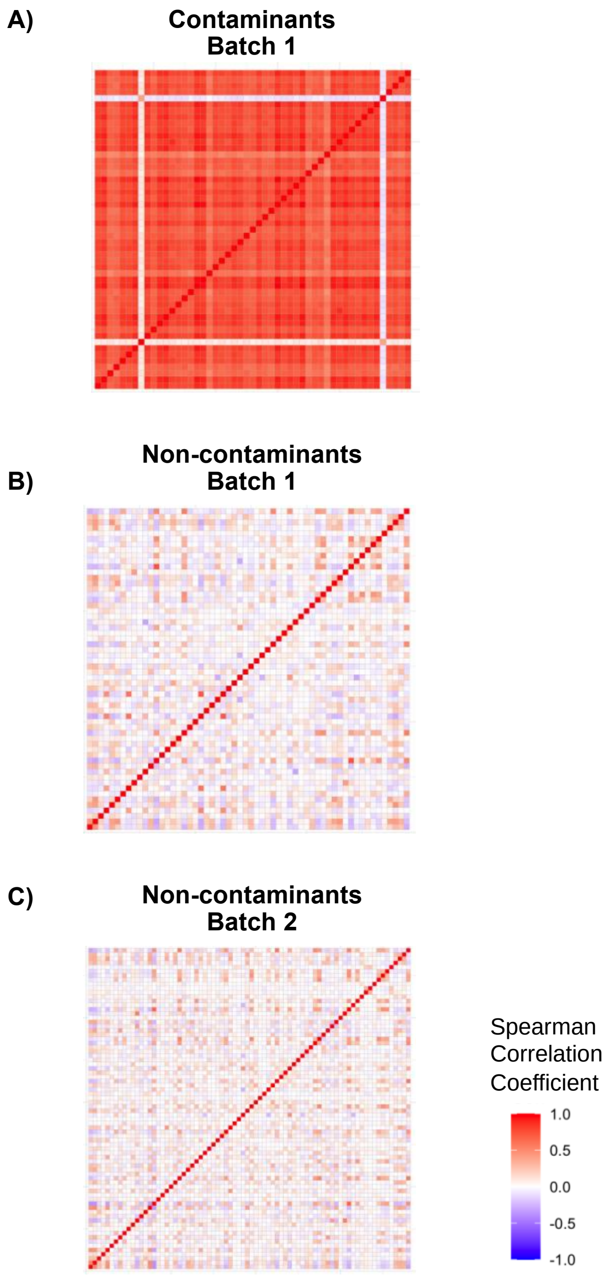

Supplement: Supplementary file 2 — Additional file 1: Table S1. Characteristics of mother-infant dyads from the CHILD cohort included in this study (n=870). Table S2. Comparison of the performance of contaminant identification using decontam and the data structure. Figure S1. Within-batch contaminant identification. Figure S2. Quality control checks for between-batch contaminant identification. Figure S3. Within-batch Spearman rank correlation assessment of the potential contaminant and non-contaminants. [file 40168_2020_998_MOESM2_ESM.pdf]
