# Supplementary material for: Repeatability and reproducibility assessment in a large-scale population-based microbiota study: case study on human milk microbiota
Source: Microbiome. 2021 Feb 10;9:41. doi: 10.1186/s40168-020-00998-4 (PMC7877029; doi:10.1186/s40168-020-00998-4)
Supplement: Supplementary file 4 — Additional file 3. Data analysis codes. [file 40168_2020_998_MOESM4_ESM.html]

Repeatability and reproducibility assessment in a large-scale population-based microbiota study: case study on human milk microbiota


# Repeatability and reproducibility assessment in a large-scale population-based microbiota study: case study on human milk microbiota

#### Kelsey Fehr & Shirin Moossavi

#### 02/05/2021

```
library(phyloseq) 
library(ggplot2)
library(tidyverse)
library(reshape2) 
library(decontam) 
library(RColorBrewer) 
library(vegan) 
library(zCompositions)
library(CoDaSeq)
library(irr)
library(plyr)
```

```
#Function to extract phyloseq OTU Table  
veganotu = function(data) {
    require("vegan")
    OTU = otu_table(data)
    if (taxa_are_rows(OTU)) {
        OTU = t(OTU)
    }
    return(as(OTU, "matrix"))
}
```

```
# Summary function
data_summary <- function(data, varname, groupnames){
  require(plyr)
  summary_func <- function(x, col){
    c(mean = mean(x[[col]], na.rm=TRUE),
      sd = sd(x[[col]], na.rm=TRUE), "median" = median(x[[col]], na.rm = TRUE), min = min(x[[col]], na.rm=TRUE), max = max(x[[col]], na.rm=TRUE), IQR = IQR(x[[col]], na.rm = TRUE, type = 7), Q1 = quantile(x[[col]], 0.25, type = 7, na.rm=TRUE), Q3 = quantile(x[[col]], 0.75, type = 7, na.rm=TRUE), 
      sem = sd((x[[col]])/{sqrt(NROW(x))}, na.rm=TRUE), "N not 0" = sum(x[[col]] != 0, na.rm=TRUE), "% N not 0" = (sum(x[[col]] != 0)/NROW(x))*100)
  }
  data_sum<-ddply(data, groupnames, .fun=summary_func,
                  varname)
  data_sum <- rename(data_sum, c("mean" = varname))
 return(data_sum)
}
```

Load the phyloseq object

```
load(file='input_data/phyloseq')
physeq
```

```
## phyloseq-class experiment-level object
## otu_table()   OTU Table:         [ 15954 taxa and 1316 samples ]
## sample_data() Sample Data:       [ 1316 samples by 14 sample variables ]
## tax_table()   Taxonomy Table:    [ 15954 taxa by 7 taxonomic ranks ]
```

# Dictionary of the main Phyloseq objects and steps throughout code:

1. physeq: Object that contains the original ASV table and taxonomy table that were output of the QIIME2 pipeline (using the DADA2 denoising algorithm).
2. ps.noncontam: Object made by removing contaminating ASVs identified by the decontam R package from "physeq".
3. physeq\_sample2: Object made by removing controls (negative and positive) and removing reads unassigned at the Kingdom level, and reads assigned to mitochondria, Chloroplast and Cyanobacteria taxonomy from "ps.noncontam".
4. data\_prune: Object made by removing additional potential contaminating ASVs identified by assessing the data strucutre (comparing runs within batches and comparing Batch 1 and Batch 2 milk samples) from "physeq\_sample2".
5. data\_rarefy\_pruned: Object made by rarefying "data\_prune" to a depth of 8,000 reads per sample
6. data\_prune3: Object made by removing rare taxa (ASVs present with a total count less than 60 reads across the dataset) from "data\_rarefy\_pruned".
7. data\_rarefy\_rel: Object with samples in data\_prune3 relativized to the total reads per sample after rarfaction (8,000). The final dataset used for downstream analyses and Figure 2G, 3A & 3B.
8. data\_rarefy\_rel\_ext: Object used for Figure 2E, made from "physeq" (original object before preprocessing). Went through the same preprocessing as "data\_rarefy\_rel" with the exclusion of step 2 (decontam) and step 4 (removal of additional potential contaminants) above.
9. data\_rarefy\_rel\_ext2: Object used for Figure 2F, made from physeq\_sample2 (object that went through decontam and other preprocessing). Went through the same preprocessing as "data\_rarefy\_rel" with the exclusion of step 4 above (removal of additional potential contaminants).

# The Function made to identify potential contaminants by assessing the data structure (See Step 4 in the Dictionary of the main Phyloseq objects and steps)

```
# FORMULA for the minumum accepted prevalence in one batch given an ASVs prevalence in the other batch, using the standard errors (SE) of the ASV in both batches (assuming it has the same prevalence in both batches).

# NOTE: this isn't a formal statistical test. This is just a practical framework based on the idea that if an ASVs proportion +/- SE in one Batch crossess over with the proportion +/- SE in the other batch, then the proportions do not differ.

Structure_comp <- function(x)  {(x - sqrt(x*(1-x)/N1) - sqrt(x*(1-x)/N2))*k}
# let x = the expected ('reference') prevalence/proportion of an ASV. If we think the prevalence of the ASV is this, given the sample sizes of both batches (N1 and N2), what is the lowest proportion "expected" in the other batch?

# let k = a constant stringency factor provided by the user (value between 0 and 1, the lower the value, the more relaxed the test is) How similar is the prevalence expected to be between batches?
k = 1/15  # If set to 1, expect to be exactly the same, very strict test
## we have relaxed the threhold by a constant of 0.067. We do not expect the prevalence of an ASV to be exactly the same between batches.

# Output: Given a list of expected proportions (x) of each ASV in (for example) Batch 1, the output is a list of the lowest expected proportion of each ASV in Batch 2
```

# Verification of sequencing technical accuracy on mock community and biological controls

## FIGURE 1A: Mock community

## Preparation of the data

1. Creating a dataframe of the known theoretical composition

```
# Copying theoretical relative abundances exactly from Table 1 of the mock communities instructional manual: https://files.zymoresearch.com/protocols/_d6305_d6306_zymobiomics_microbial_community_dna_standard.pdf
Expected_com <- c("4.2", "10.1", "10.4", "18.4", "9.9", "15.5", "14.1", "17.4")
Expected_com <- as.data.frame(Expected_com)

# Adding the genus classifiecations in the same order. 
## Genus of the species Escherichia coli & Salmonella enterica are both grouped as "Unclassified Enterobacteriaceae" since these cannot be distinguished using 16S sequencing.
Expected_com$Genus <- c("Pseudomonas", "Unclassified Enterobacteriaceae", "Unclassified Enterobacteriaceae", "Lactobacillus", "Enterococcus", "Staphylococcus", "Listeria", "Bacillus")
Expected_com$Expected_com <- as.numeric(as.character(Expected_com$Expected_com))

sum(Expected_com$Expected_com) #sums to 100, are percentages out of the total of these 7 Genera.
```

2. Mock Community composition after sequencing

```
Mocks <- subset_samples(physeq, Smple_type == "Mock_sequencing")
Mocks <- prune_taxa(taxa_sums(Mocks)>0, Mocks)
Mocks
total_mock_reads <- sample_sums(Mocks)

# Only retain taxa that is expected to be present in mock communities:
Mocks2 <- subset_taxa(Mocks, Genus == "g__Pseudomonas" | Genus == "g__Lactobacillus" | Genus == "g__Enterococcus" | Genus == "g__Staphylococcus" | Genus == "g__Listeria" | Genus == "g__Bacillus" | Genus  == "g__Unclass.Enterobacteriaceae")
Mocks2
get_taxa_unique(Mocks2, taxonomic.rank = "Genus") 

#Assess the combined relative abundance of the true mock community taxa
mock_reads_7genera <- sample_sums(Mocks2)

relative_reads_mockGenera <- mock_reads_7genera/total_mock_reads
summary(relative_reads_mockGenera) 
# The true mock community taxa make up the majority of original reads in the sequenced mock community. <1% of the remaining reads were found to be reagent contaminants. 

#Relativise the read counts to the  total sum per mock community sample
Seq_Mock <- transform_sample_counts(Mocks2, function(x) {x*100/sum(x)})
```

3. FIGURE 1A: Stacked bar chart comparing mock community compositions between batches

```
#MELT datasets
m <- psmelt(Seq_Mock)

#remove the prefixes from the taxonomy file
m$Phylum <- gsub("^p__", "", m$Phylum)
m$Family <- gsub("^f__", "", m$Family)
m$Genus <- gsub("^g__", "", m$Genus)

# Replace NA (g__) in Genus with FAMILY ID IN THE NEXT COLUMN for the plot and make unclassified IDs consistently named
m$Genus <- gsub("Unclass\\.Enterobacteriaceae", "Unclassified Enterobacteriaceae", m$Genus)

#Make a column with taxonomy that is as unique as OTUs for DATA Summary 
m$OTU_Phylum_Family_Genus <- paste(m$Phylum, m$Family, m$Genus, m$OTU, sep = ":") # just concatinating because want to keep ASV taxonomy in the summarized data but do not want to summarize based on taxonomy (just makae same color in plot).

m_summ <- data_summary(m, varname = "Abundance", groupnames = c("Batch", "OTU_Phylum_Family_Genus"))
m_summ <- m_summ %>% separate(OTU_Phylum_Family_Genus, 
                c("Phylum", "Family", "Genus", "OTU"), sep = ":") # Abundance = average


#Use rbind.fill to Add Expected_com/Expected_Plot expected composition columns to table, just adding 'theoretical composition' to the 'Abundance' column
## First editing the table so that it can bind to m_summ
Expected_Plot <- Expected_com
Expected_Plot$Smple_type <- "Theoretical"
Expected_Plot$Batch <- "Theoretical"
Expected_Plot$Abundance <- Expected_Plot$Expected_com
Expected_Plot$Expected_com <- NULL

Mocks_Plot <- rbind.fill(m_summ, Expected_Plot)
Mocks_Plot$Batch <- ordered(Mocks_Plot$Batch, levels = c("Theoretical", "Batch1" , "Batch2"), labels =c("Known\ncomposition", "Batch 1", "Batch 2"))
Mocks_Plot$Genus <- revalue(Mocks_Plot$Genus, c("Unclassified Enterobacteriaceae"="Unclassified\nEnterobacteriaceae"))

# Make some labels italic? Can't figure it out for a single label "Unclassified Enterobacteriaceae"


Fig1A = ggplot(Mocks_Plot, aes(x=Batch, y=Abundance, fill=Genus))+
        geom_bar(stat="identity", position="stack") + 
        scale_y_continuous(expand=c(0,0)) + 
        scale_fill_manual(values = col_blind7) + 
        labs(y="Relative Abundance (%)") + 
        theme_minimal()+
        theme(axis.ticks.x = element_blank(), axis.title.x = element_blank(), axis.text.x = element_text(size=11, angle = 0, hjust = 0.5), legend.text = element_text(face = "italic"))+ 
        xlab("")
        
Fig1A
```

## FIGURE 1B: Biological controls

9 Samples which were originally extracted and sequenced in Batch 1 (Moossavi et al., Cell Host Microbe, 2019) were included in the Batch 2 sequencing.

1. Data preparaton: extracting the biological samples from the dataset

```
BCs <- subset_samples(physeq, Smple_type == "Biologic_Control" | SampleID == "103" | SampleID == "148" | SampleID == "186" | SampleID == "200" | SampleID == "226" | SampleID == "356" | SampleID == "55" | SampleID == "77" | SampleID == "83" )

BCs <- prune_taxa(taxa_sums(BCs) > 0, BCs) # To check actual number of taxa in biological controls
BCs
```

2. Extract the top 5 most abundant ASVs in each pair of replicate biological control samples, we aim to compare the most abundant taxa in the biological controls between batches.

```
#Relativize
BC_rel <- transform_sample_counts(BCs, function(x) {x*100/sum(x)}) 

#Sample 1
BC1_rel <- subset_samples(BC_rel, rownames(sample_data(BC_rel)) == "Z103.B1" |  rownames(sample_data(BC_rel)) == "X30139")
BC1_Top <- names(sort(taxa_sums(BC1_rel), TRUE)[1:5])

#Sample 2
BC2_rel <- subset_samples(BC_rel, rownames(sample_data(BC_rel)) == "Z148.B1" |  rownames(sample_data(BC_rel)) == "X20137")
BC2_Top <- names(sort(taxa_sums(BC2_rel), TRUE)[1:5])

#Sample 3
BC3_rel <- subset_samples(BC_rel, rownames(sample_data(BC_rel)) == "Z186.B1" |  rownames(sample_data(BC_rel)) == "X50139")
BC3_Top <- names(sort(taxa_sums(BC3_rel), TRUE)[1:5])

#Sample 4
BC4_rel <- subset_samples(BC_rel, rownames(sample_data(BC_rel)) == "Z200.B1" |  rownames(sample_data(BC_rel)) == "X30288")
BC4_Top <- names(sort(taxa_sums(BC4_rel), TRUE)[1:5])

#Sample 5
BC5_rel <- subset_samples(BC_rel, rownames(sample_data(BC_rel)) == "Z226.B1" |  rownames(sample_data(BC_rel)) == "X50184")
BC5_Top <- names(sort(taxa_sums(BC5_rel), TRUE)[1:5])

#Sample 6
BC6_rel <- subset_samples(BC_rel, rownames(sample_data(BC_rel)) == "Z356.B1" |  rownames(sample_data(BC_rel)) == "X20303")
BC6_Top <- names(sort(taxa_sums(BC6_rel), TRUE)[1:5])

#Sample 7
BC7_rel <- subset_samples(BC_rel, rownames(sample_data(BC_rel)) == "Z55.B1" |  rownames(sample_data(BC_rel)) == "X40303")
BC7_Top <- names(sort(taxa_sums(BC7_rel), TRUE)[1:5])

#Sample 8
BC8_rel <- subset_samples(BC_rel, rownames(sample_data(BC_rel)) == "Z77.B1" |  rownames(sample_data(BC_rel)) == "X20118")
BC8_Top <- names(sort(taxa_sums(BC8_rel), TRUE)[1:5])

#Sample 9
BC9_rel <- subset_samples(BC_rel, rownames(sample_data(BC_rel)) == "Z83.B1" |  rownames(sample_data(BC_rel)) == "X40135")
BC9_Top <- names(sort(taxa_sums(BC9_rel), TRUE)[1:5])

#merging all sample
B_top_all <- c(BC1_Top, BC2_Top, BC3_Top, BC4_Top, BC5_Top, BC6_Top, BC7_Top, BC8_Top, BC9_Top) 

#Removing duplicate taxa
dups <- duplicated(B_top_all) 
sum(dups[TRUE]) #11
B_top_all <- subset(B_top_all, !(dups)) # 34

BC_prune <- prune_taxa(B_top_all, BC_rel)
get_taxa_unique(BC_prune, taxonomic.rank = "Genus")
```

3. FIGURE 1B. Stacked bar chart comparing milk microbiota compositions in biological controls between batches

```
BC_rel_melt <- psmelt(BC_prune) 

# Change the sequencing IDs of the biological controls in Batch 2 so that the biological controls in Batch 2 have the same ID as its matched sample in Batch 1
BC_rel_melt$BC_CHILDid <- BC_rel_melt$Sample
BC_rel_melt$BC_CHILDid <- gsub("Z103.B1","X30139", BC_rel_melt$BC_CHILDid)
BC_rel_melt$BC_CHILDid <- gsub("Z148.B1","X20137", BC_rel_melt$BC_CHILDid)
BC_rel_melt$BC_CHILDid <- gsub("Z186.B1","X50139", BC_rel_melt$BC_CHILDid)
BC_rel_melt$BC_CHILDid <- gsub("Z200.B1","X30288", BC_rel_melt$BC_CHILDid)
BC_rel_melt$BC_CHILDid <- gsub("Z226.B1","X50184", BC_rel_melt$BC_CHILDid)
BC_rel_melt$BC_CHILDid <- gsub("Z356.B1","X20303", BC_rel_melt$BC_CHILDid)
BC_rel_melt$BC_CHILDid <- gsub("Z55.B1","X40303", BC_rel_melt$BC_CHILDid)
BC_rel_melt$BC_CHILDid <- gsub("Z77.B1","X20118", BC_rel_melt$BC_CHILDid)
BC_rel_melt$BC_CHILDid <- gsub("Z83.B1","X40135", BC_rel_melt$BC_CHILDid)


# Replace NA (g__) in Genus with FAMILY ID IN THE NEXT COLUMN for the plot and make unclassified IDs consistently named
levels(BC_rel_melt$Genus)
```

```
##  [1] "g__"                           "g__Acinetobacter"             
##  [3] "g__Aeromonas"                  "g__Azospirillum"              
##  [5] "g__Chryseobacterium"           "g__Corynebacterium"           
##  [7] "g__Enhydrobacter"              "g__Janthinobacterium"         
##  [9] "g__Kocuria"                    "g__Lactobacillus"             
## [11] "g__Lactococcus"                "g__Pseudomonas"               
## [13] "g__Ralstonia"                  "g__Rothia"                    
## [15] "g__Staphylococcus"             "g__Stenotrophomonas"          
## [17] "g__Streptococcus"              "g__Unclass.Burkholderiales"   
## [19] "g__Unclass.Comamonadaceae"     "g__Unclass.Enterobacteriaceae"
## [21] "g__Veillonella"
```

```
#1st need to replace NA/blank in Genus with FAMILY level taxonomy
BC_rel_melt$Genus <- as.character(BC_rel_melt$Genus)
BC_rel_melt$Genus <- ifelse(BC_rel_melt$Genus == "g__", 
           paste("Unclassified", BC_rel_melt$Family), BC_rel_melt$Genus)
BC_rel_melt$Genus <- gsub("g__", "", BC_rel_melt$Genus)
BC_rel_melt$Genus <- gsub("f__Unclass\\.", "", BC_rel_melt$Genus)
BC_rel_melt$Genus <- gsub("Unclass\\.", "Unclassified ", BC_rel_melt$Genus)

BC_rel_melt$Genus <- as.factor(BC_rel_melt$Genus)
levels(BC_rel_melt$Genus)
```

```
##  [1] "Acinetobacter"                   "Aeromonas"                      
##  [3] "Azospirillum"                    "Chryseobacterium"               
##  [5] "Corynebacterium"                 "Enhydrobacter"                  
##  [7] "Janthinobacterium"               "Kocuria"                        
##  [9] "Lactobacillus"                   "Lactococcus"                    
## [11] "Pseudomonas"                     "Ralstonia"                      
## [13] "Rothia"                          "Staphylococcus"                 
## [15] "Stenotrophomonas"                "Streptococcus"                  
## [17] "Unclassified Alteromonadales"    "Unclassified Burkholderiales"   
## [19] "Unclassified Comamonadaceae"     "Unclassified Enterobacteriaceae"
## [21] "Veillonella"
```

```
BC_rel_melt$Batch <- revalue(BC_rel_melt$Batch, c("Batch1"="Batch 1", "Batch2"="Batch 2"))
BC_rel_melt$BC_CHILDid <- revalue(BC_rel_melt$BC_CHILDid, c("X20118"="Sample 1", "X20137"="Sample 2", "X20303"="Sample 3", "X30139"="Sample 4", "X30288"="Sample 5", "X40135"="Sample 6", "X40303"="Sample 7", "X50139"="Sample 8", "X50184"="Sample 9"))


Fig1B = ggplot(BC_rel_melt, aes(x=Batch, y=Abundance, fill=Genus))+
           geom_bar(stat="identity", position="stack", alpha=1) +  
           facet_wrap(~BC_CHILDid) + 
           labs(y="Relative Abundance (%)") +
           scale_fill_manual(values = c(col_blind21), guide = guide_legend(ncol= 1)) +
           theme_minimal()+
           theme(legend.position = "right", axis.title.x= element_blank(), legend.text = element_text(face = "italic"))+
           xlab("")+
           ylim(0,100)

Fig1B
```

## FIGURE 1C: Comparison of ASV prevalence between Batch 1 and Batch 2 biological controls

This will only identify potential sequencing contaminants, not extraction contaminants, the same DNA was used for Batch 1 and Batch 2 (from the same Batch 1 extraction)

1. Compare the prevalence of each ASV between the batches

```
m <- prune_samples(sample_sums(BCs)>0, BCs) 

# Convert the otu table in the phyloseq object to presence-absence table 
physeq_milk.pa <- transform_sample_counts(m, function(abund) 1*(abund>0))
#Batch 1
physeq_milk.pa.B1 <- prune_samples(sample_data(physeq_milk.pa)$Batch =="Batch1", physeq_milk.pa) 
physeq_milk.pa.B1
```

```
## phyloseq-class experiment-level object
## otu_table()   OTU Table:         [ 2192 taxa and 9 samples ]
## sample_data() Sample Data:       [ 9 samples by 14 sample variables ]
## tax_table()   Taxonomy Table:    [ 2192 taxa by 7 taxonomic ranks ]
```

```
#Batch 2
physeq_milk.pa.B2 <- prune_samples(sample_data(physeq_milk.pa)$Batch =="Batch2", physeq_milk.pa) 
physeq_milk.pa.B2
```

```
## phyloseq-class experiment-level object
## otu_table()   OTU Table:         [ 2192 taxa and 9 samples ]
## sample_data() Sample Data:       [ 9 samples by 14 sample variables ]
## tax_table()   Taxonomy Table:    [ 2192 taxa by 7 taxonomic ranks ]
```

```
N1 <- 9 # Sample size of Batch 1
N2 <- 9 # Sample size of Batch 2

# Make data.frame of prevalence like before BUT keeping as proprotions instead of Percentages for now
df.pa.milk.prerare <- data.frame(pa.B1=taxa_sums(physeq_milk.pa.B1)/N1,   pa.B2=taxa_sums(physeq_milk.pa.B2)/N2, taxonomy=as(tax_table(physeq_milk.pa), "matrix"))
```

2. Asthetic modifications for plot

```
#remove the prefix p__ from the taxonmy names
df.pa.milk.prerare$Phylum <- gsub("p__", "", df.pa.milk.prerare$taxonomy.Phylum)
#Keep major milk phyla, merge everything else into Other.
df.pa.milk.prerare$Phylum <- ifelse(df.pa.milk.prerare$Phylum == "Proteobacteria" | df.pa.milk.prerare$Phylum == "Actinobacteria" | df.pa.milk.prerare$Phylum == "Firmicutes" | df.pa.milk.prerare$Phylum == "Bacteroidetes", df.pa.milk.prerare$Phylum, "Other")
#Reorder the phyla 
df.pa.milk.prerare$Phylum = factor(df.pa.milk.prerare$Phylum, levels=c('Proteobacteria','Firmicutes', "Actinobacteria", "Bacteroidetes", "Other"))

#Colour pallet for the figure (color by phylum)
cbPalette <- c("navy", "paleturquoise4",  "violetred3", "skyblue1", "goldenrod3")
```

3. Plot the ASV prevalence comparison between between batches for biological controls. Removing taxa BELOW the line (not on the line), based partly on the sample size and standard error of each prevalence level

```
Fig1C <- ggplot() + 
  geom_point(data=df.pa.milk.prerare, aes(x=pa.B1*100, y=pa.B2*100, color=Phylum), alpha=0.75, size=2) + 
  xlab("Batch 1 (n=9)") + 
  ylab("Batch 2 (n=9)") + 
  scale_color_manual(values = cbPalette) +
  theme_minimal()+ 
  theme(legend.position = "right")+
  scale_y_continuous(limits=c(0,100), expand = c(0,0))+
  scale_x_continuous(limits=c(0,101), expand = c(0,0))

Fig1C
```

4. Check the ICC between batches for ASV prevalence in Biological controls - fairly low, this is mostly because there are only 9 samples per batch, prevaelnce values are highly variable with really low sample size, particularly for rare ASVs that may not be present in one batch just by chance.

```
# Check ICC (intraclass correlation), we not only want to identify correlation, but whether values of each taxa between the groups are similar
icc(cbind(df.pa.milk.prerare$pa.B1, df.pa.milk.prerare$pa.B2), "twoway", "agreement")
```

```
##  Single Score Intraclass Correlation
## 
##    Model: twoway 
##    Type : agreement 
## 
##    Subjects = 2192 
##      Raters = 2 
##    ICC(A,1) = 0.715
## 
##  F-Test, H0: r0 = 0 ; H1: r0 > 0 
## F(2191,105) = 6.66 , p = 2.46e-25 
## 
##  95%-Confidence Interval for ICC Population Values:
##   0.637 < ICC < 0.772
```

```
icc(cbind(df.pa.milk.prerare$pa.B1, df.pa.milk.prerare$pa.B2), "twoway", "consistency")
```

```
##  Single Score Intraclass Correlation
## 
##    Model: twoway 
##    Type : consistency 
## 
##    Subjects = 2192 
##      Raters = 2 
##    ICC(C,1) = 0.739
## 
##  F-Test, H0: r0 = 0 ; H1: r0 > 0 
## F(2191,2191) = 6.66 , p = 0 
## 
##  95%-Confidence Interval for ICC Population Values:
##   0.719 < ICC < 0.757
```

## FIGURE 1D: Comparison of ASV average relative abundance between Batch 1 and Batch 2 biological controls

```
m_relative  = transform_sample_counts(BCs, function(x) x*100 / sum(x) ) # plot easier to interpret when in relative abundances

# Make phyloseq object of presence-absence in Batch1 vs. Batch2
physeq_milk.B1 <- prune_samples(sample_data(m_relative)$Batch =="Batch1", m_relative) #9 samples
physeq_milk.B2 <- prune_samples(sample_data(m_relative)$Batch =="Batch2", m_relative) #9 samples 

# Make data.frame of prevalence, with additional taxonomy info attached
df.milk.prerare <- data.frame(pa.B1=taxa_sums(physeq_milk.B1)/9, pa.B2=taxa_sums(physeq_milk.B2)/9,
                      taxonomy=as(tax_table(m_relative), "matrix"))


#Color only by 4 main phyla, make other = black
df.milk.prerare$Phylum <- gsub("p__", "", df.milk.prerare$taxonomy.Phylum)

df.milk.prerare$Phylum <- ifelse(df.milk.prerare$Phylum == "Proteobacteria" | df.milk.prerare$Phylum == "Actinobacteria" | df.milk.prerare$Phylum == "Firmicutes" | df.milk.prerare$Phylum == "Bacteroidetes", df.milk.prerare$Phylum, "Other")

df.milk.prerare$Phylum = factor(df.milk.prerare$Phylum, levels=c('Proteobacteria','Firmicutes', "Actinobacteria", "Bacteroidetes", "Other"))

Fig1D <- ggplot(data=df.milk.prerare, aes(x=pa.B1, y=pa.B2, color=Phylum)) + 
  geom_point(aes(x=pa.B1, y=pa.B2), alpha=0.75, size=2) + 
  xlab("Batch 1 (n=9)") + 
  ylab("Batch 2 (n=9)") + 
  theme(legend.position="right") + 
  scale_color_manual(values = cbPalette) + 
  theme_minimal()+
  scale_y_continuous(expand = c(0.01,0.01), limits = c(0,15))+
  scale_x_continuous(expand = c(0.01,0.01), limits = c(0,15))+
  theme(legend.position = "right")+
  geom_smooth(method='lm', size=0.5, color="black", linetype="dashed", fullrange=TRUE) +
  annotate("segment", x = 0, xend = 15, y = 0, yend = 15, colour = "red", size=0.25)

Fig1D
```

ICC and correlation between batches for ASV relative abundance in Biological controls. Abundances are more precise despite low sample size.

```
# Check ICC (intraclass correlation) 
icc(cbind(df.milk.prerare$pa.B1, df.milk.prerare$pa.B2), "twoway", "agreement")
```

```
##  Single Score Intraclass Correlation
## 
##    Model: twoway 
##    Type : agreement 
## 
##    Subjects = 2192 
##      Raters = 2 
##    ICC(A,1) = 0.994
## 
##  F-Test, H0: r0 = 0 ; H1: r0 > 0 
## F(2191,2191) = 319 , p = 0 
## 
##  95%-Confidence Interval for ICC Population Values:
##   0.993 < ICC < 0.994
```

```
icc(cbind(df.milk.prerare$pa.B1, df.milk.prerare$pa.B2), "twoway", "consistency")
```

```
##  Single Score Intraclass Correlation
## 
##    Model: twoway 
##    Type : consistency 
## 
##    Subjects = 2192 
##      Raters = 2 
##    ICC(C,1) = 0.994
## 
##  F-Test, H0: r0 = 0 ; H1: r0 > 0 
## F(2191,2191) = 319 , p = 0 
## 
##  95%-Confidence Interval for ICC Population Values:
##   0.993 < ICC < 0.994
```

# Identification of potential reagent contaminants using a two-tier strategy including 1) decontam method , 2) comparison of the data structure between batches

## FIGURE 2A: decontam

1. The first contaminant identification method we’ll use is the statistical algorithm implemented in decontam package (Davis et al. Microbiome, 2018) using both the prevalence of ASVs in negative controls compared to samples and DNA concentration to identify contaminants. N = 72 negative controls and 1241 positive samples & controls.

Batch 1: Sequencing negative controls (N=15) Batch 2: DNA extraction (N=21) and sequencing negative controls (N=36)

```
set.seed(147890)

#Negative controls are defined. 
#physeq: Object that contains the original ASV table were output of the QIIME2 pipeline (using the DADA2 denoising algorithm).
sample_data(physeq)$is.neg <- sample_data(physeq)$Smple_type2 == "Negative"

#We used DNA concentration measurement by Picogreen following PCR amplification of 16S rRNA V4 region
contamdf.either <- isContaminant(physeq, method="either", neg="is.neg", conc="DNA_concent_ng_ul", threshold=0.5)
```

```
## Using same threshold value for the frequency and prevalence contaminant identification.
```

```
table(contamdf.either$contaminant)
```

```
## 
## FALSE  TRUE 
## 15698   256
```

2. Figure 2A. Comparison of the prevalence of potential contaminant ASVs identified by decontam in samples vs. negative controls. Code adapted from section 7 of the "Introduction to Decontam" tutorial by B. Callahan (https://benjjneb.github.io/decontam/vignettes/decontam\_intro.html), Access date: 10/05/2020

```
#physeq: Object that contains the original ASV table were output of the QIIME2 pipeline (using the DADA2 denoising algorithm).
# Remove the mock community and the biological controls (plot only milk samples and negative controls)
m <- physeq %>%
  subset_samples(Smple_type2 == "Sample" | Smple_type2 == "Negative") 

# Convert the otu table in the phyloseq object to presence-absence table
m.pa <- transform_sample_counts(m, function(abund) 1*(abund>0))
#Identify the negative controls
m.pa.neg <- prune_samples(sample_data(m.pa)$is.neg ==TRUE, m.pa)
m.pa.neg
```

```
## phyloseq-class experiment-level object
## otu_table()   OTU Table:         [ 15954 taxa and 72 samples ]
## sample_data() Sample Data:       [ 72 samples by 15 sample variables ]
## tax_table()   Taxonomy Table:    [ 15954 taxa by 7 taxonomic ranks ]
```

```
#Identify the samples (including the positive controls)
m.pa.pos <- prune_samples(sample_data(m.pa)$is.neg ==FALSE, m.pa)
m.pa.pos
```

```
## phyloseq-class experiment-level object
## otu_table()   OTU Table:         [ 15954 taxa and 1190 samples ]
## sample_data() Sample Data:       [ 1190 samples by 15 sample variables ]
## tax_table()   Taxonomy Table:    [ 15954 taxa by 7 taxonomic ranks ]
```

```
# make data.frame of prevalence in positive and negative samples for contaminants (using 0.05 and NoHb)
df.pa <- data.frame(pa.pos=taxa_sums(m.pa.pos)*100/1190, pa.neg=taxa_sums(m.pa.neg)*100/72,contaminant=contamdf.either$contaminant, taxonomy=as(tax_table(m.pa), "matrix"))

#plot the figure
Fig2A <- ggplot(data=df.pa, aes(x=pa.neg, y=pa.pos, color=contaminant)) + 
       geom_point() +
       xlab("Negative controls (n=72)") + 
       ylab("Positive samples (n=1190)") + 
       scale_color_manual(values=c("#237a72" ,"violetred3"))+
       scale_x_continuous(limits = c(0,101), expand = c(0,0))+
       scale_y_continuous(limits = c(0,100), expand = c(0,0))+
       guides(color = guide_legend(reverse=T, title="Contaminant"))+
       theme_minimal()+
       theme(legend.position = "right")

Fig2A
```

```
# 14,817 ASVs present in either samples or negative controls overall (ie. shown in Figure 2A, excludes samples in mocks and BCs)
sum(df.pa$pa.pos >0 | df.pa$pa.neg >0)
```

```
## [1] 14817
```

3. Remove these potential contaminants from the phyloseq object:

```
ps.noncontam <- prune_taxa(!contamdf.either$contaminant, physeq)
ps.noncontam
```

```
## phyloseq-class experiment-level object
## otu_table()   OTU Table:         [ 15698 taxa and 1316 samples ]
## sample_data() Sample Data:       [ 1316 samples by 15 sample variables ]
## tax_table()   Taxonomy Table:    [ 15698 taxa by 7 taxonomic ranks ]
```

4. Additional checks of these 256 potential contaminants, identify the total number of reads contributed by these ASVs and identify their average prevalence within different data subsets

```
# phyloseq obj only containing potential contaminants identified by decontam
ps_contam <- prune_taxa(contamdf.either$contaminant, physeq)
ps_contam
```

```
## phyloseq-class experiment-level object
## otu_table()   OTU Table:         [ 256 taxa and 1316 samples ]
## sample_data() Sample Data:       [ 1316 samples by 15 sample variables ]
## tax_table()   Taxonomy Table:    [ 256 taxa by 7 taxonomic ranks ]
```

```
# interested in samples only 
ps_contam_samps <- subset_samples(ps_contam, Smple_type2 == "Sample")
ps_samps_total <- subset_samples(physeq, Smple_type2 == "Sample")


# Total reads contributed by contaminants in samples
decontam_reads <- sample_sums(ps_contam_samps)
# Total reads overall, in samples
total_reads <- sample_sums(ps_samps_total)


# sample data
sdf_contam <- as(sample_data(ps_contam_samps), "data.frame")

sdf_contam2 <- data.frame(sdf_contam, decon_contam = decontam_reads, total_preDecon = total_reads) # can merge this way b/c same order

sdf_contam2$Percent_contam1 <- sdf_contam2$decon_contam*100/sdf_contam2$total_preDecon


# Average Percent of total reads identified as contaminants
summary(sdf_contam2$Percent_contam1)
```

```
##      Min.   1st Qu.    Median      Mean   3rd Qu.      Max.      NA's 
##   0.00000   0.00000   0.01673   0.72647   0.09459 100.00000         1
```

```
# Average % of original reads stratified by batch and run
data_summary_decontam <- data_summary(sdf_contam2, varname = "Percent_contam1", groupnames = c("Batch", "Run"))
data_summary_decontam
```

```
##    Batch   Run Percent_contam1       sd      median min       max        IQR
## 1 Batch1    R1       0.6186606 5.482340 0.019599931   0  79.27719 0.07680625
## 2 Batch1    R2       0.1318938 1.013857 0.032557382   0  14.77350 0.07825543
## 3 Batch2 Run52       1.0836402 5.605606 0.010509883   0  54.83871 0.13658909
## 4 Batch2 Run53       1.1603135 7.567019 0.006407384   0 100.00000 0.13716884
## 5 Batch2 Run54       0.5289234 5.707629 0.007585034   0  90.69476 0.09323308
##   Q1.25%     Q3.75%        sem N not 0 % N not 0
## 1      0 0.07680625 0.37389250     146  67.90698
## 2      0 0.07825543 0.06946835     155  72.76995
## 3      0 0.13658909 0.35311996     138  54.76190
## 4      0 0.13716884 0.47386512     136        NA
## 5      0 0.09323308 0.35742561     139  54.50980
```

```
# Identify the Average prevalence of a contaminant ASV overall and stratified by Run

## Prevalence is just the % of N not 0, so using data_summary again, but for this another groupname will be the ASVID
ps_contam_otu <- as.data.frame(t(as(otu_table(ps_contam_samps), "matrix")))


ps_contam_otu_meta <- merge(sdf_contam, ps_contam_otu, by="row.names")

ps_contam_otu_meta_melt <- melt(ps_contam_otu_meta, measure.vars = colnames(ps_contam_otu), id.vars = c("Run")) # variable = ASVid


# Prevalence is % of N not 0 - each contaminant ASV has a prevalence
data_summary_decontam_runStrat <- data_summary(ps_contam_otu_meta_melt, varname = "value", groupnames = c("variable", "Run"))
colnames(data_summary_decontam_runStrat)[13]
```

```
## [1] "% N not 0"
```

```
colnames(data_summary_decontam_runStrat)[13] <- "Prevalence"


## To get the average prevalence ( % of N not 0) of a contaminant ASV per group for each Run now, do another data_summary
av_prev_decontam_runStrat <- data_summary(data_summary_decontam_runStrat, varname = "Prevalence", groupnames = c("Run"))
av_prev_decontam_runStrat # column "Prevaelnce", shows the average prevaelnce of a contaminant identified by decontam in percent
```

```
##     Run Prevalence        sd    median min       max       IQR Q1.25%    Q3.75%
## 1    R1  0.9920058 1.4861715 0.4651163   0 12.093023 1.3953488      0 1.3953488
## 2    R2  0.6767165 1.2345008 0.0000000   0  7.511737 0.9389671      0 0.9389671
## 3 Run52  0.4479787 0.8237170 0.0000000   0  5.555556 0.3968254      0 0.3968254
## 4 Run53  0.4227941 0.8773836 0.0000000   0  7.450980 0.3921569      0 0.3921569
## 5 Run54  0.3676471 0.7923464 0.0000000   0  7.058824 0.3921569      0 0.3921569
##          sem N not 0 % N not 0
## 1 0.09288572     145  56.64062
## 2 0.07715630     125  48.82812
## 3 0.05148231     112  43.75000
## 4 0.05483647     100  39.06250
## 5 0.04952165     100  39.06250
```

```
## Overall average prevalence/etc. of a contaminant ASV
data_summary_decontam_byASV <- data_summary(ps_contam_otu_meta_melt, varname = "value", groupnames = c("variable"))
summary(data_summary_decontam_byASV$`% N not 0`)
```

```
##    Min. 1st Qu.  Median    Mean 3rd Qu.    Max. 
## 0.00000 0.08403 0.25210 0.56460 0.75630 6.05042
```

## Additional pre-processing following contaminant identification using decontam

1. Removal of reads belonging to Cyanobacteria, mitochnodria, Chloroplast and unassigned taxonomy at the kingdom level

```
#ps.noncontam: Object made by removing contaminating ASVs identified by the decontam.

ps.noncontam2 <- ps.noncontam %>%
  subset_taxa(
    Kingdom != "Unassigned" &
    Phylum != "p__Cyanobacteria" &
    Family  != "f__mitochondria" &
    Class   != "c__Chloroplast"
  )
ps.noncontam2
```

```
## phyloseq-class experiment-level object
## otu_table()   OTU Table:         [ 14914 taxa and 1316 samples ]
## sample_data() Sample Data:       [ 1316 samples by 15 sample variables ]
## tax_table()   Taxonomy Table:    [ 14914 taxa by 7 taxonomic ranks ]
```

2. Removal of controls from the phyloseq

```
#ps.noncontam: Object made by removing contaminating ASVs identified by the decontam.
physeq_sample2 <- ps.noncontam2 %>%
  subset_samples(Smple_type2 == "Sample")
#Excluding samples with 0 sequencing reads 
physeq_sample2 <- prune_taxa(taxa_sums(physeq_sample2)>0, physeq_sample2)

physeq_sample2
```

```
## phyloseq-class experiment-level object
## otu_table()   OTU Table:         [ 13348 taxa and 1190 samples ]
## sample_data() Sample Data:       [ 1190 samples by 15 sample variables ]
## tax_table()   Taxonomy Table:    [ 13348 taxa by 7 taxonomic ranks ]
```

# Assessment of data structure to identify potential contaminants (Figure S1)

Comparisons between sequencing runs within batches to identify possible run-specific contaminants and comparisons between batches to identify batch-specific contaminants

Preprocessing dataset used to identify potential contaminants by assessing the data structure (Figure 2B and Figure S1)

```
m <- prune_samples(sample_sums(physeq_sample2)>0, physeq_sample2) #1188 samples [2 samples with 0 reads from batch2]

# Convert the otu table in the phyloseq object to presence-absence table 
physeq_milk.pa <- transform_sample_counts(m, function(abund) 1*(abund>0))
```

## Figure S1A: ASV prevalence comparison between runs within Batch 1

1. Compare the prevalence of each ASV between Run 1 and Run 2

```
# Run 1, Batch 1
physeq_milk.pa.B1.R1 <- prune_samples(sample_data(physeq_milk.pa)$Run =="R1", physeq_milk.pa) 
physeq_milk.pa.B1.R1
```

```
## phyloseq-class experiment-level object
## otu_table()   OTU Table:         [ 13348 taxa and 215 samples ]
## sample_data() Sample Data:       [ 215 samples by 148 sample variables ]
## tax_table()   Taxonomy Table:    [ 13348 taxa by 7 taxonomic ranks ]
```

```
# Run 2, Batch 1
physeq_milk.pa.B1.R2 <- prune_samples(sample_data(physeq_milk.pa)$Run =="R2", physeq_milk.pa) 
physeq_milk.pa.B1.R2
```

```
## phyloseq-class experiment-level object
## otu_table()   OTU Table:         [ 13348 taxa and 213 samples ]
## sample_data() Sample Data:       [ 213 samples by 148 sample variables ]
## tax_table()   Taxonomy Table:    [ 13348 taxa by 7 taxonomic ranks ]
```

```
N1 <- 215 # sample size in Run 1
N2 <- 213 # sample size in Run 2
 
# Make data.frame of prevalence, with additional taxonomy info at phylum level attached.
df.pa.milk.prerare <- data.frame(pa.B1=taxa_sums(physeq_milk.pa.B1.R1)/N1,   pa.B2=taxa_sums(physeq_milk.pa.B1.R2)/N2, taxonomy=as(tax_table(physeq_milk.pa), "matrix"))
## won't actually show those that have prevalence of 0 in both

# 8,700 ASVs present in either run of batch 1
sum(df.pa.milk.prerare$pa.B1 >0 | df.pa.milk.prerare$pa.B2 >0)
```

```
## [1] 8700
```

2. To make the sloped line in the prevalence plot, use the Structure\_comp function on all possible proportions in intervals of 0.001, given sample sizes N1 and N2 specified in the above chunk

```
# Generic use of the Structure_comp function on xs (all possible proportions in intervals of 0.001) given a specific value of N1 and N2 specified above
xs <- seq(0, 1, by = 0.001)  # All possible proportions in intervals of 0.001
ysmax <- Structure_comp(xs)   # ysmax is the MINUMUM a proportion can be in one batch, given a certain proportion in another batch (xs). In the case of extremely low values of xs, this can be negative (this happens more often with really low N, i.e. cannot actually say that the taxa is a contaminant, prevalence too low and/or N too low). 
ysmin <- rep(0, length(xs))
df2 <- data.frame(xs, ysmin, ysmax)
```

3. Asthetic modifications for plot

```
#remove the prefix p__ from the taxonmy names
df.pa.milk.prerare$Phylum <- gsub("p__", "", df.pa.milk.prerare$taxonomy.Phylum)
#Keep major milk phyla, merge everything else into Other.
df.pa.milk.prerare$Phylum <- ifelse(df.pa.milk.prerare$Phylum == "Proteobacteria" | df.pa.milk.prerare$Phylum == "Actinobacteria" | df.pa.milk.prerare$Phylum == "Firmicutes" | df.pa.milk.prerare$Phylum == "Bacteroidetes", df.pa.milk.prerare$Phylum, "Other")
#Reorder the phyla 
df.pa.milk.prerare$Phylum = factor(df.pa.milk.prerare$Phylum, levels=c('Proteobacteria','Firmicutes', "Actinobacteria", "Bacteroidetes", "Other"))

#Colour pallet for the figure (color by phylum)
cbPalette <- c("navy", "paleturquoise4",  "violetred3", "skyblue1", "goldenrod3")
```

4. Plot the ASV prevalence comparison between runs within Batch 1. Taxa BELOW the orange line will be defined as potential contaminants based on this prevalence comparison

```
FigS1A <- ggplot() + 
  geom_ribbon(aes(x=xs*100, ymin=ysmin*100, ymax=ysmax*100), data=df2, fill="gray80")+
  geom_ribbon(aes(y=xs*100, xmin=ysmin*100, xmax=ysmax*100), data=df2, fill="gray80")+
  geom_point(data=df.pa.milk.prerare, aes(x=pa.B1*100, y=pa.B2*100, color=Phylum), alpha=0.75, size=2) + 
  xlab("Run 1 (n=215)") + 
  ylab("Run 2 (n=213)") + 
  scale_color_manual(values = cbPalette) +
  theme_minimal()+ 
  theme(legend.position = "right")+
  geom_line(data = df2, aes(x = xs*100, y = ysmax*100), colour="#e6800b") + 
  geom_line(data = df2, aes(x = ysmax*100, y = xs*100), colour="#e6800b") +
  scale_y_continuous(limits=c(0,100), expand = c(0,0))+
  scale_x_continuous(limits=c(0,101), expand = c(0,0))

FigS1A
```

5. Make lists of potential contaminants specific to Run 1 and Run 2 of Batch 1 based on the prevalence comparison shown in Figure S1A

```
df.pa.milk.prerare$Formula_B1 <- Structure_comp(df.pa.milk.prerare$pa.B1) # Given prevalence in Run1
df.pa.milk.prerare$Formula_B2 <- Structure_comp(df.pa.milk.prerare$pa.B2)# Given prevalence in Run2

ASV_Discard_R1_B1 <- subset(df.pa.milk.prerare, pa.B2 < Formula_B1) # Potential contaminants in Run 2 (shaded area on x-axis)
dim(ASV_Discard_R1_B1) # 167, Most/all of these are rare
```

```
## [1] 167  12
```

```
ASV_Discard_R2_B1 <- subset(df.pa.milk.prerare, pa.B1 < Formula_B2) # Potential contaminants below the "minimum prevalence" in Run 1 (shaded area on y-axis)
dim(ASV_Discard_R2_B1) # 31, Most/all of these are rare
```

```
## [1] 31 12
```

6. Check the ICC between batches for the prevalence of ASVs

```
icc(cbind(df.pa.milk.prerare$pa.B1, df.pa.milk.prerare$pa.B2), "twoway", "agreement")
```

```
##  Single Score Intraclass Correlation
## 
##    Model: twoway 
##    Type : agreement 
## 
##    Subjects = 13348 
##      Raters = 2 
##    ICC(A,1) = 0.993
## 
##  F-Test, H0: r0 = 0 ; H1: r0 > 0 
## F(13347,10177) = 270 , p = 0 
## 
##  95%-Confidence Interval for ICC Population Values:
##   0.992 < ICC < 0.993
```

```
icc(cbind(df.pa.milk.prerare$pa.B1, df.pa.milk.prerare$pa.B2), "twoway", "consistency")
```

```
##  Single Score Intraclass Correlation
## 
##    Model: twoway 
##    Type : consistency 
## 
##    Subjects = 13348 
##      Raters = 2 
##    ICC(C,1) = 0.993
## 
##  F-Test, H0: r0 = 0 ; H1: r0 > 0 
## F(13347,13347) = 270 , p = 0 
## 
##  95%-Confidence Interval for ICC Population Values:
##   0.992 < ICC < 0.993
```

## FIGURE S1B: ASV prevalence comparison between Run 1 and Run 2 within Batch 2

1. Compare the prevalence of each ASV between Run 1 and Run 2 of Batch 2

```
# Run 1, Batch 2
physeq_milk.pa.B2.R1 <- prune_samples(sample_data(physeq_milk.pa)$Run =="Run52", physeq_milk.pa) 
physeq_milk.pa.B2.R1 # 252 samples
```

```
## phyloseq-class experiment-level object
## otu_table()   OTU Table:         [ 13348 taxa and 252 samples ]
## sample_data() Sample Data:       [ 252 samples by 148 sample variables ]
## tax_table()   Taxonomy Table:    [ 13348 taxa by 7 taxonomic ranks ]
```

```
# Run 2, Batch 2
physeq_milk.pa.B2.R2 <- prune_samples(sample_data(physeq_milk.pa)$Run =="Run53", physeq_milk.pa) 
physeq_milk.pa.B2.R2 # 253 samples
```

```
## phyloseq-class experiment-level object
## otu_table()   OTU Table:         [ 13348 taxa and 253 samples ]
## sample_data() Sample Data:       [ 253 samples by 148 sample variables ]
## tax_table()   Taxonomy Table:    [ 13348 taxa by 7 taxonomic ranks ]
```

```
N1 <- 252 # N of Run 1
N2 <- 253 # N of Run 2


# Make data.frame of prevalence, with additional taxonomy info at phylum level attached.
df.pa.milk.prerare <- data.frame(pa.B1=taxa_sums(physeq_milk.pa.B2.R1)/N1,   pa.B2=taxa_sums(physeq_milk.pa.B2.R2)/N2, taxonomy=as(tax_table(physeq_milk.pa), "matrix"))

# 5226 ASVs present in either Run 1 or Run 2 of batch 2
sum(df.pa.milk.prerare$pa.B1 >0 | df.pa.milk.prerare$pa.B2 >0)
```

```
## [1] 5226
```

2. To make the sloped line in the prevalence plot, use the Structure\_comp function on all possible proportions in intervals of 0.001, given sample sizes N1 and N2 specified in the above chunk

```
# Generic use of the Structure_comp function on xs (all possible proportions in intervals of 0.001) given a specific value of N1 and N2 specified above
xs <- seq(0, 1, by = 0.001)  # All possible proportions in intervals of 0.001
ysmax <- Structure_comp(xs)   # ysmax is the MINUMUM a proportion can be in one batch, given a certain proportion in another batch (xs). In the case of extremely low values of xs, this can be negative (this happens more often with really low N, i.e. cannot actually say that the taxa is a contaminant, prevalence too low and/or N too low). 
ysmin <- rep(0, length(xs))
df2 <- data.frame(xs, ysmin, ysmax)
```

3. Asthetic modifications for plot

```
#remove the prefix p__ from the taxonmy names
df.pa.milk.prerare$Phylum <- gsub("p__", "", df.pa.milk.prerare$taxonomy.Phylum)
#Keep major milk phyla, merge everything else into Other.
df.pa.milk.prerare$Phylum <- ifelse(df.pa.milk.prerare$Phylum == "Proteobacteria" | df.pa.milk.prerare$Phylum == "Actinobacteria" | df.pa.milk.prerare$Phylum == "Firmicutes" | df.pa.milk.prerare$Phylum == "Bacteroidetes", df.pa.milk.prerare$Phylum, "Other")
#Reorder the phyla 
df.pa.milk.prerare$Phylum = factor(df.pa.milk.prerare$Phylum, levels=c('Proteobacteria','Firmicutes', "Actinobacteria", "Bacteroidetes", "Other"))

#Colour pallet for the figure (color by phylum)
cbPalette <- c("navy", "paleturquoise4",  "violetred3", "skyblue1", "goldenrod3")
```

4. Plot the ASV prevalence comparison between Run 1 and Run 2 within Batch 2. Taxa BELOW the orange line will be defined as potential contaminants based on this prevalence comparison

```
FigS1B <- ggplot() + 
  geom_ribbon(aes(x=xs*100, ymin=ysmin*100, ymax=ysmax*100), data=df2, fill="gray80")+
  geom_ribbon(aes(y=xs*100, xmin=ysmin*100, xmax=ysmax*100), data=df2, fill="gray80")+
  geom_point(data=df.pa.milk.prerare, aes(x=pa.B1*100, y=pa.B2*100, color=Phylum), alpha=0.75, size=2) + 
  xlab("Run 1 (n=252)") + 
  ylab("Run 2 (n=253)") + 
  scale_color_manual(values = cbPalette) +
  theme_minimal()+ 
  theme(legend.position = "right")+
  geom_line(data = df2, aes(x = xs*100, y = ysmax*100), colour="#e6800b") + 
  geom_line(data = df2, aes(x = ysmax*100, y = xs*100), colour="#e6800b") +
  scale_y_continuous(limits=c(0,100), expand = c(0,0))+
  scale_x_continuous(limits=c(0,101), expand = c(0,0))

FigS1B
```

5. Make lists of potential contaminants specific to Run 1 and Run 2 of Batch 2 based on the prevalence comparison shown in Figure S1B

```
df.pa.milk.prerare$Formula_B1 <- Structure_comp(df.pa.milk.prerare$pa.B1) # Given prevalence in Run1
df.pa.milk.prerare$Formula_B2 <- Structure_comp(df.pa.milk.prerare$pa.B2)# Given prevalence in Run2

ASV_Discard_R1_B2 <- subset(df.pa.milk.prerare, pa.B2 < Formula_B1) # ASVs below the "minimum prevalence" in Run 2 (shaded area on x-axis)
dim(ASV_Discard_R1_B2) # 16, Most of these are rare
```

```
## [1] 16 12
```

```
ASV_Discard_R2_B2 <- subset(df.pa.milk.prerare, pa.B1 < Formula_B2) # ASVs below the "minimum prevalence" in Run 1 (shaded area on y-axis)
dim(ASV_Discard_R2_B2) # 17, Most of these are rare
```

```
## [1] 17 12
```

6. Check the ICC between batches for the prevalence of ASVs

```
icc(cbind(df.pa.milk.prerare$pa.B1, df.pa.milk.prerare$pa.B2), "twoway", "agreement")
```

```
##  Single Score Intraclass Correlation
## 
##    Model: twoway 
##    Type : agreement 
## 
##    Subjects = 13348 
##      Raters = 2 
##    ICC(A,1) = 0.975
## 
##  F-Test, H0: r0 = 0 ; H1: r0 > 0 
## F(13347,11456) = 78.5 , p = 0 
## 
##  95%-Confidence Interval for ICC Population Values:
##   0.974 < ICC < 0.976
```

```
icc(cbind(df.pa.milk.prerare$pa.B1, df.pa.milk.prerare$pa.B2), "twoway", "consistency")
```

```
##  Single Score Intraclass Correlation
## 
##    Model: twoway 
##    Type : consistency 
## 
##    Subjects = 13348 
##      Raters = 2 
##    ICC(C,1) = 0.975
## 
##  F-Test, H0: r0 = 0 ; H1: r0 > 0 
## F(13347,13347) = 78.5 , p = 0 
## 
##  95%-Confidence Interval for ICC Population Values:
##   0.974 < ICC < 0.976
```

## FIGURE S1C: ASV prevalence comparison between Run 1 and Run 3 within Batch 2

1. Compare the prevalence of each ASV between Run 1 and Run 3 of Batch 2

```
# Run 1, Batch 2
physeq_milk.pa.B2.R1 <- prune_samples(sample_data(physeq_milk.pa)$Run =="Run52", physeq_milk.pa) 
physeq_milk.pa.B2.R1 # 252 samples
```

```
## phyloseq-class experiment-level object
## otu_table()   OTU Table:         [ 13348 taxa and 252 samples ]
## sample_data() Sample Data:       [ 252 samples by 148 sample variables ]
## tax_table()   Taxonomy Table:    [ 13348 taxa by 7 taxonomic ranks ]
```

```
# Run 3, Batch 2
physeq_milk.pa.B2.R3 <- prune_samples(sample_data(physeq_milk.pa)$Run =="Run54", physeq_milk.pa) 
physeq_milk.pa.B2.R3 # 255 samples
```

```
## phyloseq-class experiment-level object
## otu_table()   OTU Table:         [ 13348 taxa and 255 samples ]
## sample_data() Sample Data:       [ 255 samples by 148 sample variables ]
## tax_table()   Taxonomy Table:    [ 13348 taxa by 7 taxonomic ranks ]
```

```
N1 <- 252 # N of RUN 1
N2 <- 255 # N of RUN 3

# Make data.frame of prevalence, with additional taxonomy info at phylum level attached.
df.pa.milk.prerare <- data.frame(pa.B1=taxa_sums(physeq_milk.pa.B2.R1)/N1,   pa.B2=taxa_sums(physeq_milk.pa.B2.R3)/N2, taxonomy=as(tax_table(physeq_milk.pa), "matrix"))

# 4659 ASVs present in either run 1 or run 3 of batch 2
sum(df.pa.milk.prerare$pa.B1 >0 | df.pa.milk.prerare$pa.B2 >0)
```

```
## [1] 4659
```

2. To make the sloped line in the prevalence plot, use the Structure\_comp function on all possible proportions in intervals of 0.001, given sample sizes N1 and N2 specified in the above chunk

```
# Generic use of the Structure_comp function on xs (all possible proportions in intervals of 0.001) given a specific value of N1 and N2 specified above
xs <- seq(0, 1, by = 0.001)  # All possible proportions in intervals of 0.001
ysmax <- Structure_comp(xs)   # ysmax is the MINUMUM a proportion can be in one batch, given a certain proportion in another batch (xs). In the case of extremely low values of xs, this can be negative (this happens more often with really low N, i.e. cannot actually say that the taxa is a contaminant, prevalence too low and/or N too low). 
ysmin <- rep(0, length(xs))
df2 <- data.frame(xs, ysmin, ysmax)
```

3. Asthetic modifications for plot

```
#remove the prefix p__ from the taxonmy names
df.pa.milk.prerare$Phylum <- gsub("p__", "", df.pa.milk.prerare$taxonomy.Phylum)
#Keep major milk phyla, merge everything else into Other.
df.pa.milk.prerare$Phylum <- ifelse(df.pa.milk.prerare$Phylum == "Proteobacteria" | df.pa.milk.prerare$Phylum == "Actinobacteria" | df.pa.milk.prerare$Phylum == "Firmicutes" | df.pa.milk.prerare$Phylum == "Bacteroidetes", df.pa.milk.prerare$Phylum, "Other")
#Reorder the phyla 
df.pa.milk.prerare$Phylum = factor(df.pa.milk.prerare$Phylum, levels=c('Proteobacteria','Firmicutes', "Actinobacteria", "Bacteroidetes", "Other"))

#Colour pallet for the figure (color by phylum)
cbPalette <- c("navy", "paleturquoise4",  "violetred3", "skyblue1", "goldenrod3")
```

4. Plot the ASV prevalence comparison between Run 1 and Run 3 within Batch 2. Taxa BELOW the orange line will be defined as potential contaminants based on this prevalence comparison

```
FigS1C <- ggplot() + 
  geom_ribbon(aes(x=xs*100, ymin=ysmin*100, ymax=ysmax*100), data=df2, fill="gray80")+
  geom_ribbon(aes(y=xs*100, xmin=ysmin*100, xmax=ysmax*100), data=df2, fill="gray80")+
  geom_point(data=df.pa.milk.prerare, aes(x=pa.B1*100, y=pa.B2*100, color=Phylum), alpha=0.75, size=2) + 
  xlab("Run 1 (n=252)") + 
  ylab("Run 3 (n=255)") + 
  scale_color_manual(values = cbPalette) +
  theme_minimal()+ 
  theme(legend.position = "right")+
  geom_line(data = df2, aes(x = xs*100, y = ysmax*100), colour="#e6800b") + 
  geom_line(data = df2, aes(x = ysmax*100, y = xs*100), colour="#e6800b") +
  scale_y_continuous(limits=c(0,100), expand = c(0,0))+
  scale_x_continuous(limits=c(0,101), expand = c(0,0))

FigS1C
```

5. Make lists of potential contaminants specific to Run 1 and Run 3 of Batch 2 based on the prevalence comparison shown in Figure S1C

```
df.pa.milk.prerare$Formula_B1 <- Structure_comp(df.pa.milk.prerare$pa.B1) # Given prevalence in Run1
df.pa.milk.prerare$Formula_B2 <- Structure_comp(df.pa.milk.prerare$pa.B2)# Given prevalence in Run3

ASV_Discard_R1vR3_B2 <- subset(df.pa.milk.prerare, pa.B2 < Formula_B1) # ASVs below the "minimum prevalence" in Run 3 (shaded area on x-axis)
dim(ASV_Discard_R1vR3_B2) # 11, these are rare
```

```
## [1] 11 12
```

```
ASV_Discard_R3vR1_B2 <- subset(df.pa.milk.prerare, pa.B1 < Formula_B2) # ASVs below the "minimum prevalence" in Run 1 (shaded area on y-axis)
dim(ASV_Discard_R3vR1_B2) # 10, these are rare
```

```
## [1] 10 12
```

6. Check the ICC between batches for the prevalence of ASVs

```
icc(cbind(df.pa.milk.prerare$pa.B1, df.pa.milk.prerare$pa.B2), "twoway", "agreement")
```

```
##  Single Score Intraclass Correlation
## 
##    Model: twoway 
##    Type : agreement 
## 
##    Subjects = 13348 
##      Raters = 2 
##    ICC(A,1) = 0.977
## 
##  F-Test, H0: r0 = 0 ; H1: r0 > 0 
## F(13347,13348) = 86.8 , p = 0 
## 
##  95%-Confidence Interval for ICC Population Values:
##   0.976 < ICC < 0.978
```

```
icc(cbind(df.pa.milk.prerare$pa.B1, df.pa.milk.prerare$pa.B2), "twoway", "consistency")
```

```
##  Single Score Intraclass Correlation
## 
##    Model: twoway 
##    Type : consistency 
## 
##    Subjects = 13348 
##      Raters = 2 
##    ICC(C,1) = 0.977
## 
##  F-Test, H0: r0 = 0 ; H1: r0 > 0 
## F(13347,13347) = 86.8 , p = 0 
## 
##  95%-Confidence Interval for ICC Population Values:
##   0.976 < ICC < 0.978
```

## FIGURE S1D: ASV prevalence comparison between Run 2 and Run 3 within Batch 2

1. Compare the prevalence of each ASV between Run 2 and Run 3 of Batch 2

```
# Run 2, Batch 2
physeq_milk.pa.B2.R2 <- prune_samples(sample_data(physeq_milk.pa)$Run =="Run53", physeq_milk.pa) 
physeq_milk.pa.B2.R2 # 253 samples
```

```
## phyloseq-class experiment-level object
## otu_table()   OTU Table:         [ 13348 taxa and 253 samples ]
## sample_data() Sample Data:       [ 253 samples by 148 sample variables ]
## tax_table()   Taxonomy Table:    [ 13348 taxa by 7 taxonomic ranks ]
```

```
# Run 3, Batch 2
physeq_milk.pa.B2.R3 <- prune_samples(sample_data(physeq_milk.pa)$Run =="Run54", physeq_milk.pa) 
physeq_milk.pa.B2.R3 # 255 samples
```

```
## phyloseq-class experiment-level object
## otu_table()   OTU Table:         [ 13348 taxa and 255 samples ]
## sample_data() Sample Data:       [ 255 samples by 148 sample variables ]
## tax_table()   Taxonomy Table:    [ 13348 taxa by 7 taxonomic ranks ]
```

```
N1 <- 253 # N of Run 2
N2 <- 255 # N of RUN 3


# Make data.frame of prevalence, with additional taxonomy info at phylum level attached.
df.pa.milk.prerare <- data.frame(pa.B1=taxa_sums(physeq_milk.pa.B2.R2)/N1,   pa.B2=taxa_sums(physeq_milk.pa.B2.R3)/N2, taxonomy=as(tax_table(physeq_milk.pa), "matrix"))

# 5258 ASVs present in either r1 or r3 of batch 2
sum(df.pa.milk.prerare$pa.B1 >0 | df.pa.milk.prerare$pa.B2 >0)
```

```
## [1] 5258
```

2. To make the sloped line in the prevalence plot, use the Structure\_comp function on all possible proportions in intervals of 0.001, given sample sizes N1 and N2 specified in the above chunk

```
# Generic use of the Structure_comp function on xs (all possible proportions in intervals of 0.001) given a specific value of N1 and N2 specified above
xs <- seq(0, 1, by = 0.001)  # All possible proportions in intervals of 0.001
ysmax <- Structure_comp(xs)   # ysmax is the MINUMUM a proportion can be in one batch, given a certain proportion in another batch (xs). In the case of extremely low values of xs, this can be negative (this happens more often with really low N, i.e. cannot actually say that the taxa is a contaminant, prevalence too low and/or N too low). 
ysmin <- rep(0, length(xs))
df2 <- data.frame(xs, ysmin, ysmax)
```

3. Asthetic modifications for plot

```
#remove the prefix p__ from the taxonmy names
df.pa.milk.prerare$Phylum <- gsub("p__", "", df.pa.milk.prerare$taxonomy.Phylum)
#Keep major milk phyla, merge everything else into Other.
df.pa.milk.prerare$Phylum <- ifelse(df.pa.milk.prerare$Phylum == "Proteobacteria" | df.pa.milk.prerare$Phylum == "Actinobacteria" | df.pa.milk.prerare$Phylum == "Firmicutes" | df.pa.milk.prerare$Phylum == "Bacteroidetes", df.pa.milk.prerare$Phylum, "Other")
#Reorder the phyla 
df.pa.milk.prerare$Phylum = factor(df.pa.milk.prerare$Phylum, levels=c('Proteobacteria','Firmicutes', "Actinobacteria", "Bacteroidetes", "Other"))

#Colour pallet for the figure (color by phylum)
cbPalette <- c("navy", "paleturquoise4",  "violetred3", "skyblue1", "goldenrod3")
```

4. Plot the ASV prevalence comparison between Run 2 and Run 3 within Batch 2. Taxa BELOW the orange line will be defined as potential contaminants based on this prevalence comparison

```
FigS1D <- ggplot() + 
  geom_ribbon(aes(x=xs*100, ymin=ysmin*100, ymax=ysmax*100), data=df2, fill="gray80")+
  geom_ribbon(aes(y=xs*100, xmin=ysmin*100, xmax=ysmax*100), data=df2, fill="gray80")+
  geom_point(data=df.pa.milk.prerare, aes(x=pa.B1*100, y=pa.B2*100, color=Phylum), alpha=0.75, size=2) + 
  xlab("Run 2 (n=253)") + 
  ylab("Run 3 (n=255)") + 
  scale_color_manual(values = cbPalette) +
  theme_minimal()+ 
  theme(legend.position = "right")+
  geom_line(data = df2, aes(x = xs*100, y = ysmax*100), colour="#e6800b") + 
  geom_line(data = df2, aes(x = ysmax*100, y = xs*100), colour="#e6800b") +
  scale_y_continuous(limits=c(0,100), expand = c(0,0))+
  scale_x_continuous(limits=c(0,101), expand = c(0,0))

FigS1D
```

5. Make lists of potential contaminants specific to Run 2 and Run 3 of Batch 2 based on the prevalence comparison shown in Figure S1D

```
df.pa.milk.prerare$Formula_B1 <- Structure_comp(df.pa.milk.prerare$pa.B1) # Given prevalence in Run 2
df.pa.milk.prerare$Formula_B2 <- Structure_comp(df.pa.milk.prerare$pa.B2)# Given prevalence in Run 3

ASV_Discard_R2vR3_B2 <- subset(df.pa.milk.prerare, pa.B2 < Formula_B1) # ASVs below the "minimum prevalence" in Run 3 (shaded area on x-axis)
dim(ASV_Discard_R2vR3_B2) # 21
```

```
## [1] 21 12
```

```
ASV_Discard_R3vR2_B2 <- subset(df.pa.milk.prerare, pa.B1 < Formula_B2) # ASVs below the "minimum prevalence" in Run 2 (shaded area on y-axis)
dim(ASV_Discard_R3vR2_B2) # 11
```

```
## [1] 11 12
```

6. Check the ICC between batches for the prevalence of ASVs

```
icc(cbind(df.pa.milk.prerare$pa.B1, df.pa.milk.prerare$pa.B2), "twoway", "agreement")
```

```
##  Single Score Intraclass Correlation
## 
##    Model: twoway 
##    Type : agreement 
## 
##    Subjects = 13348 
##      Raters = 2 
##    ICC(A,1) = 0.978
## 
##  F-Test, H0: r0 = 0 ; H1: r0 > 0 
## F(13347,10389) = 90.3 , p = 0 
## 
##  95%-Confidence Interval for ICC Population Values:
##   0.977 < ICC < 0.979
```

```
icc(cbind(df.pa.milk.prerare$pa.B1, df.pa.milk.prerare$pa.B2), "twoway", "consistency")
```

```
##  Single Score Intraclass Correlation
## 
##    Model: twoway 
##    Type : consistency 
## 
##    Subjects = 13348 
##      Raters = 2 
##    ICC(C,1) = 0.978
## 
##  F-Test, H0: r0 = 0 ; H1: r0 > 0 
## F(13347,13347) = 90.3 , p = 0 
## 
##  95%-Confidence Interval for ICC Population Values:
##   0.977 < ICC < 0.979
```

## FIGURE 2B: Comparison of ASV prevalence between batches to identify potential batch-specific contaminants

1. Compare the prevalence of each ASV between Batch 1 and Batch 2

```
#Batch 1
physeq_milk.pa.B1 <- prune_samples(sample_data(physeq_milk.pa)$Batch =="Batch1", physeq_milk.pa) 
physeq_milk.pa.B1 #428 samples
```

```
## phyloseq-class experiment-level object
## otu_table()   OTU Table:         [ 13348 taxa and 428 samples ]
## sample_data() Sample Data:       [ 428 samples by 148 sample variables ]
## tax_table()   Taxonomy Table:    [ 13348 taxa by 7 taxonomic ranks ]
```

```
#Batch 2
physeq_milk.pa.B2 <- prune_samples(sample_data(physeq_milk.pa)$Batch =="Batch2", physeq_milk.pa) 
physeq_milk.pa.B2 #760 samples
```

```
## phyloseq-class experiment-level object
## otu_table()   OTU Table:         [ 13348 taxa and 760 samples ]
## sample_data() Sample Data:       [ 760 samples by 148 sample variables ]
## tax_table()   Taxonomy Table:    [ 13348 taxa by 7 taxonomic ranks ]
```

```
N1 <- 428 # Sample size of Batch 1
N2 <- 760 # Sample size of Batch 2

# Make data.frame of prevalence like before BUT keeping as proprotions instead of Percentages for now
df.pa.milk.prerare <- data.frame(pa.B1=taxa_sums(physeq_milk.pa.B1)/N1,   pa.B2=taxa_sums(physeq_milk.pa.B2)/N2, taxonomy=as(tax_table(physeq_milk.pa), "matrix"))
```

2. To make the sloped line in the prevalence plot, use the Structure\_comp function on all possible proportions in intervals of 0.001, given sample sizes N1 and N2 specified in the above chunk

```
# Generic use of the Structure_comp function on xs (all possible proportions in intervals of 0.001) given a specific value of N1 and N2 specified above
xs <- seq(0, 1, by = 0.001)  # All possible proportions in intervals of 0.001
ysmax <- Structure_comp(xs)   # ysmax is the MINUMUM a proportion can be in one batch, given a certain proportion in another batch (xs). In the case of extremely low values of xs, this can be negative (this happens more often with really low N, i.e. cannot actually say that the taxa is a contaminant, prevalence too low and/or N too low). 
ysmin <- rep(0, length(xs))
df2 <- data.frame(xs, ysmin, ysmax)
```

3. Asthetic modifications for plot

```
#remove the prefix p__ from the taxonmy names
df.pa.milk.prerare$Phylum <- gsub("p__", "", df.pa.milk.prerare$taxonomy.Phylum)
#Keep major milk phyla, merge everything else into Other.
df.pa.milk.prerare$Phylum <- ifelse(df.pa.milk.prerare$Phylum == "Proteobacteria" | df.pa.milk.prerare$Phylum == "Actinobacteria" | df.pa.milk.prerare$Phylum == "Firmicutes" | df.pa.milk.prerare$Phylum == "Bacteroidetes", df.pa.milk.prerare$Phylum, "Other")
#Reorder the phyla 
df.pa.milk.prerare$Phylum = factor(df.pa.milk.prerare$Phylum, levels=c('Proteobacteria','Firmicutes', "Actinobacteria", "Bacteroidetes", "Other"))

#Colour pallet for the figure (color by phylum)
cbPalette <- c("navy", "paleturquoise4",  "violetred3", "skyblue1", "goldenrod3")
```

4. Plot the ASV prevalence comparison between Batch 1 and Batch 2. Taxa BELOW the orange line will be defined as potential contaminants based on this prevalence comparison

```
Fig2B <- ggplot() + 
  geom_ribbon(aes(x=xs*100, ymin=ysmin*100, ymax=ysmax*100), data=df2, fill="gray80")+
  geom_ribbon(aes(y=xs*100, xmin=ysmin*100, xmax=ysmax*100), data=df2, fill="gray80")+
  geom_point(data=df.pa.milk.prerare, aes(x=pa.B1*100, y=pa.B2*100, color=Phylum), alpha=0.75, size=2) + 
  xlab("Batch 1 (n=428)") + 
  ylab("Batch 2 (n=760)") + 
  scale_color_manual(values = cbPalette) +
  theme_minimal()+ 
  theme(legend.position = "right")+
  geom_line(data = df2, aes(x = xs*100, y = ysmax*100), colour="#e6800b") + 
  geom_line(data = df2, aes(x = ysmax*100, y = xs*100), colour="#e6800b") +
  scale_y_continuous(limits=c(0,100), expand = c(0,0))+
  scale_x_continuous(limits=c(0,101), expand = c(0,0))

Fig2B
```

5. Make lists of potential contaminants specific to Batch 1 and Batch 2 based on the prevalence comparison shown in Figure 2B

```
df.pa.milk.prerare$Formula_B1 <- Structure_comp(df.pa.milk.prerare$pa.B1) # Given prevalence in Batch1
df.pa.milk.prerare$Formula_B2 <- Structure_comp(df.pa.milk.prerare$pa.B2)# Given prevalence in Batch2

ASV_Discard_B1 <- subset(df.pa.milk.prerare, pa.B2 < Formula_B1) # ASVs below the "minimum prevalence" in Batch 2 (shaded area on x-axis)
dim(ASV_Discard_B1) # 623, Most of these are rare, but a few are quite prevalent - these are the potential contaminants that lead to large batch effects
```

```
## [1] 623  12
```

```
ASV_Discard_B2 <- subset(df.pa.milk.prerare, pa.B1 < Formula_B2) # ASVs below the "minimum prevalence" in Batch 1 (shaded area on y-axis)
dim(ASV_Discard_B2) # 37, most of these are rare
```

```
## [1] 37 12
```

6. Check the ICC between batches for the prevalence of ASVs

```
icc(cbind(df.pa.milk.prerare$pa.B1, df.pa.milk.prerare$pa.B2), "twoway", "agreement")
```

```
##  Single Score Intraclass Correlation
## 
##    Model: twoway 
##    Type : agreement 
## 
##    Subjects = 13348 
##      Raters = 2 
##    ICC(A,1) = 0.224
## 
##  F-Test, H0: r0 = 0 ; H1: r0 > 0 
## F(13347,8999) = 1.59 , p = 7.69e-122 
## 
##  95%-Confidence Interval for ICC Population Values:
##   0.206 < ICC < 0.241
```

```
icc(cbind(df.pa.milk.prerare$pa.B1, df.pa.milk.prerare$pa.B2), "twoway", "consistency")
```

```
##  Single Score Intraclass Correlation
## 
##    Model: twoway 
##    Type : consistency 
## 
##    Subjects = 13348 
##      Raters = 2 
##    ICC(C,1) = 0.226
## 
##  F-Test, H0: r0 = 0 ; H1: r0 > 0 
## F(13347,13347) = 1.59 , p = 4.26e-155 
## 
##  95%-Confidence Interval for ICC Population Values:
##   0.21 < ICC < 0.242
```

7. ICC between batches for the prevalence of ASVs identified as contaminants by between-batch comparisons vs. those not identified as contaminants by this comparison

```
df.pa.milk.prerare$Discard <-ifelse(row.names(df.pa.milk.prerare) %in% c(rownames(ASV_Discard_B1), rownames(ASV_Discard_B2)), "Remove", "Keep")

table(df.pa.milk.prerare$Discard)
```

```
## 
##   Keep Remove 
##  12688    660
```

```
# Split, assess those identified as potential contaminants seporately from those not identified as potential contaminants by the between-batch comparison
df.milk.prerare_true <- subset(df.pa.milk.prerare, Discard=="Keep")
df.milk.prerare_Contam <- subset(df.pa.milk.prerare, Discard=="Remove")


# Check ICC (intraclass correlation) for 'true signals'
icc(cbind(df.milk.prerare_true$pa.B1, df.milk.prerare_true$pa.B2), "twoway", "agreement")
```

```
##  Single Score Intraclass Correlation
## 
##    Model: twoway 
##    Type : agreement 
## 
##    Subjects = 12688 
##      Raters = 2 
##    ICC(A,1) = 0.8
## 
##  F-Test, H0: r0 = 0 ; H1: r0 > 0 
## F(12687,5145) = 9.1 , p = 0 
## 
##  95%-Confidence Interval for ICC Population Values:
##   0.792 < ICC < 0.808
```

```
icc(cbind(df.milk.prerare_true$pa.B1, df.milk.prerare_true$pa.B2), "twoway", "consistency")
```

```
##  Single Score Intraclass Correlation
## 
##    Model: twoway 
##    Type : consistency 
## 
##    Subjects = 12688 
##      Raters = 2 
##    ICC(C,1) = 0.802
## 
##  F-Test, H0: r0 = 0 ; H1: r0 > 0 
## F(12687,12687) = 9.1 , p = 0 
## 
##  95%-Confidence Interval for ICC Population Values:
##   0.796 < ICC < 0.808
```

```
 # ICC for potential contaminants
icc(cbind(df.milk.prerare_Contam$pa.B1, df.milk.prerare_Contam$pa.B2), "twoway", "agreement")
```

```
##  Single Score Intraclass Correlation
## 
##    Model: twoway 
##    Type : agreement 
## 
##    Subjects = 660 
##      Raters = 2 
##    ICC(A,1) = 0.0117
## 
##  F-Test, H0: r0 = 0 ; H1: r0 > 0 
##  F(659,650) = 1.03 , p = 0.352 
## 
##  95%-Confidence Interval for ICC Population Values:
##   -0.048 < ICC < 0.073
```

```
icc(cbind(df.milk.prerare_Contam$pa.B1, df.milk.prerare_Contam$pa.B2), "twoway", "consistency")
```

```
##  Single Score Intraclass Correlation
## 
##    Model: twoway 
##    Type : consistency 
## 
##    Subjects = 660 
##      Raters = 2 
##    ICC(C,1) = 0.0149
## 
##  F-Test, H0: r0 = 0 ; H1: r0 > 0 
##  F(659,659) = 1.03 , p = 0.351 
## 
##  95%-Confidence Interval for ICC Population Values:
##   -0.061 < ICC < 0.091
```

## FIGURE 2C: Comparison of ASV average relative abundance between Batch 1 and Batch 2 samples

1. Plot comparison of average relative abundance (%) between batches

```
m <- prune_samples(sample_sums(physeq_sample2)>0, physeq_sample2) 

m_relative  = transform_sample_counts(m, function(x) x*100 / sum(x) ) # plot easier to interpret when in relative abundances

# Make phyloseq object of presence-absence in Batch1 vs. Batch2
physeq_milk.B1 <- prune_samples(sample_data(m_relative)$Batch =="Batch1", m_relative) #428 samples
physeq_milk.B2 <- prune_samples(sample_data(m_relative)$Batch =="Batch2", m_relative) #760 samples 

# Make data.frame of abundances, with additional taxonomy info attached
df.milk.prerare <- data.frame(pa.B1=taxa_sums(physeq_milk.B1)/428, pa.B2=taxa_sums(physeq_milk.B2)/760,
                      taxonomy=as(tax_table(m_relative), "matrix"))


df.milk.prerare$Discard <- ifelse(rownames(df.milk.prerare) %in% c(rownames(ASV_Discard_B1), rownames(ASV_Discard_B2)), "Remove", "Keep")
sum(df.milk.prerare$Discard == "Remove")
```

```
## [1] 660
```

```
#Color only by 4 main phyla, make other = black
df.milk.prerare$Phylum <- gsub("p__", "", df.milk.prerare$taxonomy.Phylum)

df.milk.prerare$Phylum <- ifelse(df.milk.prerare$Phylum == "Proteobacteria" | df.milk.prerare$Phylum == "Actinobacteria" | df.milk.prerare$Phylum == "Firmicutes" | df.milk.prerare$Phylum == "Bacteroidetes", df.milk.prerare$Phylum, "Other")

df.milk.prerare$Phylum = factor(df.milk.prerare$Phylum, levels=c('Proteobacteria','Firmicutes', "Actinobacteria", "Bacteroidetes", "Other"))

Fig2C <- ggplot(data=df.milk.prerare, aes(x=pa.B1, y=pa.B2, color=Phylum, shape = Discard)) + 
  geom_point(aes(x=pa.B1, y=pa.B2), alpha=0.75, size=2) + 
  xlab("Batch 1 (n=428)") + 
  ylab("Batch 2 (n=760)") + 
  scale_color_manual(values = cbPalette) + 
  theme_minimal()+
  scale_y_continuous(expand = c(0.01,0.01), limits = c(0,21.5))+
  scale_x_continuous(expand = c(0.01,0.01), limits = c(0,21.5))+
  theme(legend.position = "right")+
  geom_smooth(aes(group=Discard), method='lm', size=0.5, color="black", linetype="dashed", fullrange=TRUE) +
  annotate("segment", x = 0, xend = 21, y = 0, yend = 21, colour = "red", size=0.25)

Fig2C
```

Important Note: The most abundant potential contaminants are seen in Batch 1, they take up sequencing space in Batch 1. Once these more abundant contaminant ASVs are removed, the data will be re-relativize to the new total read count and average relative abundance of the remaining milk microbiota of Batch 1 will increase (and become more comparable to Batch 2).

2. ICC of ASV average relative abundance between batches overall, after decontam (Agreement of ASVs after decontam is shown in Table S2)

```
icc(cbind(df.milk.prerare$pa.B1, df.milk.prerare$pa.B2), "twoway", "agreement")
```

```
##  Single Score Intraclass Correlation
## 
##    Model: twoway 
##    Type : agreement 
## 
##    Subjects = 13348 
##      Raters = 2 
##    ICC(A,1) = 0.583
## 
##  F-Test, H0: r0 = 0 ; H1: r0 > 0 
## F(13347,13347) = 3.8 , p = 0 
## 
##  95%-Confidence Interval for ICC Population Values:
##   0.572 < ICC < 0.595
```

```
icc(cbind(df.milk.prerare$pa.B1, df.milk.prerare$pa.B2), "twoway", "consistency")
```

```
##  Single Score Intraclass Correlation
## 
##    Model: twoway 
##    Type : consistency 
## 
##    Subjects = 13348 
##      Raters = 2 
##    ICC(C,1) = 0.583
## 
##  F-Test, H0: r0 = 0 ; H1: r0 > 0 
## F(13347,13347) = 3.8 , p = 0 
## 
##  95%-Confidence Interval for ICC Population Values:
##   0.572 < ICC < 0.594
```

3. ICC of contaminant and true signal ASVs separately, between batches after decontam (shown in Figure 2C)

```
# split, compare contaminants and 'true signals' seporately
df.milk.prerare_true <- subset(df.milk.prerare, Discard=="Keep")
df.milk.prerare_Contam <- subset(df.milk.prerare, Discard=="Remove")

# ICC (intraclass correlation) of 'true signals'

icc(cbind(df.milk.prerare_true$pa.B1, df.milk.prerare_true$pa.B2), "twoway", "agreement")
```

```
##  Single Score Intraclass Correlation
## 
##    Model: twoway 
##    Type : agreement 
## 
##    Subjects = 12688 
##      Raters = 2 
##    ICC(A,1) = 0.66
## 
##  F-Test, H0: r0 = 0 ; H1: r0 > 0 
## F(12687,12663) = 4.89 , p = 0 
## 
##  95%-Confidence Interval for ICC Population Values:
##   0.65 < ICC < 0.67
```

```
icc(cbind(df.milk.prerare_true$pa.B1, df.milk.prerare_true$pa.B2), "twoway", "consistency")
```

```
##  Single Score Intraclass Correlation
## 
##    Model: twoway 
##    Type : consistency 
## 
##    Subjects = 12688 
##      Raters = 2 
##    ICC(C,1) = 0.66
## 
##  F-Test, H0: r0 = 0 ; H1: r0 > 0 
## F(12687,12687) = 4.89 , p = 0 
## 
##  95%-Confidence Interval for ICC Population Values:
##   0.651 < ICC < 0.67
```

```
 # ICC (intraclass correlation) of 'potential contaminants'
icc(cbind(df.milk.prerare_Contam$pa.B1, df.milk.prerare_Contam$pa.B2), "twoway", "agreement")
```

```
##  Single Score Intraclass Correlation
## 
##    Model: twoway 
##    Type : agreement 
## 
##    Subjects = 660 
##      Raters = 2 
##    ICC(A,1) = 0.00319
## 
##  F-Test, H0: r0 = 0 ; H1: r0 > 0 
##  F(659,659) = 1.01 , p = 0.466 
## 
##  95%-Confidence Interval for ICC Population Values:
##   -0.071 < ICC < 0.077
```

```
icc(cbind(df.milk.prerare_Contam$pa.B1, df.milk.prerare_Contam$pa.B2), "twoway", "consistency")
```

```
##  Single Score Intraclass Correlation
## 
##    Model: twoway 
##    Type : consistency 
## 
##    Subjects = 660 
##      Raters = 2 
##    ICC(C,1) = 0.00328
## 
##  F-Test, H0: r0 = 0 ; H1: r0 > 0 
##  F(659,659) = 1.01 , p = 0.466 
## 
##  95%-Confidence Interval for ICC Population Values:
##   -0.073 < ICC < 0.08
```

# Removal of potential reagent contaminants identified by assessing the data strucutre and related data checks

1. Compare ASVs identified as potential contaminants by between-batch comparisons and the various between-run comparisons. Overall 769 unique potential contaminants were found

```
# First compare contaminants found in runs vs. those found by comparing batches, any crossover? (cannot be crossover in contaminant ASVs between the pairs of lists made from the same plot so merge those first)
Between_batch_ASV_Discard <- c(rownames(ASV_Discard_B1), rownames(ASV_Discard_B2)) # 660
Between_Runs_B1_Discard <- c(rownames(ASV_Discard_R1_B1), rownames(ASV_Discard_R2_B1)) # 198
Between_Run1_2_B2_Discard <- c(rownames(ASV_Discard_R1_B2), rownames(ASV_Discard_R2_B2)) # 33
Between_Run1_3_B2_Discard <- c(rownames(ASV_Discard_R1vR3_B2), rownames(ASV_Discard_R3vR1_B2)) # 21
Between_Run2_3_B2_Discard <- c(rownames(ASV_Discard_R2vR3_B2), rownames(ASV_Discard_R3vR2_B2)) #32

# Combine all contaminants into single vector
All_contaminants <- c(Between_batch_ASV_Discard, Between_Runs_B1_Discard, Between_Run1_2_B2_Discard, Between_Run1_3_B2_Discard, Between_Run2_3_B2_Discard)

sum(duplicated(All_contaminants)==FALSE) # There are 769 unqiue contaminants
```

```
## [1] 769
```

```
# EXTRA CHECKS

length(intersect(rownames(ASV_Discard_B1), Between_Runs_B1_Discard)) # 144 ASVs found as contaminants in batch 1 by comparing batches AND by comparing runs within Batch 1
```

```
## [1] 144
```

```
length(intersect(rownames(ASV_Discard_B2), Between_Runs_B1_Discard)) # 0 ASVs found as batch 2 contaminants by comparing batches AND by comparing runs within Batch 1
```

```
## [1] 0
```

```
# batch 2 contaminants
Batch2_Runs <- c(Between_Run1_2_B2_Discard, Between_Run1_3_B2_Discard, Between_Run2_3_B2_Discard)
sum(duplicated(Batch2_Runs)==FALSE) # 66 unqiue contaminants WITHIN batch 2
```

```
## [1] 66
```

```
length(intersect(rownames(ASV_Discard_B2), Batch2_Runs)) # 4 ASVs found as batch 2 contaminants by comparing batches AND by comparing runs within Batch 2
```

```
## [1] 4
```

```
length(intersect(rownames(ASV_Discard_B1), Batch2_Runs)) # 5 ASVs found as batch 1 contaminants by comparing batches AND by comparing runs within Batch 2, are these abundant ASVs?
```

```
## [1] 5
```

```
Batch1btw_And_Batch2in <- intersect(rownames(ASV_Discard_B1), Batch2_Runs) # REMEMBER object for later, will check to see if these ASVs are abundant for Table S2


length(intersect(Between_Runs_B1_Discard, Batch2_Runs)) # 2 "Within batch 1" contaminants also in "within batch 2"
```

```
## [1] 2
```

```
# ALL unqiue ASVs identified from between-run comparisons overall (batch 1 and 2)
All_between_run_discard <- c(Between_Runs_B1_Discard, Between_Run1_2_B2_Discard, Between_Run1_3_B2_Discard, Between_Run2_3_B2_Discard) # will be some duplicates
sum(duplicated(All_between_run_discard)==FALSE) # 262
```

```
## [1] 262
```

```
# OVERALL, which ASVs were identified as contaminants by between-batch comparisons AND Batch 1 between-run vs. batch 2 between-run comparisons?
length(intersect(Between_batch_ASV_Discard, Between_Runs_B1_Discard)) # 144
```

```
## [1] 144
```

```
length(intersect(Between_batch_ASV_Discard, Batch2_Runs)) # 9
```

```
## [1] 9
```

2. Remove potential reagent contaminants identified by the between-run comparisons and between-batch comparison fo prevalence

```
All_ASVs <- taxa_names(physeq_sample2) #13,348
Keep_ASVs <- All_ASVs[!(All_ASVs %in% All_contaminants)] 
length(Keep_ASVs) #12,579 ASVs to keep (13,348-12,579 = 769 unqiue contaminants)
```

```
## [1] 12579
```

```
data_Milk_filtered <- prune_taxa(Keep_ASVs, physeq_sample2)
data_Milk_filtered <- prune_samples(sample_sums(data_Milk_filtered)>0, data_Milk_filtered)
data_Milk_filtered
```

```
## phyloseq-class experiment-level object
## otu_table()   OTU Table:         [ 12579 taxa and 1188 samples ]
## sample_data() Sample Data:       [ 1188 samples by 148 sample variables ]
## tax_table()   Taxonomy Table:    [ 12579 taxa by 7 taxonomic ranks ]
```

```
# Still 1188 samples remaining with > 0 Reads 

data_prune <- data_Milk_filtered # Just renaming for convenience
```

3. Check the proportion of reads remaining after removing contaminants

```
summary(sample_sums(data_prune))/summary(sample_sums(physeq_sample2)) # The Inf is the sample with 0 reads, 0/0
```

```
##    Min. 1st Qu.  Median    Mean 3rd Qu.    Max. 
##     Inf  0.5815  0.5303  0.8086  0.8184  0.9999
```

## FIGURE 2D: Comparison of ASV average relative abundance between Batch 1 and Batch 2 samples after the potential contaminats identified using the data structure are removed

1. Plot comparison of average relative abundance (%) between batches Note that contaminating ASVs were removed before the data is re-relativized

```
m <- prune_samples(sample_sums(data_prune)>0, data_prune) 

m_relative  = transform_sample_counts(m, function(x) x*100 / sum(x) ) # plot easier to interpret when in relative abundances

# Make phyloseq object of presence-absence in Batch1 vs. Batch2
physeq_milk.B1 <- prune_samples(sample_data(m_relative)$Batch =="Batch1", m_relative) #428 samples
physeq_milk.B2 <- prune_samples(sample_data(m_relative)$Batch =="Batch2", m_relative) #760 samples 

# Make data.frame of prevalence, with additional taxonomy info attached!
df.milk.prerare <- data.frame(pa.B1=taxa_sums(physeq_milk.B1)/428, pa.B2=taxa_sums(physeq_milk.B2)/760,
                      taxonomy=as(tax_table(m_relative), "matrix"))

#Color only by 4 main phyla, make other = black
df.milk.prerare$Phylum <- gsub("p__", "", df.milk.prerare$taxonomy.Phylum)

df.milk.prerare$Phylum <- ifelse(df.milk.prerare$Phylum == "Proteobacteria" | df.milk.prerare$Phylum == "Actinobacteria" | df.milk.prerare$Phylum == "Firmicutes" | df.milk.prerare$Phylum == "Bacteroidetes", df.milk.prerare$Phylum, "Other")

df.milk.prerare$Phylum = factor(df.milk.prerare$Phylum, levels=c('Proteobacteria','Firmicutes', "Actinobacteria", "Bacteroidetes", "Other"))

Fig2D <- ggplot(data=df.milk.prerare, aes(x=pa.B1, y=pa.B2, color=Phylum)) + 
  geom_point(aes(x=pa.B1, y=pa.B2), alpha=0.75, size=2) + 
  xlab("Batch 1 (n=428)") + 
  ylab("Batch 2 (n=760)") + 
  theme(legend.position="right") + 
  scale_color_manual(values = cbPalette) + 
  theme_minimal()+
  scale_y_continuous(expand = c(0.01,0.01), limits = c(0,22))+
  scale_x_continuous(expand = c(0.01,0.01), limits = c(0,22))+
  theme(legend.position = "right")+
  geom_smooth(method='lm', size=0.5, color="black", linetype="dashed", fullrange=TRUE) +
  annotate("segment", x = 0, xend = 21, y = 0, yend = 21, colour = "red", size=0.25)

Fig2D
```

2. ICC of ASV average relative abundance between batches after all contaminant removal steps (decontam + data structure)

```
icc(cbind(df.milk.prerare$pa.B1, df.milk.prerare$pa.B2), "twoway", "agreement")              
icc(cbind(df.milk.prerare$pa.B1, df.milk.prerare$pa.B2), "twoway", "consistency")
```

## For Table S2: Extra checks of between-run and between-batch contaminants identified, including the total number of reads contributed by contaminants identified at each step and their average prevalence within different data subsets

1. Get total reads in samples overall for the 1188 samples with reads after decontam

```
m <- prune_samples(sample_sums(physeq_sample2)>0, physeq_sample2) 

total_reads <- sample_sums(m)

sdf_contam <- as(sample_data(m), "data.frame") # sample data, to be used in following chunks
```

2. BATCH 1 contaminants identified by BETWEEN-RUN comparisons: Identify the average percent of total reads identified as this type of contaminant/ASV and the Average prevalence of this type of contaminant

```
# phyloseq obj only containing potential contaminants identified within batch 1 by between-run comparisons
ps_contam <- prune_taxa(Between_Runs_B1_Discard, m) 
ps_contam
```

```
## phyloseq-class experiment-level object
## otu_table()   OTU Table:         [ 198 taxa and 1188 samples ]
## sample_data() Sample Data:       [ 1188 samples by 148 sample variables ]
## tax_table()   Taxonomy Table:    [ 198 taxa by 7 taxonomic ranks ]
```

```
# Total reads contributed by contaminants in samples
B1_run_cont_reads <- sample_sums(ps_contam)

# sample data
sdf_contam3 <- data.frame(sdf_contam, B1run_contam = B1_run_cont_reads, total_postDecon = total_reads) 


sdf_contam3$Pct_Contam_B1run <- sdf_contam3$B1run_contam*100/sdf_contam3$total_postDecon
```

Average Percent of total reads from Batch 1 between-run contaminant ASVs (Overall and stratified by Run)

```
summary(sdf_contam3$Pct_Contam_B1run)
```

```
##     Min.  1st Qu.   Median     Mean  3rd Qu.     Max. 
##  0.00000  0.00000  0.00000  0.20871  0.01791 26.66752
```

```
sd(sdf_contam3$Pct_Contam_B1run)
```

```
## [1] 1.396646
```

```
# Average Percent of total reads stratified by batch and run
data_summary_B1Run <- data_summary(sdf_contam3, varname = "Pct_Contam_B1run", groupnames = c("Batch", "Run"))
data_summary_B1Run
```

```
##    Batch   Run Pct_Contam_B1run        sd      median min       max        IQR
## 1 Batch1    R1        0.3667900 1.7125263 0.005811842   0 14.148086 0.05981554
## 2 Batch1    R2        0.1854531 0.8590812 0.008296001   0  7.461113 0.03398628
## 3 Batch2 Run52        0.1524041 1.2291258 0.000000000   0 18.345100 0.00000000
## 4 Batch2 Run53        0.2373827 1.9123017 0.000000000   0 26.667517 0.00000000
## 5 Batch2 Run54        0.1220497 0.9352498 0.000000000   0 14.150133 0.00000000
##   Q1.25%     Q3.75%        sem N not 0 % N not 0
## 1      0 0.05981554 0.11679333     111  51.62791
## 2      0 0.03398628 0.05886328     116  54.46009
## 3      0 0.00000000 0.07742765      54  21.42857
## 4      0 0.00000000 0.12022538      60  23.71542
## 5      0 0.00000000 0.05856761      52  20.39216
```

Average prevalence of a Batch 1 between-run contaminant ASV (Overall and stratified by Run)

```
## Prevalence is just the % of N not 0, so using data_summary again, but for this another groupname will be the ASVID
ps_contam_otu <- as.data.frame(t(as(otu_table(ps_contam), "matrix")))

ps_contam_otu_meta <- merge(sdf_contam, ps_contam_otu, by="row.names")

ps_contam_otu_meta_melt <- melt(ps_contam_otu_meta, measure.vars = colnames(ps_contam_otu), id.vars = c("Run")) # variable = ASVid

## Overall average prevalence/etc. of a contaminant ASV
data_summary_decontam_byASV <- data_summary(ps_contam_otu_meta_melt, varname = "value", groupnames = c("variable"))
summary(data_summary_decontam_byASV$`% N not 0`)
```

```
##    Min. 1st Qu.  Median    Mean 3rd Qu.    Max. 
##  0.3367  0.4209  0.5051  0.5518  0.5892  2.6094
```

```
sd(summary(data_summary_decontam_byASV$`% N not 0`))
```

```
## [1] 0.873826
```

```
# Prevalence is % of N not 0
data_summary_decontam_runStrat <- data_summary(ps_contam_otu_meta_melt, varname = "value", groupnames = c("variable", "Run"))
##colnames(data_summary_decontam_runStrat)[13]
colnames(data_summary_decontam_runStrat)[13] <- "Prevalence"

## To get the average prevalence ( % of N not 0) for these ASVs per group for each Run now, do another data_summary
av_prev_decontam_runStrat <- data_summary(data_summary_decontam_runStrat, varname = "Prevalence", groupnames = c("Run"))
av_prev_decontam_runStrat # column "Prevaelnce", shows the average prevaelnce of a contaminant identified by decontam in percent
```

```
##     Run Prevalence        sd   median min      max       IQR   Q1.25%   Q3.75%
## 1    R1  2.1869861 1.1514828 2.325581   0 6.511628 0.9302326 1.860465 2.790698
## 2    R2  0.3604116 0.9444995 0.000000   0 7.981221 0.0000000 0.000000 0.000000
## 3 Run52  0.1443001 0.4539658 0.000000   0 4.365079 0.0000000 0.000000 0.000000
## 4 Run53  0.1457260 0.4003265 0.000000   0 2.766798 0.0000000 0.000000 0.000000
## 5 Run54  0.1386413 0.4267169 0.000000   0 2.745098 0.0000000 0.000000 0.000000
##          sem N not 0 % N not 0
## 1 0.08183232     167  84.34343
## 2 0.06712266      31  15.65657
## 3 0.03226194      33  16.66667
## 4 0.02844996      37  18.68687
## 5 0.03032545      28  14.14141
```

3. BATCH 2 contaminants identified by BETWEEN-RUN comparisons: Identify the average percent of total reads identified as this type of contaminant/ASV and the Average prevalence of this type of contaminant

```
# phyloseq obj only containing potential contaminants identified within batch 1
ps_contam <- prune_taxa(Batch2_Runs, m) 
ps_contam
```

```
## phyloseq-class experiment-level object
## otu_table()   OTU Table:         [ 66 taxa and 1188 samples ]
## sample_data() Sample Data:       [ 1188 samples by 148 sample variables ]
## tax_table()   Taxonomy Table:    [ 66 taxa by 7 taxonomic ranks ]
```

```
# Total reads contributed by contaminants in samples
B2_run_cont_reads <- sample_sums(ps_contam)

# sample data
sdf_contam4 <- data.frame(sdf_contam3, B2run_contam = B2_run_cont_reads)  # adding to sample data that batch 1 contaminant read counts were already added to in the previous chunk

sdf_contam4$Pct_Contam_B2run <- sdf_contam4$B2run_contam*100/sdf_contam4$total_postDecon
```

Average Percent of total reads from Batch 2 between-run contaminant ASVs (Overall and stratified by Run)

```
# Average Percent of total reads identified as contaminants
summary(sdf_contam4$Pct_Contam_B2run)
```

```
##     Min.  1st Qu.   Median     Mean  3rd Qu.     Max. 
##  0.00000  0.00000  0.05485  1.27408  2.23159 59.39537
```

```
sd(sdf_contam4$Pct_Contam_B2run)
```

```
## [1] 2.871543
```

```
# Average % of original reads stratified by batch and run
data_summary_B2Run <- data_summary(sdf_contam4, varname = "Pct_Contam_B2run", groupnames = c("Batch", "Run"))
data_summary_B2Run
```

```
##    Batch   Run Pct_Contam_B2run       sd   median min       max        IQR
## 1 Batch1    R1        2.6607714 1.656585 2.868444   0  7.014738 2.67539484
## 2 Batch1    R2        3.0683728 1.633320 3.260551   0  6.547990 2.30478677
## 3 Batch2 Run52        0.2162231 1.179078 0.000000   0 10.993786 0.03668757
## 4 Batch2 Run53        0.4737897 4.106714 0.000000   0 59.395368 0.05534647
## 5 Batch2 Run54        0.4455758 3.020712 0.000000   0 42.387555 0.04264466
##     Q1.25%     Q3.75%        sem N not 0 % N not 0
## 1 1.179745 3.85513986 0.11297814     213  99.06977
## 2 1.954157 4.25894378 0.11191327     211  99.06103
## 3 0.000000 0.03668757 0.07427492      90  35.71429
## 4 0.000000 0.05534647 0.25818690     118  46.64032
## 5 0.000000 0.04264466 0.18916431      87  34.11765
```

Average prevalence of a Batch 2 between-run contaminant ASV (Overall and stratified by Run)

```
## Prevalence is just the % of N not 0, so using data_summary again, but for this another groupname will be the ASVID
ps_contam_otu <- as.data.frame(t(as(otu_table(ps_contam), "matrix")))


ps_contam_otu_meta <- merge(sdf_contam, ps_contam_otu, by="row.names")

ps_contam_otu_meta_melt <- melt(ps_contam_otu_meta, measure.vars = colnames(ps_contam_otu), id.vars = c("Run")) # variable = ASVid

## Overall average prevalence/etc. of a contaminant ASV
data_summary_decontam_byASV <- data_summary(ps_contam_otu_meta_melt, varname = "value", groupnames = c("variable"))
summary(data_summary_decontam_byASV$`% N not 0`)
```

```
##    Min. 1st Qu.  Median    Mean 3rd Qu.    Max. 
##  0.3367  0.5892  0.9259  2.8594  1.4310 36.1111
```

```
sd(summary(data_summary_decontam_byASV$`% N not 0`))
```

```
## [1] 14.26882
```

```
# Prevalence is % of N not 0
data_summary_decontam_runStrat <- data_summary(ps_contam_otu_meta_melt, varname = "value", groupnames = c("variable", "Run"))
##colnames(data_summary_decontam_runStrat)[13]
colnames(data_summary_decontam_runStrat)[13] <- "Prevalence"

## To get the average prevalence ( % of N not 0) for these ASVs per group for each Run now, do another data_summary
av_prev_decontam_runStrat <- data_summary(data_summary_decontam_runStrat, varname = "Prevalence", groupnames = c("Run"))
av_prev_decontam_runStrat # column "Prevaelnce", shows the average prevaelnce of a contaminant identified by decontam in percent
```

```
##     Run Prevalence         sd    median min       max      IQR    Q1.25%
## 1    R1  6.3424947 19.8545506 0.9302326   0 99.069767 2.093023 0.1162791
## 2    R2  6.3807085 20.1693882 0.9389671   0 99.061033 1.877934 0.0000000
## 3 Run52  0.9319384  1.0026720 0.7936508   0  3.968254 1.587302 0.0000000
## 4 Run53  1.0719847  1.0324867 1.1857708   0  3.952569 1.581028 0.0000000
## 5 Run54  0.6595365  0.7787407 0.3921569   0  3.137255 1.470588 0.0000000
##     Q3.75%        sem N not 0 % N not 0
## 1 2.209302 2.44392624      49  74.24242
## 2 1.877934 2.48268007      47  71.21212
## 3 1.587302 0.12342040      41  62.12121
## 4 1.581028 0.12709032      42  63.63636
## 5 1.470588 0.09585635      37  56.06061
```

4. Extra check for Table S2 (corresponds to a footnote 2 of Table S2). Repeat of Step 3 above but just for 5 ASVs identified as Batch 2 contaminatns by between-run comparisons AND Batch 1 'between-batch' contaminants

```
## phyloseq-class experiment-level object
## otu_table()   OTU Table:         [ 5 taxa and 1188 samples ]
## sample_data() Sample Data:       [ 1188 samples by 148 sample variables ]
## tax_table()   Taxonomy Table:    [ 5 taxa by 7 taxonomic ranks ]
```

Average Percent of total reads from ASVs identified as contaminant by both Batch 2 "between-run" AND Batch 1 'between-batch' comparisons (Overall and stratified by Run)

```
##    Min. 1st Qu.  Median    Mean 3rd Qu.    Max. 
##   0.000   0.000   0.000   1.010   1.874   7.015
```

```
## [1] 1.66407
```

```
##    Batch   Run Pct_Contam_B2runB1         sd   median min       max      IQR
## 1 Batch1    R1        2.597703873 1.64807576 2.820180   0 7.0147378 2.704557
## 2 Batch1    R2        2.991350027 1.62348220 3.171848   0 6.4975523 2.334702
## 3 Batch2 Run52        0.001788103 0.01041846 0.000000   0 0.1058167 0.000000
## 4 Batch2 Run53        0.003304709 0.02478547 0.000000   0 0.2785515 0.000000
## 5 Batch2 Run54        0.011920734 0.08912752 0.000000   0 1.0935601 0.000000
##     Q1.25%   Q3.75%         sem N not 0 % N not 0
## 1 1.135222 3.839779 0.112397834     213 99.069767
## 2 1.800864 4.135566 0.111239167     211 99.061033
## 3 0.000000 0.000000 0.000656301      11  4.365079
## 4 0.000000 0.000000 0.001558249       6  2.371542
## 5 0.000000 0.000000 0.005581382      14  5.490196
```

Average prevalence of a contaminant by both Batch 2 "between-run" AND Batch 1 'between-batch' comparisons (Overall and stratified by Run)

```
##    Min. 1st Qu.  Median    Mean 3rd Qu.    Max. 
##   5.976  12.963  31.987  24.512  35.522  36.111
```

```
## [1] 12.55845
```

```
##     Run Prevalence         sd     median      min       max        IQR
## 1    R1 65.9534884 38.9719151 85.5813953 15.34884 99.069767 63.7209302
## 2    R2 66.9483568 39.5992065 88.7323944 15.02347 99.061033 64.3192488
## 3 Run52  0.9523810  0.6640159  0.7936508  0.00000  1.587302  0.7936508
## 4 Run53  0.5533597  0.7704976  0.0000000  0.00000  1.581028  1.1857708
## 5 Run54  1.1764706  1.3298686  0.3921569  0.00000  3.137255  1.5686275
##       Q1.25%    Q3.75%        sem N not 0 % N not 0
## 1 33.0232558 96.744186 17.4287703       5       100
## 2 33.8028169 98.122066 17.7093035       5       100
## 3  0.7936508  1.587302  0.2969569       4        80
## 4  0.0000000  1.185771  0.3445770       2        40
## 5  0.3921569  1.960784  0.5947353       4        80
```

These 5 very prevalent and relatively abundant Batch 1 contaminants explain the unexpectedly high prevalence of some batch 2 within-run contaminants in batch 1.

5. BATCH 1 contaminants identified by BETWEEN-BATCH comparisons: Identify the average percent of total reads identified as this type of contaminant/ASV and the Average prevalence of this type of contaminant

```
## phyloseq-class experiment-level object
## otu_table()   OTU Table:         [ 623 taxa and 1188 samples ]
## sample_data() Sample Data:       [ 1188 samples by 148 sample variables ]
## tax_table()   Taxonomy Table:    [ 623 taxa by 7 taxonomic ranks ]
```

Average percent of total reads from Batch 1 specific contaminant ASVs (Overall and stratified by Run)

```
##     Min.  1st Qu.   Median     Mean  3rd Qu.     Max. 
##  0.00000  0.00000  0.08075 18.60815 35.28253 94.52183
```

```
## [1] 29.80408
```

```
##    Batch   Run Pct_Contam_B1Batch         sd   median         min       max
## 1 Batch1    R1         51.0112725 28.8988652 56.44556 0.017111567 94.521826
## 2 Batch1    R2         51.6116046 27.4335040 54.80020 0.006180406 94.068587
## 3 Batch2 Run52          0.1187181  0.4535184  0.00000 0.000000000  5.084746
## 4 Batch2 Run53          0.1807253  0.8155861  0.00000 0.000000000 10.869565
## 5 Batch2 Run54          0.2750816  1.2368047  0.00000 0.000000000 12.345679
##           IQR   Q1.25%      Q3.75%        sem N not 0 % N not 0
## 1 51.38094391 24.30668 75.68761899 1.97088625     215 100.00000
## 2 43.36373325 31.19266 74.55639380 1.87971272     213 100.00000
## 3  0.03560864  0.00000  0.03560864 0.02856897     100  39.68254
## 4  0.06747259  0.00000  0.06747259 0.05127546     120  47.43083
## 5  0.06889367  0.00000  0.06889367 0.07745172     125  49.01961
```

Average prevalence of a Batch 1 specific contaminant ASV (Overall and stratified by Run)

```
##    Min. 1st Qu.  Median    Mean 3rd Qu.    Max. 
##  0.3367  0.4209  0.6734  5.6246  4.2929 38.4680
```

```
## [1] 14.94646
```

```
##     Run Prevalence         sd   median min        max      IQR    Q1.25%
## 1    R1 15.3727276 27.2639109 2.790698   0 100.000000 10.46512 1.8604651
## 2    R2 15.3859487 28.4942381 1.877934   0  99.530516 10.79812 0.4694836
## 3 Run52  0.1248439  0.4683254 0.000000   0   5.158730  0.00000 0.0000000
## 4 Run53  0.1325982  0.5171730 0.000000   0   4.347826  0.00000 0.0000000
## 5 Run54  0.1359645  0.5222449 0.000000   0   4.705882  0.00000 0.0000000
##     Q3.75%        sem N not 0 % N not 0
## 1 12.32558 1.09230553     609  97.75281
## 2 11.26761 1.14159754     493  79.13323
## 3  0.00000 0.01876306      77  12.35955
## 4  0.00000 0.02072010      73  11.71750
## 5  0.00000 0.02092330      74  11.87801
```

6. BATCH 2 contaminants identified by BETWEEN-BATCH comparisons: Identify the average percent of total reads identified as this type of contaminant/ASV and the Average prevalence of this type of contaminant

```
## phyloseq-class experiment-level object
## otu_table()   OTU Table:         [ 37 taxa and 1188 samples ]
## sample_data() Sample Data:       [ 1188 samples by 148 sample variables ]
## tax_table()   Taxonomy Table:    [ 37 taxa by 7 taxonomic ranks ]
```

Average percent of total reads from Batch 2 specific contaminant ASVs (Overall and stratified by Run)

```
##     Min.  1st Qu.   Median     Mean  3rd Qu.     Max. 
##  0.00000  0.00000  0.00000  0.22891  0.01772 23.73869
```

```
## [1] 1.374073
```

```
##    Batch   Run Pct_Contam_B2Batch       sd      median min      max        IQR
## 1 Batch1    R1          0.0000000 0.000000 0.000000000   0  0.00000 0.00000000
## 2 Batch1    R2          0.0000000 0.000000 0.000000000   0  0.00000 0.00000000
## 3 Batch2 Run52          0.5437690 1.700111 0.007983434   0 12.46846 0.15918378
## 4 Batch2 Run53          0.2704932 1.712059 0.000000000   0 23.32892 0.04846005
## 5 Batch2 Run54          0.2607169 1.694027 0.000000000   0 23.73869 0.06006557
##   Q1.25%     Q3.75%       sem N not 0 % N not 0
## 1      0 0.00000000 0.0000000       0   0.00000
## 2      0 0.00000000 0.0000000       0   0.00000
## 3      0 0.15918378 0.1070969     137  54.36508
## 4      0 0.04846005 0.1076362     126  49.80237
## 5      0 0.06006557 0.1060841     113  44.31373
```

Average prevalence of a Batch 2 contaminant ASV (Overall and stratified by Run)

```
##    Min. 1st Qu.  Median    Mean 3rd Qu.    Max. 
##  0.5051  0.5051  0.5892  1.2854  0.8418 17.0875
```

```
## [1] 6.6783
```

```
##     Run Prevalence       sd   median min      max       IQR    Q1.25%   Q3.75%
## 1    R1   0.000000 0.000000 0.000000   0  0.00000 0.0000000 0.0000000 0.000000
## 2    R2   0.000000 0.000000 0.000000   0  0.00000 0.0000000 0.0000000 0.000000
## 3 Run52   2.359502 6.115451 1.190476   0 36.90476 1.1904762 0.3968254 1.587302
## 4 Run53   2.008332 4.349202 1.185771   0 26.48221 0.7905138 0.7905138 1.581028
## 5 Run54   1.664017 2.776671 1.176471   0 16.86275 1.1764706 0.3921569 1.568627
##         sem N not 0 % N not 0
## 1 0.0000000       0   0.00000
## 2 0.0000000       0   0.00000
## 3 1.0053740      35  94.59459
## 4 0.7150043      35  94.59459
## 5 0.4564819      36  97.29730
```

7. ALL UNIQUE contaminants identified using the DATA STRUCTURE (all between-run comparisons and the between-batch comparison): Identify the average percent of total reads identified as this type of contaminant/ASV and the Average prevalence of this type of contaminant

```
## phyloseq-class experiment-level object
## otu_table()   OTU Table:         [ 769 taxa and 1188 samples ]
## sample_data() Sample Data:       [ 1188 samples by 148 sample variables ]
## tax_table()   Taxonomy Table:    [ 769 taxa by 7 taxonomic ranks ]
```

Average percent of total reads from all ASVs identified as a potential contaminants based on the data structure (Overall and stratified by Run)

```
##    Min. 1st Qu.  Median    Mean 3rd Qu.    Max. 
##  0.0000  0.0429  0.5818 19.2357 35.4477 94.5218
```

```
## [1] 29.65654
```

```
##    Batch   Run Pct_Contam_Struc        sd      median         min      max
## 1 Batch1    R1        51.195361 28.826353 56.45553959 0.017111567 94.52183
## 2 Batch1    R2        51.865575 27.262071 54.81152592 0.006180406 94.07990
## 3 Batch2 Run52         1.014591  2.576282  0.09173633 0.000000000 18.49454
## 4 Batch2 Run53         1.043892  4.593406  0.07601865 0.000000000 59.55371
## 5 Batch2 Run54         1.089668  3.897706  0.11300712 0.000000000 42.41571
##          IQR      Q1.25%     Q3.75%       sem N not 0 % N not 0
## 1 51.4047789 24.32714093 75.7319198 1.9659409     215 100.00000
## 2 43.2043443 31.42960717 74.6339514 1.8679663     213 100.00000
## 3  0.5950880  0.00000000  0.5950880 0.1622905     185  73.41270
## 4  0.4212363  0.01413787  0.4353741 0.2887849     210  83.00395
## 5  0.4631225  0.01259787  0.4757204 0.2440838     204  80.00000
```

Average prevalence of an ASV identified as a potential contamiant based on the data structure overall (Overall and stratified by Run)

```
##    Min. 1st Qu.  Median    Mean 3rd Qu.    Max. 
##  0.3367  0.4209  0.6734  4.7521  2.3569 38.4680
```

```
## [1] 15.10217
```

```
##     Run Prevalence        sd   median min       max      IQR    Q1.25%   Q3.75%
## 1    R1 12.6881785 25.167787 2.325581   0 100.00000 5.581395 0.9302326 6.511628
## 2    R2 12.6174472 26.284289 1.408451   0  99.53052 6.572770 0.0000000 6.572770
## 3 Run52  0.3183892  1.519048 0.000000   0  36.90476 0.000000 0.0000000 0.000000
## 4 Run53  0.3197007  1.190983 0.000000   0  26.48221 0.000000 0.0000000 0.000000
## 5 Run54  0.2702772  0.879935 0.000000   0  16.86275 0.000000 0.0000000 0.000000
##          sem N not 0 % N not 0
## 1 0.90757363     690  89.72692
## 2 0.94783571     550  71.52146
## 3 0.05477827     178  23.14694
## 4 0.04294795     181  23.53706
## 5 0.03173127     167  21.71651
```

8. The percentage of the total contaminant reads (decontam + data structure) that were identified in each step (uses sdf\_contam8, made in previous chunks, contains the number of reads contributed by potential contaminants identified by each dataset comparison, and sdf\_contam2, made in a previous chunk, contained the number of reads identified as contaminant by decontam)

```
## For these 1,188 samples that still had reads after contaminant removal, add decon_contam (the reads in these samples that were identified as contaminants by decontam)
sdf_contam2_sub <- subset(sdf_contam2, select = c("decon_contam"))

sdf_contam9 <- merge(sdf_contam8, sdf_contam2_sub, by="row.names")
dim(sdf_contam9) # 1,188 samples
```

```
## [1] 1188  163
```

```
## sdf_contam9$All_str_contam # These are the number of total reads that were identified as contaminants using the data structure from the above chunk, the 769 unique ASVs identified through one or more of the between-run and/or between-batch comparisons. 

sdf_contam9$Total_contam_reads <- (sdf_contam9$All_str_contam + sdf_contam9$decon_contam) # total reads, those identified using the data structure plus decontam

# total contaminant reads identified by decontam on average
# NOTE that all the 0/0 will be NA for all of these
summary(sdf_contam9$decon_contam*100/sdf_contam9$Total_contam_reads)
```

```
##     Min.  1st Qu.   Median     Mean  3rd Qu.     Max.     NA's 
##   0.0000   0.0000   0.1193  18.1943  23.9048 100.0000      101
```

```
sd(sdf_contam9$decon_contam*100/sdf_contam9$Total_contam_reads, na.rm=TRUE)
```

```
## [1] 31.19411
```

```
# total contaminant reads identified by data structure (overall, on average)
summary(sdf_contam9$All_str_contam*100/sdf_contam9$Total_contam_reads)
```

```
##    Min. 1st Qu.  Median    Mean 3rd Qu.    Max.    NA's 
##    0.00   76.10   99.88   81.81  100.00  100.00     101
```

```
sd(sdf_contam9$All_str_contam*100/sdf_contam9$Total_contam_reads, na.rm=TRUE)
```

```
## [1] 31.19411
```

```
# total contaminant reads identified by between-batch comparisons, batch 1 
summary(sdf_contam9$B1batch_contam*100/sdf_contam9$Total_contam_reads)
```

```
##    Min. 1st Qu.  Median    Mean 3rd Qu.    Max.    NA's 
##    0.00    0.00   52.94   51.84   99.84  100.00     101
```

```
sd(sdf_contam9$B1batch_contam*100/sdf_contam9$Total_contam_reads, na.rm=TRUE)
```

```
## [1] 44.97061
```

```
# total contaminant reads identified by between-batch comparisons, batch 2
summary(sdf_contam9$B2batch_contam*100/sdf_contam9$Total_contam_reads)
```

```
##    Min. 1st Qu.  Median    Mean 3rd Qu.    Max.    NA's 
##    0.00    0.00    0.00   14.59   14.72  100.00     101
```

```
sd(sdf_contam9$B2batch_contam*100/sdf_contam9$Total_contam_reads, na.rm=TRUE)
```

```
## [1] 28.20276
```

```
# total contaminant reads identified by between-run comparisons, batch 1
summary(sdf_contam9$B1run_contam*100/sdf_contam9$Total_contam_reads)
```

```
##      Min.   1st Qu.    Median      Mean   3rd Qu.      Max.      NA's 
##   0.00000   0.00000   0.00000   6.12687   0.09879 100.00000       101
```

```
sd(sdf_contam9$B1run_contam*100/sdf_contam9$Total_contam_reads, na.rm=TRUE)
```

```
## [1] 19.31472
```

```
# total contaminant reads identified by between-run comparisons, batch 2
summary(sdf_contam9$B2run_contam*100/sdf_contam9$Total_contam_reads)
```

```
##    Min. 1st Qu.  Median    Mean 3rd Qu.    Max.    NA's 
##   0.000   0.000   5.034  12.981   7.284 100.000     101
```

```
sd(sdf_contam9$B2run_contam*100/sdf_contam9$Total_contam_reads, na.rm=TRUE)
```

```
## [1] 23.95139
```

```
# Based on The above chunks looking at prevalence of contaminants identified by within-run comparions seen in Table S2, weighted average for prevalence of these within-run ASVs for legend of Figure S1
weight_av <- (0.6*(215+213) + 2.9*(252+253+255))/1188
weight_av
```

```
## [1] 2.07138
```

# Additional verification of this contaminant removal methods performance, assessing contaminant removal by between-batch comparisons in different scenarios (Figure S2)

Replicating Figure 2B (Batch comparison) with sub-samples of Batch 1 and Batch 2 to obtain: 1- A low sample size for both (N=25 in each) 2- An intermediate sample size for both (n=60 in each) 3- An imbalanced sample size N=25 in Batch 1 and N=60 in Batch 2 N=60 in Batch 1 and N=25 in Batch 2 4 - A more imbalanced sample size, but using all available samples in the selected subset

1. These sub-samples will only be taken from a homogenous subset of primiparous mothers that directly breastfed, and who's child did not have an asthma diagnosis at 5 years. This will allow for more consistency among milk samples that are compared between batches

```
physeq_similar <- subset_samples(physeq_sample2, BM_mode_3m== "DBM only" & older_sibs=="No" & asthma_5y == "No")

physeq_similar2 <- prune_taxa(taxa_sums(physeq_similar)>0, physeq_similar) # non 0 taxa only
physeq_similar2 # much fewer taxa, perhaps an indication of much similar sample types in the phyloseq object
```

```
## phyloseq-class experiment-level object
## otu_table()   OTU Table:         [ 4604 taxa and 171 samples ]
## sample_data() Sample Data:       [ 171 samples by 148 sample variables ]
## tax_table()   Taxonomy Table:    [ 4604 taxa by 7 taxonomic ranks ]
```

```
table(sample_data(physeq_similar2)$Batch)
```

```
## 
## Batch1 Batch2 
##     63    108
```

2. Make the binary dataset used for assessment of data strucutre, and make the random sub-samples used

```
m <- prune_samples(sample_sums(physeq_similar2)>0, physeq_similar2) #1188 samples [2 samples with 0 reads from batch2]
 
# Convert the otu table in the phyloseq object to presence-absence table 
physeq_milk.pa <- transform_sample_counts(m, function(abund) 1*(abund>0))

physeq_milk.pa.B1 <- prune_samples(sample_data(physeq_milk.pa)$Batch =="Batch1", physeq_milk.pa) 
physeq_milk.pa.B1 # 63
```

```
## phyloseq-class experiment-level object
## otu_table()   OTU Table:         [ 4604 taxa and 63 samples ]
## sample_data() Sample Data:       [ 63 samples by 148 sample variables ]
## tax_table()   Taxonomy Table:    [ 4604 taxa by 7 taxonomic ranks ]
```

```
physeq_milk.pa.B2 <- prune_samples(sample_data(physeq_milk.pa)$Batch =="Batch2", physeq_milk.pa) 
physeq_milk.pa.B2 # 108
```

```
## phyloseq-class experiment-level object
## otu_table()   OTU Table:         [ 4604 taxa and 108 samples ]
## sample_data() Sample Data:       [ 108 samples by 148 sample variables ]
## tax_table()   Taxonomy Table:    [ 4604 taxa by 7 taxonomic ranks ]
```

```
# Assign vector of random numbers to each batch that is equal in length to sample size
set.seed(123)
sample_data(physeq_milk.pa.B1)$B1_list <- sample(seq(from = 1, to = 63, by = 1), size = 63, replace = FALSE) # will not be any duplicates this way


sample_data(physeq_milk.pa.B2)$B2_list <- sample(seq(from = 1, to = 108, by = 1), size = 108, replace = FALSE) # will not be any duplicates this way

# Now because they were randomly assigned (not in order from 1 to 108), selecting the numbers 1-25 will select 25 random rows/samples
## N=20 for each batch
Milk.pa.B1_20 <- subset_samples(physeq_milk.pa.B1, B1_list <= 25)
Milk.pa.B1_20
```

```
## phyloseq-class experiment-level object
## otu_table()   OTU Table:         [ 4604 taxa and 25 samples ]
## sample_data() Sample Data:       [ 25 samples by 149 sample variables ]
## tax_table()   Taxonomy Table:    [ 4604 taxa by 7 taxonomic ranks ]
```

```
Milk.pa.B2_20 <- subset_samples(physeq_milk.pa.B2, B2_list <= 25)
Milk.pa.B2_20
```

```
## phyloseq-class experiment-level object
## otu_table()   OTU Table:         [ 4604 taxa and 25 samples ]
## sample_data() Sample Data:       [ 25 samples by 149 sample variables ]
## tax_table()   Taxonomy Table:    [ 4604 taxa by 7 taxonomic ranks ]
```

```
# Also, because the sample assigned a value of 1 will always be the same, and 2, etc., when we increase the sample size to for example 60, we are just adding another 35 samples to the dataset that had n=25 samples

## N=60 for each batch

Milk.pa.B1_60 <- subset_samples(physeq_milk.pa.B1, B1_list <= 60) # really just selecting almost all samples in this specific subset, this is okay. The purpose of this is mostly to compare the slope lines with different sample sizes which is not influenced by the prevalences of ASVs.
Milk.pa.B1_60
```

```
## phyloseq-class experiment-level object
## otu_table()   OTU Table:         [ 4604 taxa and 60 samples ]
## sample_data() Sample Data:       [ 60 samples by 149 sample variables ]
## tax_table()   Taxonomy Table:    [ 4604 taxa by 7 taxonomic ranks ]
```

```
Milk.pa.B2_60 <- subset_samples(physeq_milk.pa.B2, B2_list <= 60)
Milk.pa.B2_60
```

```
## phyloseq-class experiment-level object
## otu_table()   OTU Table:         [ 4604 taxa and 60 samples ]
## sample_data() Sample Data:       [ 60 samples by 149 sample variables ]
## tax_table()   Taxonomy Table:    [ 4604 taxa by 7 taxonomic ranks ]
```

## FIGURE S2A: Using all available samples from the Homogenous subset of samples (Batch 1 n=63 and Batch 2 n=108)

1. Compare the prevalence of each ASV between Batch 1 and Batch 2 for the homogenous subset

```
N1 <- 63 # Sample size of Batch 1
N2 <- 108 # Sample size of Batch 2

# Make data.frame of prevalence like before BUT keeping as proprotions instead of Percentages for now
df.pa.milk.prerare <- data.frame(pa.B1=taxa_sums(physeq_milk.pa.B1)/N1,   pa.B2=taxa_sums(physeq_milk.pa.B2)/N2, taxonomy=as(tax_table(physeq_milk.pa), "matrix"))

sum(df.pa.milk.prerare$pa.B1 >0 | df.pa.milk.prerare$pa.B2 >0) # 4,604
```

```
## [1] 4604
```

2. To make the sloped line in the prevalence plot, use the Structure\_comp function on all possible proportions in intervals of 0.001, given sample sizes N1 and N2 specified in the above chunk

```
# Generic use of the Structure_comp function on xs (all possible proportions in intervals of 0.001) given a specific value of N1 and N2 specified above
xs <- seq(0, 1, by = 0.001)  # All possible proportions in intervals of 0.001
ysmax <- Structure_comp(xs)   # ysmax is the MINUMUM a proportion can be in one batch, given a certain proportion in another batch (xs). In the case of extremely low values of xs, this can be negative (this happens more often with really low N, i.e. cannot actually say that the taxa is a contaminant, prevalence too low and/or N too low). 
ysmin <- rep(0, length(xs))
df2 <- data.frame(xs, ysmin, ysmax)
```

3. Asthetic modifications for plot

```
#remove the prefix p__ from the taxonmy names
df.pa.milk.prerare$Phylum <- gsub("p__", "", df.pa.milk.prerare$taxonomy.Phylum)
#Keep major milk phyla, merge everything else into Other.
df.pa.milk.prerare$Phylum <- ifelse(df.pa.milk.prerare$Phylum == "Proteobacteria" | df.pa.milk.prerare$Phylum == "Actinobacteria" | df.pa.milk.prerare$Phylum == "Firmicutes" | df.pa.milk.prerare$Phylum == "Bacteroidetes", df.pa.milk.prerare$Phylum, "Other")
#Reorder the phyla 
df.pa.milk.prerare$Phylum = factor(df.pa.milk.prerare$Phylum, levels=c('Proteobacteria','Firmicutes', "Actinobacteria", "Bacteroidetes", "Other"))
 
#Colour pallet for the figure (color by phylum)
cbPalette <- c("navy", "paleturquoise4",  "violetred3", "skyblue1", "goldenrod3")
```

4. Plot the ASV prevalence comparison between Batch 1 and Batch 2 for the homogenous subset

```
FigS2A <- ggplot() + 
  geom_ribbon(aes(x=xs*100, ymin=ysmin*100, ymax=ysmax*100), data=df2, fill="gray80")+
  geom_ribbon(aes(y=xs*100, xmin=ysmin*100, xmax=ysmax*100), data=df2, fill="gray80")+
  geom_point(data=df.pa.milk.prerare, aes(x=pa.B1*100, y=pa.B2*100, color=Phylum), alpha=0.75, size=2) + 
  xlab("Batch 1 (n=63)") + 
  ylab("Batch 2 (n=108)") + 
  scale_color_manual(values = cbPalette) +
  theme_minimal()+ 
  theme(legend.position = "right")+
  geom_line(data = df2, aes(x = xs*100, y = ysmax*100), colour="#e6800b") + 
  geom_line(data = df2, aes(x = ysmax*100, y = xs*100), colour="#e6800b") +
  scale_y_continuous(limits=c(0,100), expand = c(0,0))+
  scale_x_continuous(limits=c(0,101), expand = c(0,0))

FigS2A
```

5. Make lists of potential contaminants specific to Batch 1 and Batch 2 based on the prevalence comparison shown in Figure S2A

```
df.pa.milk.prerare$Formula_B1 <- Structure_comp(df.pa.milk.prerare$pa.B1) # Given prevalence in Batch1
df.pa.milk.prerare$Formula_B2 <- Structure_comp(df.pa.milk.prerare$pa.B2)# Given prevalence in Batch2
 
ASV_Discard_B1_63_unev <- subset(df.pa.milk.prerare, pa.B2 < Formula_B1) # ASVs below the "minimum prevalence" in Batch 2 (shaded area on x-axis)
dim(ASV_Discard_B1_63_unev) # 378
```

```
## [1] 378  12
```

```
ASV_Discard_B2_100_unev <- subset(df.pa.milk.prerare, pa.B1 < Formula_B2) # ASVs below the "minimum prevalence" in Batch 1 (shaded area on y-axis)
dim(ASV_Discard_B2_100_unev) # 8
```

```
## [1]  8 12
```

## FIGURE S2B: A low sample size in both batches (N=25 each)

1. Compare the prevalence of each ASV between Batch 1 and Batch 2 for the low sample size subset

```
N1 <- 25 # Sample size of Batch 1
N2 <- 25 # Sample size of Batch 2

# Make data.frame of prevalence like before BUT keeping as proprotions instead of Percentages for now
df.pa.milk.prerare <- data.frame(pa.B1=taxa_sums(Milk.pa.B1_20)/N1,   pa.B2=taxa_sums(Milk.pa.B2_20)/N2, taxonomy=as(tax_table(physeq_milk.pa), "matrix"))
dim(df.pa.milk.prerare) # 4604
```

```
## [1] 4604    9
```

```
## N(ASV), number of non-zero taxa (taxa that are At Least present in 1 of the 2 batches) 
sum(df.pa.milk.prerare$pa.B1 >0 | df.pa.milk.prerare$pa.B2 >0) # 1,832
```

```
## [1] 1832
```

2. To make the sloped line in the prevalence plot, use the Structure\_comp function on all possible proportions in intervals of 0.001, given sample sizes N1 and N2 specified in the above chunk

```
# Generic use of the Structure_comp function on xs (all possible proportions in intervals of 0.001) given a specific value of N1 and N2 specified above
xs <- seq(0, 1, by = 0.001)  # All possible proportions in intervals of 0.001
ysmax <- Structure_comp(xs)   # ysmax is the MINUMUM a proportion can be in one batch, given a certain proportion in another batch (xs). In the case of extremely low values of xs, this can be negative (this happens more often with really low N, i.e. cannot actually say that the taxa is a contaminant, prevalence too low and/or N too low). 
ysmin <- rep(0, length(xs))
df2 <- data.frame(xs, ysmin, ysmax)
```

3. Asthetic modifications for plot

```
#remove the prefix p__ from the taxonmy names
df.pa.milk.prerare$Phylum <- gsub("p__", "", df.pa.milk.prerare$taxonomy.Phylum)
#Keep major milk phyla, merge everything else into Other.
df.pa.milk.prerare$Phylum <- ifelse(df.pa.milk.prerare$Phylum == "Proteobacteria" | df.pa.milk.prerare$Phylum == "Actinobacteria" | df.pa.milk.prerare$Phylum == "Firmicutes" | df.pa.milk.prerare$Phylum == "Bacteroidetes", df.pa.milk.prerare$Phylum, "Other")
#Reorder the phyla 
df.pa.milk.prerare$Phylum = factor(df.pa.milk.prerare$Phylum, levels=c('Proteobacteria','Firmicutes', "Actinobacteria", "Bacteroidetes", "Other"))

#Colour pallet for the figure (color by phylum)
cbPalette <- c("navy", "paleturquoise4",  "violetred3", "skyblue1", "goldenrod3")
```

4. Plot the ASV prevalence comparison between Batch 1 and Batch 2 for the low sample size subset

```
FigS2B <- ggplot() + 
  geom_ribbon(aes(x=xs*100, ymin=ysmin*100, ymax=ysmax*100), data=df2, fill="gray80")+
  geom_ribbon(aes(y=xs*100, xmin=ysmin*100, xmax=ysmax*100), data=df2, fill="gray80")+
  geom_point(data=df.pa.milk.prerare, aes(x=pa.B1*100, y=pa.B2*100, color=Phylum), alpha=0.75, size=2) + 
  xlab("Batch 1 (n=25)") + 
  ylab("Batch 2 (n=25)") + 
  scale_color_manual(values = cbPalette) +
  theme_minimal()+ 
  theme(legend.position = "right")+
  geom_line(data = df2, aes(x = xs*100, y = ysmax*100), colour="#e6800b") + 
  geom_line(data = df2, aes(x = ysmax*100, y = xs*100), colour="#e6800b") +
  scale_y_continuous(limits=c(0,100), expand = c(0,0))+
  scale_x_continuous(limits=c(0,101), expand = c(0,0))

FigS2B
```

5. Make lists of potential contaminants specific to Batch 1 and Batch 2 based on the prevalence comparison shown in Figure S2B

```
df.pa.milk.prerare$Formula_B1 <- Structure_comp(df.pa.milk.prerare$pa.B1) # Given prevalence in Batch1
df.pa.milk.prerare$Formula_B2 <- Structure_comp(df.pa.milk.prerare$pa.B2)# Given prevalence in Batch2

ASV_Discard_B1_25 <- subset(df.pa.milk.prerare, pa.B2 < Formula_B1) # ASVs below the "minimum prevalence" in Run 2 (shaded area on x-axis)
dim(ASV_Discard_B1_25) # 161
```

```
## [1] 161  12
```

```
ASV_Discard_B2_25 <- subset(df.pa.milk.prerare, pa.B1 < Formula_B2) # ASVs below the "minimum prevalence" in Run 1 (shaded area on y-axis)
dim(ASV_Discard_B2_25) # 1
```

```
## [1]  1 12
```

## FIGURE S2C: An imbalanced sample size, with more samples in the Batch 2 (the batch that had fewer contaminants identified in it)

1. Compare the prevalence of each ASV between Batch 1 and Batch 2 for an imbalanced subset (more samples in Batch 2)

```
N1 <- 25 # Sample size of Batch 1
N2 <- 60 # Sample size of Batch 2

# Make data.frame of prevalence like before BUT keeping as proprotions instead of Percentages for now
df.pa.milk.prerare <- data.frame(pa.B1=taxa_sums(Milk.pa.B1_20)/N1,   pa.B2=taxa_sums(Milk.pa.B2_60)/N2, taxonomy=as(tax_table(physeq_milk.pa), "matrix"))

sum(df.pa.milk.prerare$pa.B1 >0 | df.pa.milk.prerare$pa.B2 >0) # 3,344
```

```
## [1] 2229
```

2. To make the sloped line in the prevalence plot, use the Structure\_comp function on all possible proportions in intervals of 0.001, given sample sizes N1 and N2 specified in the above chunk

```
# Generic use of the Structure_comp function on xs (all possible proportions in intervals of 0.001) given a specific value of N1 and N2 specified above
xs <- seq(0, 1, by = 0.001)  # All possible proportions in intervals of 0.001
ysmax <- Structure_comp(xs)   # ysmax is the MINUMUM a proportion can be in one batch, given a certain proportion in another batch (xs). In the case of extremely low values of xs, this can be negative (this happens more often with really low N, i.e. cannot actually say that the taxa is a contaminant, prevalence too low and/or N too low). 
ysmin <- rep(0, length(xs))
df2 <- data.frame(xs, ysmin, ysmax)
```

3. Asthetic modifications for plot

```
#remove the prefix p__ from the taxonmy names
df.pa.milk.prerare$Phylum <- gsub("p__", "", df.pa.milk.prerare$taxonomy.Phylum)
#Keep major milk phyla, merge everything else into Other.
df.pa.milk.prerare$Phylum <- ifelse(df.pa.milk.prerare$Phylum == "Proteobacteria" | df.pa.milk.prerare$Phylum == "Actinobacteria" | df.pa.milk.prerare$Phylum == "Firmicutes" | df.pa.milk.prerare$Phylum == "Bacteroidetes", df.pa.milk.prerare$Phylum, "Other")
#Reorder the phyla 
df.pa.milk.prerare$Phylum = factor(df.pa.milk.prerare$Phylum, levels=c('Proteobacteria','Firmicutes', "Actinobacteria", "Bacteroidetes", "Other"))

#Colour pallet for the figure (color by phylum)
cbPalette <- c("navy", "paleturquoise4",  "violetred3", "skyblue1", "goldenrod3")
```

4. Plot the ASV prevalence comparison between Batch 1 and Batch 2 for an imbalanced subset (more samples in Batch 2)

```
FigS2C <- ggplot() + 
  geom_ribbon(aes(x=xs*100, ymin=ysmin*100, ymax=ysmax*100), data=df2, fill="gray80")+
  geom_ribbon(aes(y=xs*100, xmin=ysmin*100, xmax=ysmax*100), data=df2, fill="gray80")+
  geom_point(data=df.pa.milk.prerare, aes(x=pa.B1*100, y=pa.B2*100, color=Phylum), alpha=0.75, size=2) + 
  xlab("Batch 1 (n=25)") + 
  ylab("Batch 2 (n=60)") + 
  scale_color_manual(values = cbPalette) +
  theme_minimal()+ 
  theme(legend.position = "right")+
  geom_line(data = df2, aes(x = xs*100, y = ysmax*100), colour="#e6800b") + 
  geom_line(data = df2, aes(x = ysmax*100, y = xs*100), colour="#e6800b") +
  scale_y_continuous(limits=c(0,100), expand = c(0,0))+
  scale_x_continuous(limits=c(0,101), expand = c(0,0))

FigS2C
```

5. Make lists of potential contaminants specific to Batch 1 and Batch 2 based on the prevalence comparison shown in Figure S2C

```
df.pa.milk.prerare$Formula_B1 <- Structure_comp(df.pa.milk.prerare$pa.B1) # Given prevalence in Batch1
df.pa.milk.prerare$Formula_B2 <- Structure_comp(df.pa.milk.prerare$pa.B2)# Given prevalence in Batch2

ASV_Discard_B1_25_unev <- subset(df.pa.milk.prerare, pa.B2 < Formula_B1) # ASVs below the "minimum prevalence" in Run 2 (shaded area on x-axis)
dim(ASV_Discard_B1_25_unev) # 169
```

```
## [1] 169  12
```

```
ASV_Discard_B2_60_unev <- subset(df.pa.milk.prerare, pa.B1 < Formula_B2) # ASVs below the "minimum prevalence" in Run 1 (shaded area on y-axis)
dim(ASV_Discard_B2_60_unev) # 3
```

```
## [1]  3 12
```

## FIGURE S2D: An imbalanced sample size, with more samples in Batch 1 (the batch that had more contaminants identified in it)

1. Compare the prevalence of each ASV between Batch 1 and Batch 2 for an imbalanced subset (more samples in Batch 1)

```
N1 <- 60 # Sample size of Batch 1 
N2 <- 25 # Sample size of Batch 2

# Make data.frame of prevalence like before BUT keeping as proprotions instead of Percentages for now
df.pa.milk.prerare <- data.frame(pa.B1=taxa_sums(Milk.pa.B1_60)/N1,   pa.B2=taxa_sums(Milk.pa.B2_20)/N2, taxonomy=as(tax_table(physeq_milk.pa), "matrix"))

sum(df.pa.milk.prerare$pa.B1 >0 | df.pa.milk.prerare$pa.B2 >0) # 2,982
```

```
## [1] 2982
```

2. To make the sloped line in the prevalence plot, use the Structure\_comp function on all possible proportions in intervals of 0.001, given sample sizes N1 and N2 specified in the above chunk

```
# Generic use of the Structure_comp function on xs (all possible proportions in intervals of 0.001) given a specific value of N1 and N2 specified above
xs <- seq(0, 1, by = 0.001)  # All possible proportions in intervals of 0.001
ysmax <- Structure_comp(xs)   # ysmax is the MINUMUM a proportion can be in one batch, given a certain proportion in another batch (xs). In the case of extremely low values of xs, this can be negative (this happens more often with really low N, i.e. cannot actually say that the taxa is a contaminant, prevalence too low and/or N too low). 
ysmin <- rep(0, length(xs))
df2 <- data.frame(xs, ysmin, ysmax)
```

3. Asthetic modifications for plot

```
#remove the prefix p__ from the taxonmy names
df.pa.milk.prerare$Phylum <- gsub("p__", "", df.pa.milk.prerare$taxonomy.Phylum)
#Keep major milk phyla, merge everything else into Other.
df.pa.milk.prerare$Phylum <- ifelse(df.pa.milk.prerare$Phylum == "Proteobacteria" | df.pa.milk.prerare$Phylum == "Actinobacteria" | df.pa.milk.prerare$Phylum == "Firmicutes" | df.pa.milk.prerare$Phylum == "Bacteroidetes", df.pa.milk.prerare$Phylum, "Other")
#Reorder the phyla 
df.pa.milk.prerare$Phylum = factor(df.pa.milk.prerare$Phylum, levels=c('Proteobacteria','Firmicutes', "Actinobacteria", "Bacteroidetes", "Other"))

#Colour pallet for the figure (color by phylum)
cbPalette <- c("navy", "paleturquoise4",  "violetred3", "skyblue1", "goldenrod3")
```

4. Plot the ASV prevalence comparison between Batch 1 and Batch 2 for an imbalanced subset (more samples in Batch 1)

```
FigS2D <- ggplot() + 
  geom_ribbon(aes(x=xs*100, ymin=ysmin*100, ymax=ysmax*100), data=df2, fill="gray80")+
  geom_ribbon(aes(y=xs*100, xmin=ysmin*100, xmax=ysmax*100), data=df2, fill="gray80")+
  geom_point(data=df.pa.milk.prerare, aes(x=pa.B1*100, y=pa.B2*100, color=Phylum), alpha=0.75, size=2) + 
  xlab("Batch 1 (n=60)") + 
  ylab("Batch 2 (n=25)") + 
  scale_color_manual(values = cbPalette) +
  theme_minimal()+ 
  theme(legend.position = "right")+
  geom_line(data = df2, aes(x = xs*100, y = ysmax*100), colour="#e6800b") + 
  geom_line(data = df2, aes(x = ysmax*100, y = xs*100), colour="#e6800b") +
  scale_y_continuous(limits=c(0,100), expand = c(0,0))+
  scale_x_continuous(limits=c(0,101), expand = c(0,0))

FigS2D
```

5. Make lists of potential contaminants specific to Batch 1 and Batch 2 based on the prevalence comparison shown in Figure S2D

```
df.pa.milk.prerare$Formula_B1 <- Structure_comp(df.pa.milk.prerare$pa.B1) # Given prevalence in Batch1
df.pa.milk.prerare$Formula_B2 <- Structure_comp(df.pa.milk.prerare$pa.B2)# Given prevalence in Batch2

ASV_Discard_B1_60_unev <- subset(df.pa.milk.prerare, pa.B2 < Formula_B1) # ASVs below the "minimum prevalence" in Run 2 (shaded area on x-axis)
dim(ASV_Discard_B1_60_unev) # 194
```

```
## [1] 194  12
```

```
ASV_Discard_B2_25_unev <- subset(df.pa.milk.prerare, pa.B1 < Formula_B2) # ASVs below the "minimum prevalence" in Run 1 (shaded area on y-axis)
dim(ASV_Discard_B2_25_unev) # 3
```

```
## [1]  3 12
```

## Compare the between-batch contaminants identified in the overall dataset (Figure S2B) to the Homogenous subset (Figure S2A), small sample-size dataset (Figure S2B), and the small imbalanced datasets (Figure S2C & S2D)

1. Make a phyloseq object containing only contaminants identified by between-batch comparison of data structure assessment in the overall dataset (Figure S2B)

```
All_ASVs <- taxa_names(physeq_sample2) #13,348

# Another object with only contaminants identified by between-batch comparisons (these contaminants were generally much more abundant than the between-run contaminants)
Batch_discard <- All_ASVs[(All_ASVs %in% Between_batch_ASV_Discard)]  # removed the !
length(Batch_discard) # 660
```

```
## [1] 660
```

```
Batch_Contaminant <- prune_taxa(Batch_discard, physeq_sample2) # keeping the discarded ASVs for the contaminant object
Batch_Contaminant <- prune_samples(sample_sums(Batch_Contaminant)>0, Batch_Contaminant)
Batch_Contaminant
```

```
## phyloseq-class experiment-level object
## otu_table()   OTU Table:         [ 660 taxa and 951 samples ]
## sample_data() Sample Data:       [ 951 samples by 148 sample variables ]
## tax_table()   Taxonomy Table:    [ 660 taxa by 7 taxonomic ranks ]
```

2. Contaminant ASVs of the Homogenous subset (Figure S2A) vs. overall dataset

```
Homogenous_contams <- c(row.names(ASV_Discard_B1_63_unev), row.names(ASV_Discard_B2_100_unev))
length(Homogenous_contams) # 386 total contaminants identified in this subset
```

```
## [1] 386
```

```
length(intersect(Between_batch_ASV_Discard, Homogenous_contams)) # 323
```

```
## [1] 323
```

```
323*100/386 # 84% of ASVs identified as contaminants in the homogenous subset were ALSO identified as contaminants in the overall dataset (though this made up only ~50% of ASVs identified as contaminants overall, the sample size is substantially smaller, increasing variation in prevalence values.)
```

```
## [1] 83.67876
```

```
# Identify the proportion of total between-batch contaminants that were also identified as between-batch contaminants using the homogenous subset
Batch_Contaminant_homog <- prune_taxa(Homogenous_contams, Batch_Contaminant) 
Batch_Contaminant_homog  # 323
```

```
## phyloseq-class experiment-level object
## otu_table()   OTU Table:         [ 323 taxa and 951 samples ]
## sample_data() Sample Data:       [ 951 samples by 148 sample variables ]
## tax_table()   Taxonomy Table:    [ 323 taxa by 7 taxonomic ranks ]
```

```
sum(sample_sums(Batch_Contaminant)) #  9,458,355 total contaminant reads in 951 samples from 660 taxa
```

```
## [1] 9458355
```

```
sum(sample_sums(Batch_Contaminant_homog)) #  9,342,618 total contaminant reads in 951 samples from just 323 taxa
```

```
## [1] 9342618
```

```
sum(sample_sums(Batch_Contaminant_homog))*100/sum(sample_sums(Batch_Contaminant)) # 99%
```

```
## [1] 98.77635
```

3. Contaminant ASVs of the small sample-size subset (Figure S2B) vs. overall dataset

```
# contaminant ASVs of the small sample-size homogenous subset
SmallHom_contams <- c(row.names(ASV_Discard_B1_25), row.names(ASV_Discard_B2_25))
length(SmallHom_contams) # 162 total contaminants identified in this subset
```

```
## [1] 162
```

```
# Identify the proportion of total between-batch contaminants that were also identified as between-batch contaminants using the smaller homogenous subset
Batch_Contaminant_small <- prune_taxa(SmallHom_contams, Batch_Contaminant) 
Batch_Contaminant_small
```

```
## phyloseq-class experiment-level object
## otu_table()   OTU Table:         [ 147 taxa and 951 samples ]
## sample_data() Sample Data:       [ 951 samples by 148 sample variables ]
## tax_table()   Taxonomy Table:    [ 147 taxa by 7 taxonomic ranks ]
```

```
sum(sample_sums(Batch_Contaminant_small)) #  7,991,213 total contaminant reads in 951 samples from just 147 taxa
```

```
## [1] 7991213
```

```
sum(sample_sums(Batch_Contaminant_small))*100/sum(sample_sums(Batch_Contaminant)) # 84%
```

```
## [1] 84.4884
```

4. Contaminant ASVs of the small imbalanced subset with more samples in Batch 2 (Figure S2C) vs. overall dataset

```
# contaminant ASVs of the uneven homogenous subset - More samples in Batch 2
UnevB2_contams <- c(row.names(ASV_Discard_B1_25_unev), row.names(ASV_Discard_B2_60_unev))
length(UnevB2_contams) # 172 total contaminants identified in this subset
```

```
## [1] 172
```

```
# Identify the proportion of total between-batch contaminants that were also identified as between-batch contaminants using the smaller homogenous subset
UnevB2_Contaminant <- prune_taxa(UnevB2_contams, Batch_Contaminant) 
UnevB2_Contaminant
```

```
## phyloseq-class experiment-level object
## otu_table()   OTU Table:         [ 160 taxa and 951 samples ]
## sample_data() Sample Data:       [ 951 samples by 148 sample variables ]
## tax_table()   Taxonomy Table:    [ 160 taxa by 7 taxonomic ranks ]
```

```
sum(sample_sums(UnevB2_Contaminant)) #  8,485,687 total contaminant reads in 951 samples from just 160 taxa
```

```
## [1] 8485687
```

```
sum(sample_sums(UnevB2_Contaminant))*100/sum(sample_sums(Batch_Contaminant)) # 90%
```

```
## [1] 89.71631
```

5. Contaminant ASVs of the small imbalanced subset with more samples in Batch 1 (Figure S2D) vs. overall dataset

```
# contaminant ASVs of the uneven homogenous subset - More samples in Batch 1
UnevB1_contams <- c(row.names(ASV_Discard_B1_60_unev), row.names(ASV_Discard_B2_25_unev))
length(UnevB1_contams) # 197 total contaminants identified in this subset
```

```
## [1] 197
```

```
# Identify the proportion of total between-batch contaminants that were also identified as between-batch contaminants using the smaller homogenous subset
UnevB1_Contaminant <- prune_taxa(UnevB1_contams, Batch_Contaminant) 
UnevB1_Contaminant
```

```
## phyloseq-class experiment-level object
## otu_table()   OTU Table:         [ 171 taxa and 951 samples ]
## sample_data() Sample Data:       [ 951 samples by 148 sample variables ]
## tax_table()   Taxonomy Table:    [ 171 taxa by 7 taxonomic ranks ]
```

```
sum(sample_sums(UnevB1_Contaminant)) #  8,008,740 total contaminant reads in 951 samples from just 171 taxa
```

```
## [1] 8008740
```

```
sum(sample_sums(UnevB1_Contaminant))*100/sum(sample_sums(Batch_Contaminant)) # 85%
```

```
## [1] 84.67371
```

# Assess the within-batch consistency of the between-batch contaminant profile (in contrast to a lack of consistency for non-contaminants)

## FIGURE S3: Assessment of correlations among the most abundant contaminants and true-singals of Batch 1 and 2

The preprocessing steps performed for the final dataset are repeated specifically for this analysis of within-batch consistency. These include: 1- Rarefying to a depth of 8,000 reads per sample, 3- Removing rare taxa (ASVs present with a total count less than 60 reads across the dataset), 4- Relativized to the total reads per sample after rarfaction (8,000).

1. Data pre-processing
2. Modify dataset for assessment of correlations among the most abundant contaminants and true signals within Batch 1 and within Batch 2

```
data_Abund_Batch1 <- subset_samples(data_rarefy_rel_ext2, Batch == "Batch1")
data_Abund_Batch2 <- subset_samples(data_rarefy_rel_ext2, Batch == "Batch2")


# Taxa in at least abundance of 0.1% 
datafr1_B1 = filter_taxa(data_Abund_Batch1, function(x) mean(x) > 0.1, TRUE)  #  ~ 55% of ASVs are non-contaminants, 45% are contaminants
datafr1_B1 # Top ~ 100 taxa of Batch 1 >0.1% abundance
```

```
## phyloseq-class experiment-level object
## otu_table()   OTU Table:         [ 109 taxa and 422 samples ]
## sample_data() Sample Data:       [ 422 samples by 148 sample variables ]
## tax_table()   Taxonomy Table:    [ 109 taxa by 7 taxonomic ranks ]
```

```
datafr1_B2 = filter_taxa(data_Abund_Batch2, function(x) mean(x) > 0.1, TRUE) # ALL abundant taxa are non-contaminant ASVs for Batch 2
datafr1_B2 # Top ~ 80 taxa of Batch 2 >0.1% abundance
```

```
## phyloseq-class experiment-level object
## otu_table()   OTU Table:         [ 77 taxa and 534 samples ]
## sample_data() Sample Data:       [ 534 samples by 148 sample variables ]
## tax_table()   Taxonomy Table:    [ 77 taxa by 7 taxonomic ranks ]
```

```
datafr1_B2_contams = filter_taxa(data_Abund_Batch2, function(x) mean(x) > 0.1, TRUE) # Tried 0.01% too and only 4 contaminatns of Batch 2 are >0.01%, tried 0.001%, but taxa are too rare for spearman correlation
datafr1_B2_contams
```

```
## phyloseq-class experiment-level object
## otu_table()   OTU Table:         [ 77 taxa and 534 samples ]
## sample_data() Sample Data:       [ 534 samples by 148 sample variables ]
## tax_table()   Taxonomy Table:    [ 77 taxa by 7 taxonomic ranks ]
```

```
data_vegan_B1 <- veganotu(datafr1_B1)

data_vegan_B2 <- veganotu(datafr1_B2)

data_vegan_B2_contams <- veganotu(datafr1_B2_contams)


## Additional packages used for Figure S3
library(Hmisc)
library(ggcorrplot)
```

## FIGURE S3A: Spearman correlation heatmap for contaminants of Batch 1

```
data_vegan_contam <- subset(data_vegan_B1, select = intersect(colnames(data_vegan_B1), rownames(ASV_Discard_B1)))
dim(data_vegan_contam)
```

```
## [1] 422  51
```

```
#Correlate each ASV with each other ASV
phi_est2 <- rcorr(as.matrix(data_vegan_contam), type="spearman") 

#Created corrleation and P-value 'matrix'
table_R_phi <- (as.matrix(phi_est2$r))
dim(table_R_phi)
```

```
## [1] 51 51
```

```
row.names(table_R_phi) <- NULL
colnames(table_R_phi) <- NULL


# overall plot, ignore p-values, strength more important
FigS3A <- ggcorrplot(table_R_phi, lab=FALSE, show.legend = TRUE) 

FigS3A
```

Note two taxa consistently not associated with any other contaminants but associated with each other - may be a different type of contamination, also some of the rarer taxa will not show strong corrleations.

## FIGURE S3B: Spearman correlation heatmap for non-contaminants of Batch 1

```
data_vegan_Noncontam <- subset(data_vegan_B1, select = setdiff(colnames(data_vegan_B1), rownames(ASV_Discard_B1)))
dim(data_vegan_Noncontam) # 58 non-contaminants
```

```
## [1] 422  58
```

```
#Correlate each ASV with each other ASV
phi_est2 <- rcorr(as.matrix(data_vegan_Noncontam), type="spearman") 

#Created corrleation and P-value 'matrix'
table_R_phi <- (as.matrix(phi_est2$r))
dim(table_R_phi)
```

```
## [1] 58 58
```

```
row.names(table_R_phi) <- NULL
colnames(table_R_phi) <- NULL


# overall plot, ignore p-values, strength more important
FigS3B <- ggcorrplot(table_R_phi, lab=FALSE, show.legend = TRUE) 

FigS3B
```

NOTE - We were not able to do Spearman correlation heatmap for contaminants of Batch 2, contaminants of Batch 2 are extremely rare

```
data_vegan_contam <- subset(data_vegan_B2_contams, select = intersect(colnames(data_vegan_B2_contams), rownames(ASV_Discard_B2)))
dim(data_vegan_contam)
```

```
## [1] 534   1
```

```
 # Won't see any correlations
```

## FIGURE S3C: Spearman correlation heatmap for non-contaminants of Batch 2

```
data_vegan_Noncontam <- subset(data_vegan_B2, select = setdiff(colnames(data_vegan_B2), rownames(ASV_Discard_B2)))
dim(data_vegan_Noncontam) # All abundant taxa are non-contaminants
```

```
## [1] 534  76
```

```
#Correlate each ASV with each other ASV
phi_est2 <- rcorr(as.matrix(data_vegan_Noncontam), type="spearman") 

#Created corrleation and P-value 'matrix'
table_R_phi <- (as.matrix(phi_est2$r))
dim(table_R_phi)
```

```
## [1] 76 76
```

```
row.names(table_R_phi) <- NULL
colnames(table_R_phi) <- NULL


# overall plot, ignore p-values, strength more important
FigS3C <- ggcorrplot(table_R_phi, lab=FALSE, show.legend = TRUE) 

FigS3C
```

# Additional data pre-processing including rarefaction, removal of rare ASVs, and relativizing data

1. Rarefaction

```
#set seed for reproducibility 
set.seed(14507) 

#plot the figure (not included in the paper)
#rarecurve(t(otu_table(data_prune)), step = 50, cex=0.5, label = FALSE, xlim = c(1, 15000))
#abline(v = 8000, col="blue")

#Checking the number of samples and taxa lost by removing samples with <8000 reads/sample.
Check_data_prune <- prune_samples(sample_sums(data_prune)>=8000, data_prune)
Check_data_prune <- prune_taxa(taxa_sums(Check_data_prune)>0, Check_data_prune)
Check_data_prune  # 10661 taxa in samples with >8000 reads
```

```
## phyloseq-class experiment-level object
## otu_table()   OTU Table:         [ 10661 taxa and 870 samples ]
## sample_data() Sample Data:       [ 870 samples by 148 sample variables ]
## tax_table()   Taxonomy Table:    [ 10661 taxa by 7 taxonomic ranks ]
```

```
#Rarify to 8,000 reads/sample
set.seed(29132)
data_rarefy_pruned = rarefy_even_depth(data_prune, sample.size = 8000)
ntaxa(data_rarefy_pruned)
 
#9309

# few OTUs removed when compared with dataset of samples with >=8000 reads  - decontamination also removed most very rare / likely spurious ASVs previously removed by rarefaction (e.g. sequencing artifacts)
```

2. Remove rare ASVs that are more likely sequencing artifacts

```
#Remove taxa seen less than 60 times in total
data_prune3 = filter_taxa(data_rarefy_pruned, function(x) sum(x) > 60 , TRUE) 
ntaxa(data_prune3) # 908
```

```
## [1] 908
```

3. Assess the percent of reads remaining after removing rare ASVs - nearly all reads are kept, this filtering step is acceptable.

```
summary(sample_sums(data_rarefy_pruned)) # rarified
```

```
##    Min. 1st Qu.  Median    Mean 3rd Qu.    Max. 
##    8000    8000    8000    8000    8000    8000
```

```
summary(sample_sums(data_prune3)) # rarified + pruned
```

```
##    Min. 1st Qu.  Median    Mean 3rd Qu.    Max. 
##    3628    7916    7965    7924    7990    8000
```

```
# percent of reads remaining after pruning (compared to right before pruning)
summary(sample_sums(data_prune3)*100/sample_sums(data_rarefy_pruned))
```

```
##    Min. 1st Qu.  Median    Mean 3rd Qu.    Max. 
##   45.35   98.95   99.56   99.04   99.88  100.00
```

```
sd(sample_sums(data_prune3)*100/sample_sums(data_rarefy_pruned))
```

```
## [1] 2.456347
```

4. Assess the percent of ASVs per sample remaining on average
5. Relativize to the rarefaction threshold (8000 reads per sample)

```
data_rarefy_rel  = transform_sample_counts(data_prune3, function(x) x*100 / 8000)
```

# Assessment of batch variability in the overal milk microbiota composition

Batch variability was assessed prior to contaminant removal (Figure 2E), after decontam (Figure 2F), and after considering the data structure i.e. taxa prevalence between the batches (Figure 2G). Milk microbiota composition variability was assessed using the PCoA plot assessed on the Bray-Curtis dissimilarity.

## Figure 2E: Batch variability on the original prior to contaminant identification and removal using decontam and data structure comparison

The preprocessing steps performed above are repeated. These include: 1-Excluding reads unassigned at the kingdom level, and reads assigned to mitochondria, Chloroplast and Cyanobacteria taxonomy, 2- Rarefying to a depth of 8,000 reads per sample, 3- Removing rare taxa (ASVs present with a total count less than 60 reads across the dataset), 4- Relativized to the total reads per sample after rarfaction (8,000).

1. Data pre-processing
2. FIGURE 2E: Batch variability prior to contaminant removal

```
# Bray-Curtis dissimilarity
set.seed(999)
milk.PCoA.bray5 <- ordinate(data_rarefy_rel_ext, "PCoA", "bray")

pt_ord_m5 <- plot_ordination(data_rarefy_rel_ext, milk.PCoA.bray5)

pt_ord_data_m5 <- as.data.frame(pt_ord_m5$data) # check % variability explained by axes in this object. Just using ggplot directly for final plot instead of phyloseq.

Fig2E <- ggplot(pt_ord_data_m5, aes(Axis.1, Axis.2)) + 
  geom_point(aes(color=Batch), size=2, alpha=0.60) + 
  labs(x="PCoA 1\n(24.3%)",y="PCoA2\n(14.9%)") + 
  ggtitle("") + 
  stat_ellipse(type = "t", linetype = 2, aes(group=Batch, color=Batch)) + 
  scale_color_manual(values=c("#E69F00", "#56B4E9"))+
  theme_minimal() +
  theme(legend.position = "right") 

Fig2E
```

3. ADONIS test for batch effect (not shown in plot, but used in footnote of Table S2)

```
bray <- distance(data_rarefy_rel_ext, method = "bray")
sdf <- as(sample_data(data_rarefy_rel_ext), "data.frame")

set.seed(999)
adonis(bray ~ Batch, data = sdf)
```

```
## 
## Call:
## adonis(formula = bray ~ Batch, data = sdf) 
## 
## Permutation: free
## Number of permutations: 999
## 
## Terms added sequentially (first to last)
## 
##            Df SumsOfSqs MeanSqs F.Model      R2 Pr(>F)    
## Batch       1     59.51  59.510  207.96 0.17897  0.001 ***
## Residuals 954    273.00   0.286         0.82103           
## Total     955    332.51                 1.00000           
## ---
## Signif. codes:  0 '***' 0.001 '**' 0.01 '*' 0.05 '.' 0.1 ' ' 1
```

```
betadisper(bray, sdf$Batch)
```

```
## 
##  Homogeneity of multivariate dispersions
## 
## Call: betadisper(d = bray, group = sdf$Batch)
## 
## No. of Positive Eigenvalues: 384
## No. of Negative Eigenvalues: 571
## 
## Average distance to median:
## Batch1 Batch2 
## 0.4042 0.5721 
## 
## Eigenvalues for PCoA axes:
## (Showing 8 of 955 eigenvalues)
##  PCoA1  PCoA2  PCoA3  PCoA4  PCoA5  PCoA6  PCoA7  PCoA8 
## 80.824 49.605 30.181 18.999 16.819 12.003  9.148  7.913
```

```
permutest(betadisper(bray, sdf$Batch), pairwise = TRUE)
```

```
## 
## Permutation test for homogeneity of multivariate dispersions
## Permutation: free
## Number of permutations: 999
## 
## Response: Distances
##            Df Sum Sq Mean Sq      F N.Perm Pr(>F)    
## Groups      1  6.644  6.6439 190.96    999  0.001 ***
## Residuals 954 33.192  0.0348                         
## ---
## Signif. codes:  0 '***' 0.001 '**' 0.01 '*' 0.05 '.' 0.1 ' ' 1
## 
## Pairwise comparisons:
## (Observed p-value below diagonal, permuted p-value above diagonal)
##            Batch1 Batch2
## Batch1             0.001
## Batch2 1.0025e-39
```

## FIGURE 2F: Batch variability following contamiant identification and removal using decontam

The preprocessing steps performed above are repeated. These include: 1- Rarefying to a depth of 8,000 reads per sample, 3- Removing rare taxa (ASVs present with a total count less than 60 reads across the dataset), 4- Relativized to the total reads per sample after rarfaction (8,000).

1. Data pre-processing
2. FIGURE 2F: Batch variability after decontam

```
#Bray-Curtis dissimilarity
set.seed(999)
milk.PCoA.bray5 <- ordinate(data_rarefy_rel_ext2, "PCoA", "bray")

pt_ord_m5 <- plot_ordination(data_rarefy_rel_ext2, milk.PCoA.bray5)

pt_ord_data_m5 <- as.data.frame(pt_ord_m5$data)

Fig2F <- ggplot(pt_ord_data_m5, aes(Axis.1, Axis.2)) + 
         geom_point(aes(color=Batch), size=2, alpha=0.60) + 
         labs(x="PCoA 1\n(24.4%)",y="PCoA2\n(15.0%)") + 
         ggtitle("") + 
         stat_ellipse(type = "t", linetype = 2, aes(group=Batch, color=Batch)) + 
         scale_color_manual(values=c("#E69F00", "#56B4E9"))+
         theme_minimal() +
         theme(legend.position = "right") 

Fig2F
```

3. ADONIS test for batch effect

```
bray <- distance(data_rarefy_rel_ext2, method = "bray")
sdf <- as(sample_data(data_rarefy_rel_ext2), "data.frame")

set.seed(999)
adonis(bray ~ Batch, data = sdf)
```

```
## 
## Call:
## adonis(formula = bray ~ Batch, data = sdf) 
## 
## Permutation: free
## Number of permutations: 999
## 
## Terms added sequentially (first to last)
## 
##            Df SumsOfSqs MeanSqs F.Model      R2 Pr(>F)    
## Batch       1     59.57  59.567  208.54 0.17938  0.001 ***
## Residuals 954    272.50   0.286         0.82062           
## Total     955    332.07                 1.00000           
## ---
## Signif. codes:  0 '***' 0.001 '**' 0.01 '*' 0.05 '.' 0.1 ' ' 1
```

```
betadisper(bray, sdf$Batch)
```

```
## 
##  Homogeneity of multivariate dispersions
## 
## Call: betadisper(d = bray, group = sdf$Batch)
## 
## No. of Positive Eigenvalues: 381
## No. of Negative Eigenvalues: 574
## 
## Average distance to median:
## Batch1 Batch2 
## 0.4039 0.5715 
## 
## Eigenvalues for PCoA axes:
## (Showing 8 of 955 eigenvalues)
##  PCoA1  PCoA2  PCoA3  PCoA4  PCoA5  PCoA6  PCoA7  PCoA8 
## 80.924 49.732 30.291 19.063 16.848 12.031  9.152  7.897
```

```
permutest(betadisper(bray, sdf$Batch), pairwise = TRUE)
```

```
## 
## Permutation test for homogeneity of multivariate dispersions
## Permutation: free
## Number of permutations: 999
## 
## Response: Distances
##            Df Sum Sq Mean Sq      F N.Perm Pr(>F)    
## Groups      1  6.625  6.6250 190.43    999  0.001 ***
## Residuals 954 33.189  0.0348                         
## ---
## Signif. codes:  0 '***' 0.001 '**' 0.01 '*' 0.05 '.' 0.1 ' ' 1
## 
## Pairwise comparisons:
## (Observed p-value below diagonal, permuted p-value above diagonal)
##            Batch1 Batch2
## Batch1             0.001
## Batch2 1.2477e-39
```

## FIGURE 2G: Batch variability after decontam + considering the data structure i.e. taxa prevalence between the batches and additional contaminant removal

1. FIGURE 2G: Batch variability after decontam and considering the data structure i.e. taxa prevalence between the batches

```
set.seed(999)
milk.PCoA.bray5 <- ordinate(data_rarefy_rel, "PCoA", "bray")

pt_ord_m5 <- plot_ordination(data_rarefy_rel, milk.PCoA.bray5)

pt_ord_data_m5 <- as.data.frame(pt_ord_m5$data)

Fig2G <- ggplot(pt_ord_data_m5, aes(Axis.1, Axis.2)) + 
         geom_point(aes(color=Batch), size=2, alpha=0.60) + 
         labs(x="PCoA 1\n(19.8%)",y="PCoA 2\n(12.1%)") + 
         ggtitle("") + 
         stat_ellipse(type = "t", linetype = 2, aes(group=Batch, color=Batch)) + 
         scale_color_manual(values=c("#E69F00", "#56B4E9"))+
         theme_minimal() + 
         theme(legend.position = "right")

Fig2G
```

2. ADONIS test for batch effect

```
bray <- distance(data_rarefy_rel, method = "bray")
sdf <- as(sample_data(data_rarefy_rel), "data.frame")

set.seed(999)
adonis(bray ~ Batch, data = sdf)
```

```
## 
## Call:
## adonis(formula = bray ~ Batch, data = sdf) 
## 
## Permutation: free
## Number of permutations: 999
## 
## Terms added sequentially (first to last)
## 
##            Df SumsOfSqs MeanSqs F.Model      R2 Pr(>F)    
## Batch       1     4.091  4.0909  12.212 0.01387  0.001 ***
## Residuals 868   290.768  0.3350         0.98613           
## Total     869   294.859                 1.00000           
## ---
## Signif. codes:  0 '***' 0.001 '**' 0.01 '*' 0.05 '.' 0.1 ' ' 1
```

```
betadisper(bray, sdf$Batch)
```

```
## 
##  Homogeneity of multivariate dispersions
## 
## Call: betadisper(d = bray, group = sdf$Batch)
## 
## No. of Positive Eigenvalues: 325
## No. of Negative Eigenvalues: 544
## 
## Average distance to median:
## Batch1 Batch2 
## 0.5450 0.5705 
## 
## Eigenvalues for PCoA axes:
## (Showing 8 of 869 eigenvalues)
##  PCoA1  PCoA2  PCoA3  PCoA4  PCoA5  PCoA6  PCoA7  PCoA8 
## 58.254 35.816 24.441 21.057 12.672 10.547  9.553  8.130
```

```
permutest(betadisper(bray, sdf$Batch), pairwise = TRUE)
```

```
## 
## Permutation test for homogeneity of multivariate dispersions
## Permutation: free
## Number of permutations: 999
## 
## Response: Distances
##            Df  Sum Sq  Mean Sq      F N.Perm Pr(>F)  
## Groups      1  0.1347 0.134662 5.9958    999  0.013 *
## Residuals 868 19.4948 0.022459                       
## ---
## Signif. codes:  0 '***' 0.001 '**' 0.01 '*' 0.05 '.' 0.1 ' ' 1
## 
## Pairwise comparisons:
## (Observed p-value below diagonal, permuted p-value above diagonal)
##          Batch1 Batch2
## Batch1           0.015
## Batch2 0.014537
```

# Assessment of repeatability and reproducibility of the results

## FIGURE 3A: Comparison of milk microbiota taxonomy between the two batches

1. Dataset preprocessing

```
#MELT datasets
physeq2_milk2_m <- psmelt(data_rarefy_rel)

## remove the prefixes from the taxonomy file
physeq2_milk2_m$Phylum <- gsub("^p__", "", physeq2_milk2_m$Phylum)
physeq2_milk2_m$Class <- gsub("^c__", "", physeq2_milk2_m$Class)
physeq2_milk2_m$Family <- gsub("^f__", "", physeq2_milk2_m$Family)
physeq2_milk2_m$Genus <- gsub("^g__", "", physeq2_milk2_m$Genus)

#This new Column is as Unique as ASV level (yet to decide which taxonomic level to color plot by)
physeq2_milk2_m$OTU_Phylum_Class_Fam_Genus <- paste(physeq2_milk2_m$Phylum, physeq2_milk2_m$Class, physeq2_milk2_m$Family, physeq2_milk2_m$Genus, physeq2_milk2_m$OTU, sep = ":")
     

#data summary - Plot Dependent, will plot average abundance of ASVs per-batch
physeq2_milk_summ <- data_summary(physeq2_milk2_m, varname = "Abundance", groupnames = c("Batch", "OTU_Phylum_Class_Fam_Genus"))
physeq2_milk_summ_delim <- physeq2_milk_summ %>% separate(OTU_Phylum_Class_Fam_Genus, 
                c("Phylum", "Class", "Family", "Genus", "OTU"), sep = ":")
```

2. FIGURE 3A. stacked bar chart of Family-level taxonomic plot of the top 50 most abundant ASVs.

```
col_blind18 <- c("cadetblue", "darkgoldenrod1",
"dodgerblue4","indianred3", "navajowhite2", "mistyrose3", "lightsteelblue3", "lightgoldenrod1", "deepskyblue4", "cornsilk3", "black", "grey")

#Check which abundant taxa to plot vs. which to clump into "Other" category
ASVs_Top <- names(sort(taxa_sums(data_rarefy_rel), TRUE)[1:25]) # plot top 25 ASVs
datafr2_Top <- prune_taxa(ASVs_Top, data_rarefy_rel)
top_class <- get_taxa_unique(datafr2_Top, taxonomic.rank = "Family") # To be plotted, remaining family level taxonomy shown as "Other"
top_class <- gsub("^f__", "", top_class)

physeq2_milk_summ_delim$Main_Class <- physeq2_milk_summ_delim$Family #physeq2_milk_summ_delim is the table to be plotted 
physeq2_milk_summ_delim$Main_Class <- ifelse(physeq2_milk_summ_delim$Main_Class %in% top_class, physeq2_milk_summ_delim$Main_Class, "Other")


# post-hoc addition, it seems we want to plot all taxa (even those rare taxa in <60 reads filtered upstream) as "Other"
physeq2_milk_summ_delim_B1 <- subset(physeq2_milk_summ_delim, Batch == "Batch1", select = c("Batch", "Abundance", "Main_Class")) # only picking plotted columns
sum(physeq2_milk_summ_delim_B1$Abundance) # want an additional "Other" group that makes this sum to 100
```

```
## [1] 98.68112
```

```
add_row <- c("Batch1", 100-sum(physeq2_milk_summ_delim_B1$Abundance), "Other")
add_row
```

```
## [1] "Batch1"           "1.31887982195846" "Other"
```

```
physeq2_milk_summ_delim_B2 <- subset(physeq2_milk_summ_delim, Batch == "Batch2", select = c("Batch", "Abundance", "Main_Class"))
sum(physeq2_milk_summ_delim_B2$Abundance)
```

```
## [1] 99.27488
```

```
add_row2 <- c("Batch2", 100-sum(physeq2_milk_summ_delim_B2$Abundance), "Other")
add_row2
```

```
## [1] "Batch2"            "0.725117260787997" "Other"
```

```
## re-merge and plot again
physeq2_milk_summ_delim2 <- rbind(physeq2_milk_summ_delim_B1, add_row, physeq2_milk_summ_delim_B2, add_row2)
physeq2_milk_summ_delim2$Abundance <- as.numeric(as.character(physeq2_milk_summ_delim2$Abundance))

physeq2_milk_summ_delim2$Main_Class <- as.factor(physeq2_milk_summ_delim2$Main_Class)
levels(physeq2_milk_summ_delim2$Main_Class) # Top 50 ASVs, to many taxa to plot, dropped to top 25 ASVS - encompassess 12 Families including "Other"
```

```
##  [1] "Bacillaceae"        "Enterobacteriaceae" "Gemellaceae"       
##  [4] "Micrococcaceae"     "Moraxellaceae"      "Other"             
##  [7] "Pasteurellaceae"    "Pseudomonadaceae"   "Staphylococcaceae" 
## [10] "Streptococcaceae"   "Veillonellaceae"    "Xanthomonadaceae"
```

```
physeq2_milk_summ_delim2$Main_Class = ordered(physeq2_milk_summ_delim2$Main_Class, levels=c("Bacillaceae","Enterobacteriaceae","Gemellaceae","Micrococcaceae" ,"Moraxellaceae","Pasteurellaceae", "Pseudomonadaceae","Staphylococcaceae","Streptococcaceae","Veillonellaceae","Xanthomonadaceae", "Other"))


labs = c(expression(italic("Bacillaceae")), expression(italic("Enterobacteriaceae")),expression(italic("Gemellaceae")), expression(italic("Micrococcaceae")), expression(italic("Moraxellaceae")), expression(italic("Pasteurellaceae")), expression(italic("Pseudomonadaceae")), expression(italic("Staphylococcaceae")), expression(italic("Streptococcaceae")), expression(italic("Veillonellaceae")), expression(italic("Xanthomonadaceae")), "Other")


Fig3A <- ggplot(physeq2_milk_summ_delim2, aes(x=Batch, y=Abundance, fill=Main_Class, colour=Main_Class))+
         geom_bar(stat="identity", position="stack") + 
         scale_color_manual(name = "Family", values = c(col_blind18), labels=labs) +
         scale_y_continuous(expand=c(0,0)) + 
         labs(y="Relative Abundance (%)") + 
         theme_minimal()+
         scale_fill_manual(name = "Family", values = c(col_blind18), labels=labs) +
         theme(axis.ticks.x = element_blank(), axis.title.x = element_blank(), legend.text.align = 0) + 
         ylim(0,100)+
         xlab("") 
Fig3A
```

# FIGURE 3B: Assessment of repeatability and reproducibility of statistical associations in original Batch 1, new Batch 1, and Batch 2

## Make table of associations for each batch separately

Only the analysis for Batch 2 is shown. The same approach was repeated for the Original Batch 1 and New Batch 1.

1. Frist make data subset for Batch 2

```
data_relative <- subset_samples(data_rarefy_rel, Batch == "Batch2")
#Filter taxa with average mean relative abundance of less thann 0.01%
datafr2 = filter_taxa(data_relative, function(x) mean(x) > 1e-2, TRUE) 
nsamples(datafr2)
```

```
## [1] 533
```

```
# Exporting data from phyloseq object
data_vegan <- veganotu(datafr2)
sdf <- as(sample_data(datafr2), "data.frame")
tax <- as(tax_table(datafr2), "matrix")

# Replace 0 values with an estimate of the probability that the zero is not 0

set.seed(14701)
d.n0 <- cmultRepl(data_vegan,  label=0, method="CZM", output="p-counts")
```

```
## No. corrected values:  96974
```

```
#Centered log-ratio transformation (CLR). Function from codaSeq, equivalent to log(x/gx) for every value where gx is the geomtric mean of the vector X

d.n0.clr <- codaSeq.clr(d.n0, samples.by.row=TRUE)

# Create the phyloseq object with centered log-ratio transformed ASV table

otumat_clr <- as.matrix(t(d.n0.clr))
OTU = otu_table(otumat_clr, taxa_are_rows=TRUE)
TAX = tax_table(tax)
sampledata = sample_data(sdf)
newPhyseq_clr = phyloseq(OTU, TAX, sampledata)
newPhyseq_clr
```

```
## phyloseq-class experiment-level object
## otu_table()   OTU Table:         [ 230 taxa and 533 samples ]
## sample_data() Sample Data:       [ 533 samples by 148 sample variables ]
## tax_table()   Taxonomy Table:    [ 230 taxa by 7 taxonomic ranks ]
```

2. Univariate redundancy analyses of determinants of milk microbiota (Moossavi et al. Cell Host Microbe 2019) with the overall milk microbiota composition. Factors that are assessed: Maternal antibiotics before 3-4 months; Child antibiotics before 3-4 months; Maternal secretor status; Total HMO concentration; Maternal BMI; Mode of delivary; Lactation stage; Exclusive breastfeeding; Number of older siblings; Mode of breastfeeding; HMO composition profile

The process is shown for one of the factors. The same approach was repeated for other variables.

```
set.seed(14701)
```

Maternal antibiotics, 3-4 months

```
#Exluding samples with missing observation for maternal antibiotics
m <- newPhyseq_clr %>%
  subset_samples(Mom_abs_3mo_IV_oral_supp_yn != "NA")
nsamples(m)
```

```
## [1] 515
```

```
#Export ASV table as matrix
data <- veganotu(m)
#Export metadata as dataframe
sdf_m <- as(sample_data(m), "data.frame")
table(sdf_m$Mom_abs_3mo_IV_oral_supp_yn)
```

```
## 
##  No Yes 
## 452  63
```

```
mat_abs_RDA <- rda(data ~sdf_m$Mom_abs_3mo_IV_oral_supp_yn)
mat_abs_ano <- anova(mat_abs_RDA) 
mat_abs_R2 <- RsquareAdj(mat_abs_RDA)$r.squared
mat_abs_R2
```

```
## [1] 0.003013087
```

3. Combine the RDA results for Batch 2 into a summary table

```
# Compile P values
p_values_B2 <- c(HMO_compAno$`Pr(>F)`[1],
                 BM_mode_ano$`Pr(>F)`[1],
                 olderSibs_ano$`Pr(>F)`[1],
                 ebf_ano$`Pr(>F)`[1],
                 lac_ano$`Pr(>F)`[1],
                 mode_ano$`Pr(>F)`[1],
                 BMI_ano$`Pr(>F)`[1],
                 HMO_ano$`Pr(>F)`[1],
                 sec_ano$`Pr(>F)`[1],
                 child_ano$`Pr(>F)`[1],
                 mat_abs_ano$`Pr(>F)`[1])

# Compile R2's
R2s_B2 <- c(HMO_compR2,
            BM_mode_R2,
            olderSibs_R2,
            ebf_R2,
            lac_R2,
            mode_R2,
            BMI_R2,
            HMO_R2,
            sec_R2,
            child_R2,
            mat_abs_R2)


names <- c("HMO composition profile", "Mode of breastfeeding", "Number of older siblings", "Exclusive breastfeeding", "Lactation stage", "Mode of delivery", "Maternal BMI", "Total HMO concentration", "Maternal secretor status", "Child antibiotics before 3-4 months", "Maternal antibiotics before 3-4 months") # same for batch 2 and batch 1


Univar_B2 <- rbind(name=names, R2=R2s_B2, P_value=p_values_B2)

Univar_B2 <- as.data.frame(t(Univar_B2))

Univar_B2$Batch <- "Batch 2"


Univar_B2$R2 <- as.numeric(as.character(Univar_B2$R2))
Univar_B2$P_value <- as.numeric(as.character(Univar_B2$P_value))

#P-value cats
Univar_B2$P_value_cat[Univar_B2$P_value<=0.001] <- "***" # 0.001 is actually max p-value for the RDA
Univar_B2$P_value_cat[Univar_B2$P_value<0.01 & Univar_B2$P_value>0.001] <- "**"
Univar_B2$P_value_cat[Univar_B2$P_value<0.05 & Univar_B2$P_value>=0.01] <- "*"
Univar_B2$P_value_cat[Univar_B2$P_value<0.1 & Univar_B2$P_value>=0.05] <- "~"
Univar_B2$P_value_cat[Univar_B2$P_value>=0.1] <- ""
```

4. Combine the RDA results for Batch 1 into a summary table

```
# Compile P values
p_values_B1 <- c(HMO_compAno$`Pr(>F)`[1],
                 BM_mode_ano$`Pr(>F)`[1],
                 olderSibs_ano$`Pr(>F)`[1],
                 ebf_ano$`Pr(>F)`[1],
                 lac_ano$`Pr(>F)`[1],
                 mode_ano$`Pr(>F)`[1],
                 BMI_ano$`Pr(>F)`[1],
                 HMO_ano$`Pr(>F)`[1],
                 sec_ano$`Pr(>F)`[1],
                 child_ano$`Pr(>F)`[1],
                 mat_abs_ano$`Pr(>F)`[1])

# Compile R2's
R2s_B1 <- c(HMO_compR2,
            BM_mode_R2,
            olderSibs_R2,
            ebf_R2,
            lac_R2,
            mode_R2,
            BMI_R2,
            HMO_R2,
            sec_R2,
            child_R2,
            mat_abs_R2)


names <- c("HMO composition profile", "Mode of breastfeeding", "Number of older siblings", "Exclusive breastfeeding", "Lactation stage", "Mode of delivery", "Maternal BMI", "Total HMO concentration", "Maternal secretor status", "Child antibiotics before 3-4 months", "Maternal antibiotics before 3-4 months") # same for batch 2 and batch 1
length(names)
```

```
## [1] 11
```

```
Univar_B1 <- rbind(name=names, R2=R2s_B1, P_value=p_values_B1)

Univar_B1 <- as.data.frame(t(Univar_B1))

Univar_B1$Batch <- "Batch 1 - new dataset"


Univar_B1$R2 <- as.numeric(as.character(Univar_B1$R2))
Univar_B1$P_value <- as.numeric(as.character(Univar_B1$P_value))

#P-value cats
Univar_B1$P_value_cat[Univar_B1$P_value<=0.001] <- "***" # 0.001 is actually max p-value for the RDA
Univar_B1$P_value_cat[Univar_B1$P_value<0.01 & Univar_B1$P_value>0.001] <- "**"
Univar_B1$P_value_cat[Univar_B1$P_value<0.05 & Univar_B1$P_value>=0.01] <- "*"
Univar_B1$P_value_cat[Univar_B1$P_value<0.1 & Univar_B1$P_value>=0.05] <- "~"
Univar_B1$P_value_cat[Univar_B1$P_value>=0.1] <- ""
```

## Tabel of associations for Original Batch 1 dataset

Preprocessing of this dataset and the redundancy analysis is described in Moossavi, et al. Cell Host & Microbe 2019. The RDA analyses done above follow this same methods.

1. Import table - data from all dyads (n=393) Figure 3 in https://doi.org/10.1016/j.chom.2019.01.011

```
Original_B1 <- read.csv(file="input_data/RDA_Fig3_Moossavi2019CHM.csv", header=TRUE)
```

## FIGURE 3B: Comparison of the statistical associations of determinants of the milk microbiota composition using redundancy analysis.

1. Merge tables of RDA results and transform R2 to redundancy values or percent of explained variation

```
Univar_data_all <- rbind(Univar_B2,Univar_B1,Original_B1)

# R2 in percent
Univar_data_all$Redundancy_value <- Univar_data_all$R2*100
```

2. FIGURE 3B: Comparison of the statistical associations of determinants of the milk microbiota composition using redundancy analysis

```
Univar_data_all$name <- as.factor(Univar_data_all$name)
Univar_data_all$Batch <- factor(Univar_data_all$Batch, levels = c("Batch 1 - original dataset", "Batch 1 - new dataset", "Batch 2"))

Fig3B <- ggplot(data=Univar_data_all, aes(x= reorder(name, -Redundancy_value), y= Redundancy_value)) +
         geom_bar(stat="identity", position=position_dodge(), aes(fill=Batch))+
         theme(panel.grid.major = element_blank(), panel.grid.minor = element_blank())+
         labs(colour = NULL)+
         ylab("Redundancy value (%)")+ xlab("")+
         theme(axis.title=element_text(size=10))+
         coord_flip()+
         guides(fill = guide_legend(reverse=T, title=NULL))+
         theme(panel.grid.major = element_blank(), panel.grid.minor = element_blank())+
         scale_fill_manual(values=c("#999999", "#E69F00", "#56B4E9"))+
         theme_minimal()+
  theme(legend.position = "right")

Fig3B
```

3. Additional table of the p-value categories and redundancy values plotted in Figure 3B

```
Univar_data_all2 <- Univar_data_all
Univar_data_all2$P_value <- NULL
Univar_data_all2$R2 <- NULL
Univar_data_all2$Batch <- gsub(" dataset", "", Univar_data_all2$Batch)

Univar_data_all2
```

```
##                                      name              Batch P_value_cat
## 1                 HMO composition profile            Batch 2           *
## 2                   Mode of breastfeeding            Batch 2         ***
## 3                Number of older siblings            Batch 2         ***
## 4                 Exclusive breastfeeding            Batch 2         ***
## 5                         Lactation stage            Batch 2         ***
## 6                        Mode of delivery            Batch 2            
## 7                            Maternal BMI            Batch 2           *
## 8                 Total HMO concentration            Batch 2            
## 9                Maternal secretor status            Batch 2            
## 10    Child antibiotics before 3-4 months            Batch 2            
## 11 Maternal antibiotics before 3-4 months            Batch 2           *
## 12                HMO composition profile      Batch 1 - new           *
## 13                  Mode of breastfeeding      Batch 1 - new         ***
## 14               Number of older siblings      Batch 1 - new         ***
## 15                Exclusive breastfeeding      Batch 1 - new          **
## 16                        Lactation stage      Batch 1 - new         ***
## 17                       Mode of delivery      Batch 1 - new            
## 18                           Maternal BMI      Batch 1 - new           *
## 19                Total HMO concentration      Batch 1 - new           ~
## 20               Maternal secretor status      Batch 1 - new            
## 21    Child antibiotics before 3-4 months      Batch 1 - new            
## 22 Maternal antibiotics before 3-4 months      Batch 1 - new            
## 23                           Maternal BMI Batch 1 - original           *
## 24               Maternal secretor status Batch 1 - original           ~
## 25                       Mode of delivery Batch 1 - original            
## 26               Number of older siblings Batch 1 - original           *
## 27    Child antibiotics before 3-4 months Batch 1 - original            
## 28 Maternal antibiotics before 3-4 months Batch 1 - original            
## 29                        Lactation stage Batch 1 - original          **
## 30                  Mode of breastfeeding Batch 1 - original         ***
## 31                Exclusive breastfeeding Batch 1 - original         ***
## 32                Total HMO concentration Batch 1 - original            
## 33                HMO composition profile Batch 1 - original           ~
##    Redundancy_value
## 1         4.1221426
## 2         0.6657114
## 3         0.8165988
## 4         0.5911532
## 5         0.5633162
## 6         0.3759562
## 7         0.3511385
## 8         0.2379255
## 9         0.1195245
## 10        0.1705454
## 11        0.3013087
## 12        6.3410617
## 13        1.2026980
## 14        1.1463526
## 15        0.7540525
## 16        0.8656840
## 17        0.6168461
## 18        0.4668015
## 19        0.4167951
## 20        0.3225909
## 21        0.3852520
## 22        0.2125261
## 23        0.4300000
## 24        0.3800000
## 25        0.6200000
## 26        0.8400000
## 27        0.2500000
## 28        0.2100000
## 29        0.5900000
## 30        1.3400000
## 31        0.9300000
## 32        0.3100000
## 33        5.5000000
```

```
sessionInfo()
```

```
## R version 3.5.2 (2018-12-20)
## Platform: x86_64-apple-darwin15.6.0 (64-bit)
## Running under: macOS High Sierra 10.13.6
## 
## Matrix products: default
## BLAS: /Library/Frameworks/R.framework/Versions/3.5/Resources/lib/libRblas.0.dylib
## LAPACK: /Library/Frameworks/R.framework/Versions/3.5/Resources/lib/libRlapack.dylib
## 
## locale:
## [1] en_CA.UTF-8/en_CA.UTF-8/en_CA.UTF-8/C/en_CA.UTF-8/en_CA.UTF-8
## 
## attached base packages:
## [1] stats     graphics  grDevices utils     datasets  methods   base     
## 
## other attached packages:
##  [1] ggcorrplot_0.1.3    Hmisc_4.3-1         Formula_1.2-3      
##  [4] plyr_1.8.6          irr_0.84.1          lpSolve_5.6.15     
##  [7] CoDaSeq_0.99.3      car_3.0-8           carData_3.0-4      
## [10] ALDEx2_1.14.1       zCompositions_1.3.4 truncnorm_1.0-8    
## [13] NADA_1.6-1.1        survival_3.2-3      MASS_7.3-51.6      
## [16] vegan_2.5-6         lattice_0.20-41     permute_0.9-5      
## [19] RColorBrewer_1.1-2  decontam_1.2.1      reshape2_1.4.4     
## [22] forcats_0.5.0       stringr_1.4.0       dplyr_1.0.0        
## [25] purrr_0.3.4         readr_1.3.1         tidyr_1.1.0        
## [28] tibble_3.0.3        tidyverse_1.3.0     ggplot2_3.3.2      
## [31] phyloseq_1.26.1    
## 
## loaded via a namespace (and not attached):
##   [1] colorspace_1.4-1            ellipsis_0.3.1             
##   [3] rio_0.5.16                  htmlTable_2.0.1            
##   [5] XVector_0.22.0              base64enc_0.1-3            
##   [7] GenomicRanges_1.34.0        fs_1.4.2                   
##   [9] rstudioapi_0.11             farver_2.0.3               
##  [11] fansi_0.4.1                 lubridate_1.7.9            
##  [13] xml2_1.3.2                  codetools_0.2-16           
##  [15] splines_3.5.2               knitr_1.29                 
##  [17] ade4_1.7-15                 jsonlite_1.7.0             
##  [19] broom_0.7.0                 cluster_2.1.0              
##  [21] dbplyr_1.4.4                compiler_3.5.2             
##  [23] httr_1.4.2                  backports_1.1.8            
##  [25] assertthat_0.2.1            Matrix_1.2-18              
##  [27] cli_2.0.2                   acepack_1.4.1              
##  [29] htmltools_0.5.0             tools_3.5.2                
##  [31] igraph_1.2.5                gtable_0.3.0               
##  [33] glue_1.4.1                  GenomeInfoDbData_1.2.0     
##  [35] Rcpp_1.0.5                  Biobase_2.42.0             
##  [37] cellranger_1.1.0            vctrs_0.3.2                
##  [39] Biostrings_2.50.2           multtest_2.38.0            
##  [41] ape_5.3                     nlme_3.1-138               
##  [43] iterators_1.0.13            xfun_0.15                  
##  [45] openxlsx_4.1.5              rvest_0.3.5                
##  [47] lifecycle_0.2.0             zlibbioc_1.28.0            
##  [49] scales_1.1.1                hms_0.5.3                  
##  [51] parallel_3.5.2              SummarizedExperiment_1.12.0
##  [53] biomformat_1.10.1           rhdf5_2.26.2               
##  [55] yaml_2.2.1                  curl_4.3                   
##  [57] gridExtra_2.3               rpart_4.1-15               
##  [59] latticeExtra_0.6-28         stringi_1.4.6              
##  [61] S4Vectors_0.20.1            foreach_1.5.1              
##  [63] checkmate_2.0.0             BiocGenerics_0.28.0        
##  [65] zip_2.0.4                   BiocParallel_1.16.6        
##  [67] GenomeInfoDb_1.18.2         rlang_0.4.7                
##  [69] pkgconfig_2.0.3             bitops_1.0-6               
##  [71] matrixStats_0.56.0          evaluate_0.14              
##  [73] Rhdf5lib_1.4.3              htmlwidgets_1.5.1          
##  [75] labeling_0.3                tidyselect_1.1.0           
##  [77] magrittr_1.5                R6_2.4.1                   
##  [79] IRanges_2.16.0              generics_0.0.2             
##  [81] DelayedArray_0.8.0          DBI_1.1.0                  
##  [83] pillar_1.4.6                haven_2.3.1                
##  [85] foreign_0.8-71              withr_2.2.0                
##  [87] mgcv_1.8-31                 nnet_7.3-14                
##  [89] abind_1.4-5                 RCurl_1.98-1.2             
##  [91] modelr_0.1.8                crayon_1.3.4               
##  [93] rmarkdown_2.3               grid_3.5.2                 
##  [95] readxl_1.3.1                data.table_1.12.8          
##  [97] blob_1.2.1                  reprex_0.3.0               
##  [99] digest_0.6.25               stats4_3.5.2               
## [101] munsell_0.5.0
```
